# Supplementary material for: Macrophages form dendrite-like pseudopods to enhance bacterial ingestion
Source: EMBO J. 2025 Jul 28;44(17):4772–802. doi: 10.1038/s44318-025-00515-z (PMC12402336; doi:10.1038/s44318-025-00515-z)
Supplement: Supplementary file 1 — Appendix [file 44318_2025_515_MOESM1_ESM.pdf]

Appendix for **"Macrophages form dendrite-like pseudopods to enhance bacterial ingestion"**

Changyuan Fan[1,2,3], Xinyi Huang[1,2], Jie Mei[1,2,3], Xuemeng Shi[2], Hao Zhang[4], Cong Liang[4], Shuzhi Cui[2], Yifan Xing[2], Biao Cao[5], Wei Liu[6], Huisheng Liu[7], Bo Liu[1,2,3], Wakam Chang[8], Mengle Shao[1,2,3], Gong-Hong Wei[9], Yan-Jun Liu[6], Zheng-Jun Chen[4], Zhaoyu Lin[10,*], Tao Xu[3,7,*], Yaming Jiu[1,2,3,*]

[1] Shanghai Institute of Materia Medica, Chinese Academy of Sciences, Shanghai 201203, China

[2] Shanghai Institute of Immunity and Infection, Chinese Academy of Sciences, Shanghai 200031, China

[3] University of Chinese Academy of Sciences, Yuquan Road No. 19(A), Shijingshan District, Beijing 100049, China

[4] State Key Laboratory of Cell Biology, Shanghai Institute of Biochemistry and Cell Biology, Center for Excellence in Molecular Cell Science, Chinese Academy of Sciences, Shanghai 200031, China

[5] State Key Laboratory of Experimental Hematology, Shanghai Institute of Nutrition and Health, Chinese Academy of Sciences, Shanghai 200031, China.

[6] Shanghai Xuhui Central Hospital, Zhongshan-Xuhui Hospital, Shanghai Key Laboratory of Medical Epigenetics, Shanghai Stomatological Hospital, Institutes of Biomedical Sciences, Department of Chemistry, Fudan University, Shanghai 200032, China.

[7] Guangzhou Laboratory, Guangzhou 510005, China

[8] Department of Biomedical Sciences, Faculty of Health Sciences, University of Macau, Taipa, Macau 999074, China

[9] MOE Key Laboratory of Metabolism and Molecular Medicine & Department of Biochemistry and Molecular Biology of School of Basic Medical Sciences, and Fudan University Shanghai Cancer Center, Cancer Institutes, Department of Oncology, Shanghai Medical College of Fudan University, Shanghai 200032,

China.
[10] State Key Laboratory of Pharmaceutical Biotechnology, Ministry of Education Key Laboratory of Model Animal for Disease Study, Jiangsu Key Laboratory of Molecular Medicine, Model Animal Research Center, National Resource Center for Mutant Mice of China, Nanjing Drum Tower Hospital, School of Medicine, Nanjing University, Nanjing 210061, China

Table of Contents

Table of Contents	Page
Appendix Figure S1	1

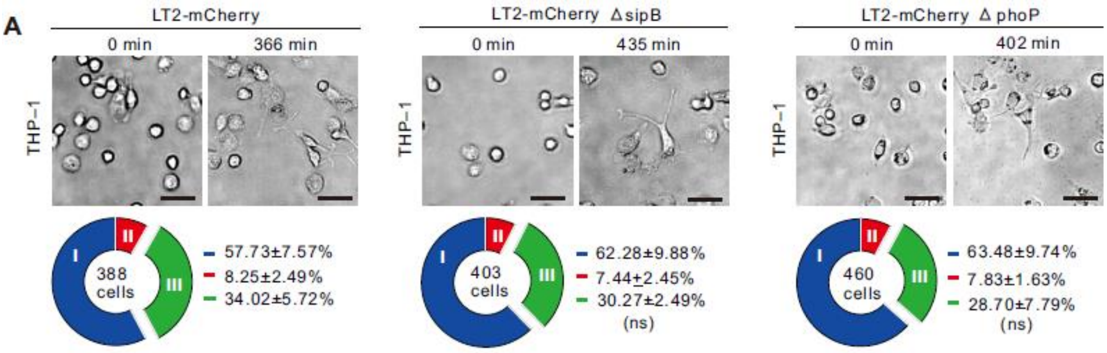

Appendix Figure S1. (A) Time-lapse imaging of THP-1 macrophages showing the DLPs formation upon *Salmonella* infection (LT2-mCherry WT / ΔsipB / ΔphoP). Scale bars, 20 μm. pie chart quantification of the percentage of three macrophage shapes in THP-1 macrophages infected with different *Salmonella* strains (MOI=20). Data are presented as mean ± s.d. from three independent experiments. ns P > 0.05. one-way ANOVA with Sidak's analysis (A).
