# Supplementary material for: Macrophages form dendrite-like pseudopods to enhance bacterial ingestion
Source: EMBO J. 2025 Jul 28;44(17):4772–802. doi: 10.1038/s44318-025-00515-z (PMC12402336; doi:10.1038/s44318-025-00515-z)
Supplement: Supplementary file 3 — Movie EV1 [file 44318_2025_515_MOESM3_ESM.zip › Movie EV1.docx]

**Movie EV1.** Time-lapse two-photon intravital microscope of peritoneal macrophages stained with anti-F4/80 with or without *Salmonella*, related to Fig. 1A-D. Images were displayed every 10 min for 70 min. Arrows denoted the pseudopods. Scale bar, 20 µm.
