# Supplementary material for: Macrophages form dendrite-like pseudopods to enhance bacterial ingestion
Source: EMBO J. 2025 Jul 28;44(17):4772–802. doi: 10.1038/s44318-025-00515-z (PMC12402336; doi:10.1038/s44318-025-00515-z)
Supplement: Supplementary file 5 — Movie EV3 [file 44318_2025_515_MOESM5_ESM.zip › Movie EV3.docx]

**Movie EV 3.** Time-lapse brightfield video of THP-1 macrophages infected with or without *Salmonella* in microfluidics, related to Fig. 2D. Images were displayed every 30 s for 70 min and 48 min, arrows denoted DLPs. Scale bar, 20 µm.
