# Supplementary material for: Macrophages form dendrite-like pseudopods to enhance bacterial ingestion
Source: EMBO J. 2025 Jul 28;44(17):4772–802. doi: 10.1038/s44318-025-00515-z (PMC12402336; doi:10.1038/s44318-025-00515-z)
Supplement: Supplementary file 6 — Movie EV4 [file 44318_2025_515_MOESM6_ESM.zip › Movie EV4.docx]

**Movie EV 4.** Time-lapse confocal imaging video of *Salmonella* infected THP-1 cells visualized by SiR-actin, related to Fig. 3C. Images were displayed every 6 min for 120 min. Scale bar, 10 µm.
