# Supplementary material for: Macrophages form dendrite-like pseudopods to enhance bacterial ingestion
Source: EMBO J. 2025 Jul 28;44(17):4772–802. doi: 10.1038/s44318-025-00515-z (PMC12402336; doi:10.1038/s44318-025-00515-z)
Supplement: Supplementary file 7 — Movie EV5 [file 44318_2025_515_MOESM7_ESM.zip › Movie EV5.docx]

**Movie EV 5.** Time-lapse confocal imaging video of *Salmonella* infected VIM KO THP-1 cells visualized by SiR-actin, related to Fig. 3J. Images were displayed every 5 min for 595 min. Yellow dash line denoted the actin-rich region. Scale bar, 20 µm.
