# Supplementary material for: Macrophages form dendrite-like pseudopods to enhance bacterial ingestion
Source: EMBO J. 2025 Jul 28;44(17):4772–802. doi: 10.1038/s44318-025-00515-z (PMC12402336; doi:10.1038/s44318-025-00515-z)
Supplement: Supplementary file 8 — Movie EV6 [file 44318_2025_515_MOESM8_ESM.zip › Movie EV6.docx]

**Movie EV 6.** Time-lapse brightfield imaging video of THP-1 macrophages infected with *Salmonella* upon LTA or LPS treatment, related to Fig. 4B. Images were displayed every 3 min for 500 min. Scale bar, 20 µm.
