# Supplementary material for: Macrophages form dendrite-like pseudopods to enhance bacterial ingestion
Source: EMBO J. 2025 Jul 28;44(17):4772–802. doi: 10.1038/s44318-025-00515-z (PMC12402336; doi:10.1038/s44318-025-00515-z)
Supplement: Supplementary file 9 — Movie EV7 [file 44318_2025_515_MOESM9_ESM.zip › Movie EV7.docx]

**Movie EV7.** Time-lapse confocal imaging video of mCherry-expressing *Salmonella* infected THP-1 macrophages visualized by Alexa 488 WGA, related to Fig. 6B. Images were acquired every 4 min for 188 min. Arrows denoted an internalized *Salmonella*, and a dash line indicated the border between cell body and DLPs. Scale bar, 20 µm.
