# Supplementary figures and images for: Macrophages form dendrite-like pseudopods to enhance bacterial ingestion (part 1 of 3)
Source: EMBO J. 2025 Jul 28;44(17):4772–802. doi: 10.1038/s44318-025-00515-z (PMC12402336; doi:10.1038/s44318-025-00515-z)

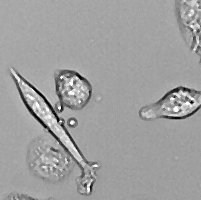

Supplement: Supplementary file 10 — Source data Fig. 1 [file 44318_2025_515_MOESM10_ESM.zip › Figure1/1G/Mock-80 min-1.tif]

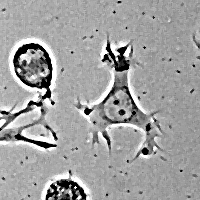

Supplement: Supplementary file 10 — Source data Fig. 1 [file 44318_2025_515_MOESM10_ESM.zip › Figure1/1G/Salmonella-80 min-1.tif]

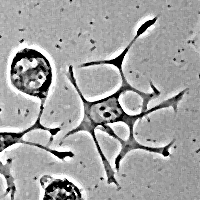

Supplement: Supplementary file 10 — Source data Fig. 1 [file 44318_2025_515_MOESM10_ESM.zip › Figure1/1G/Salmonella-120 min-1.tif]

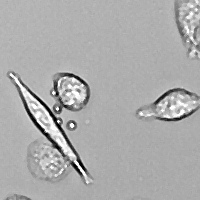

Supplement: Supplementary file 10 — Source data Fig. 1 [file 44318_2025_515_MOESM10_ESM.zip › Figure1/1G/Mock-0 min-1.tif]

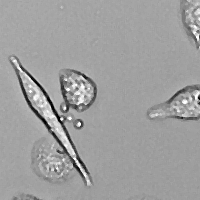

Supplement: Supplementary file 10 — Source data Fig. 1 [file 44318_2025_515_MOESM10_ESM.zip › Figure1/1G/Mock-120 min-1.tif]

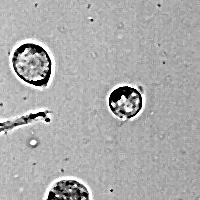

Supplement: Supplementary file 10 — Source data Fig. 1 [file 44318_2025_515_MOESM10_ESM.zip › Figure1/1G/Salmonella-0 min-1.tif]

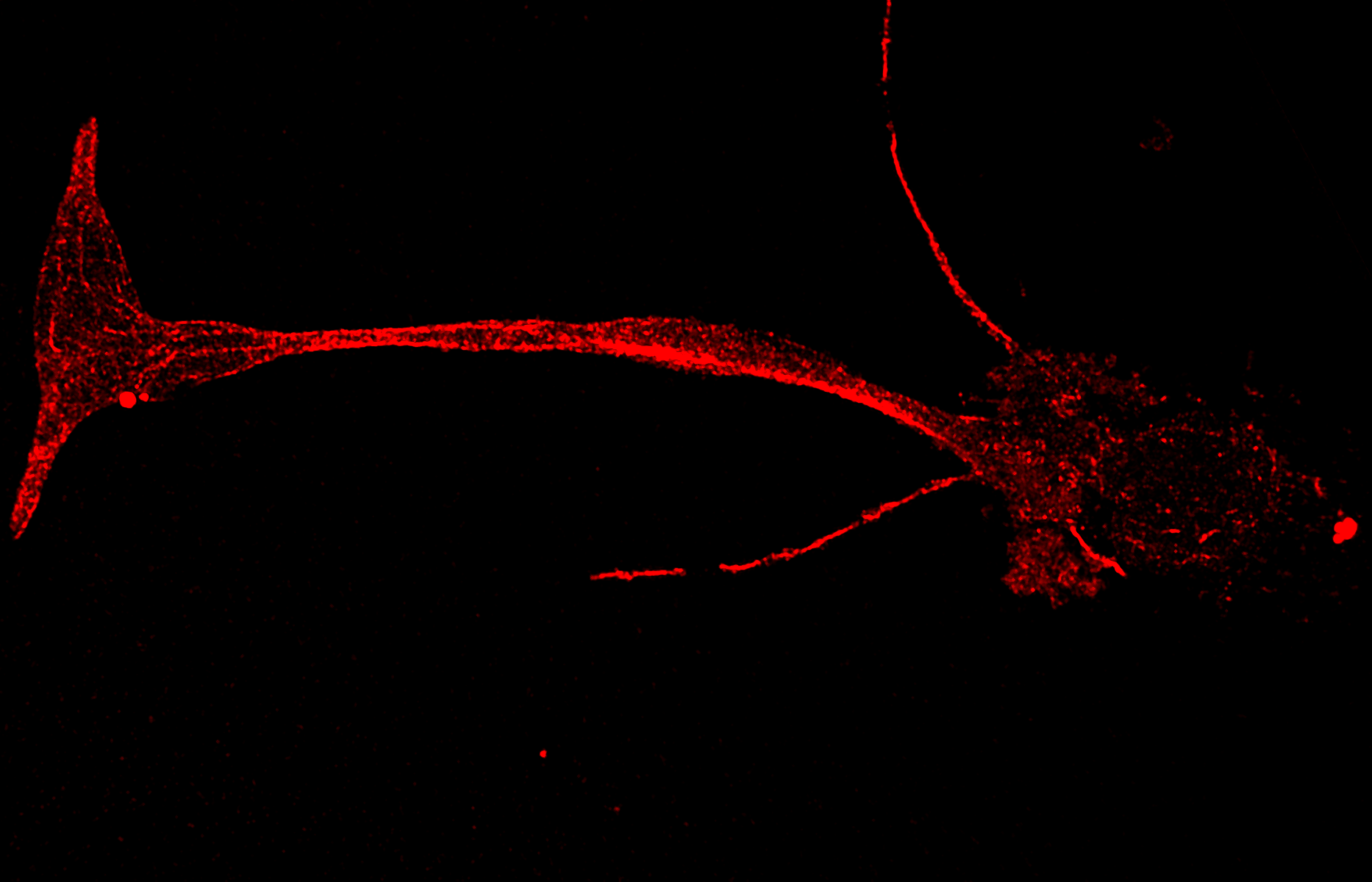

Supplement: Supplementary file 10 — Source data Fig. 1 [file 44318_2025_515_MOESM10_ESM.zip › Figure1/1N/WGA.tif]

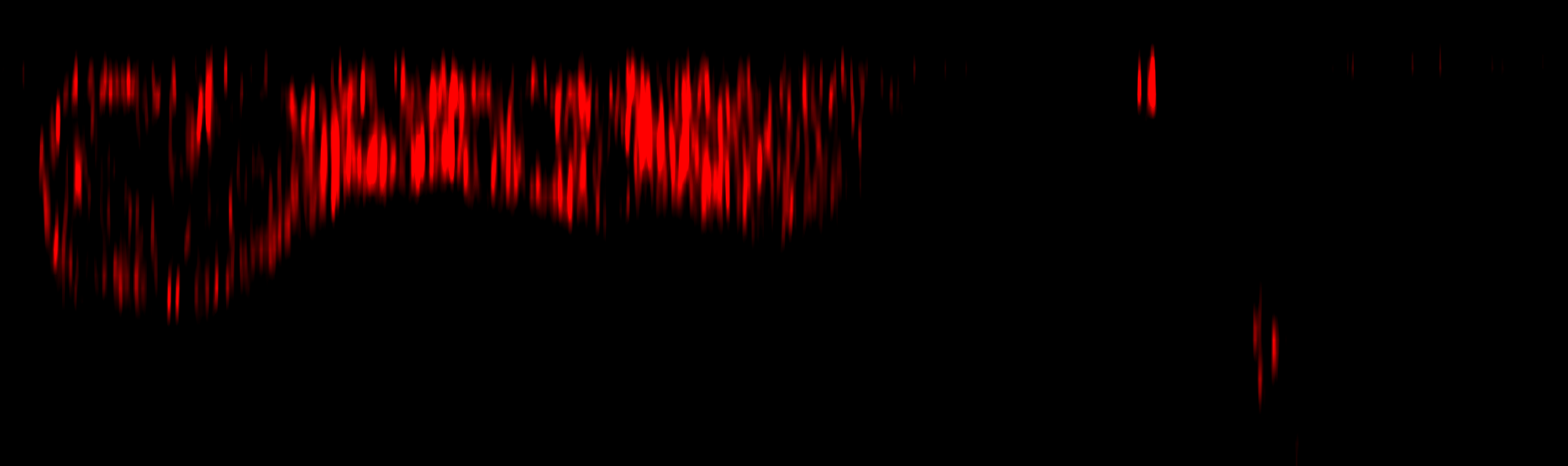

Supplement: Supplementary file 10 — Source data Fig. 1 [file 44318_2025_515_MOESM10_ESM.zip › Figure1/1N/Composite.tif]

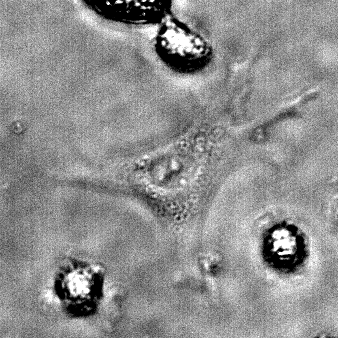

Supplement: Supplementary file 10 — Source data Fig. 1 [file 44318_2025_515_MOESM10_ESM.zip › Figure1/1L/PEM 129min.tif]

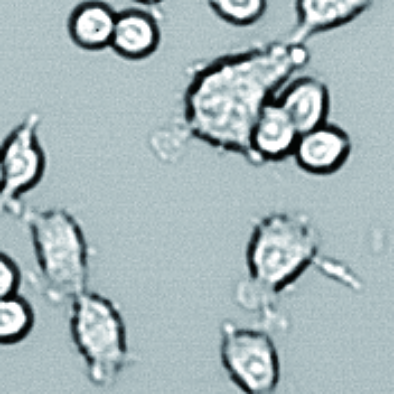

Supplement: Supplementary file 10 — Source data Fig. 1 [file 44318_2025_515_MOESM10_ESM.zip › Figure1/1L/iBMDM 0min.tif]

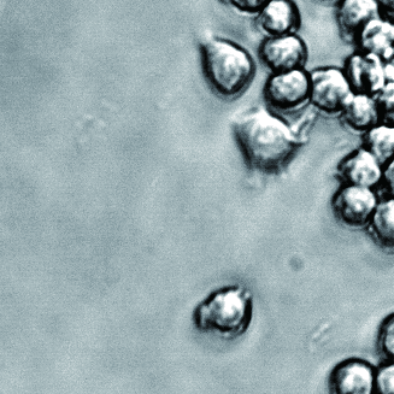

Supplement: Supplementary file 10 — Source data Fig. 1 [file 44318_2025_515_MOESM10_ESM.zip › Figure1/1L/RAW264.7 0min.tif]

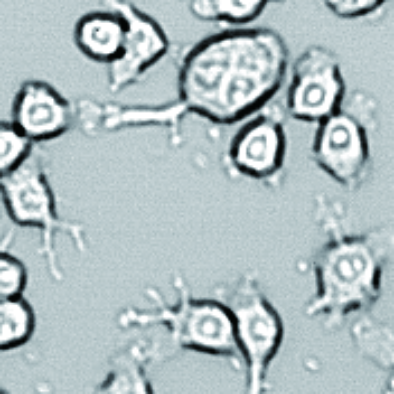

Supplement: Supplementary file 10 — Source data Fig. 1 [file 44318_2025_515_MOESM10_ESM.zip › Figure1/1L/iBMDM 62min.tif]

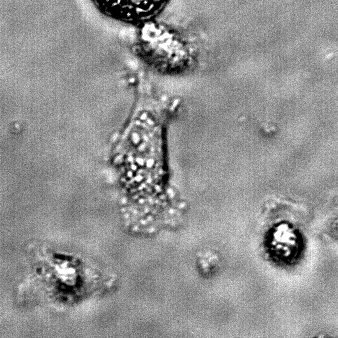

Supplement: Supplementary file 10 — Source data Fig. 1 [file 44318_2025_515_MOESM10_ESM.zip › Figure1/1L/PEM 0min.tif]

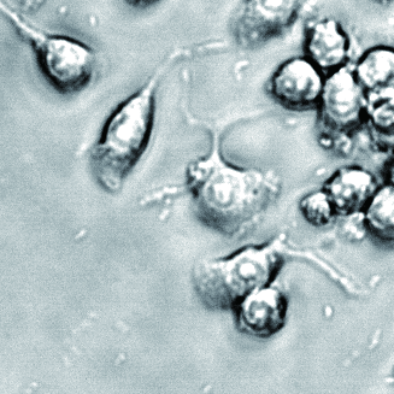

Supplement: Supplementary file 10 — Source data Fig. 1 [file 44318_2025_515_MOESM10_ESM.zip › Figure1/1L/RAW264.7 174min.tif]

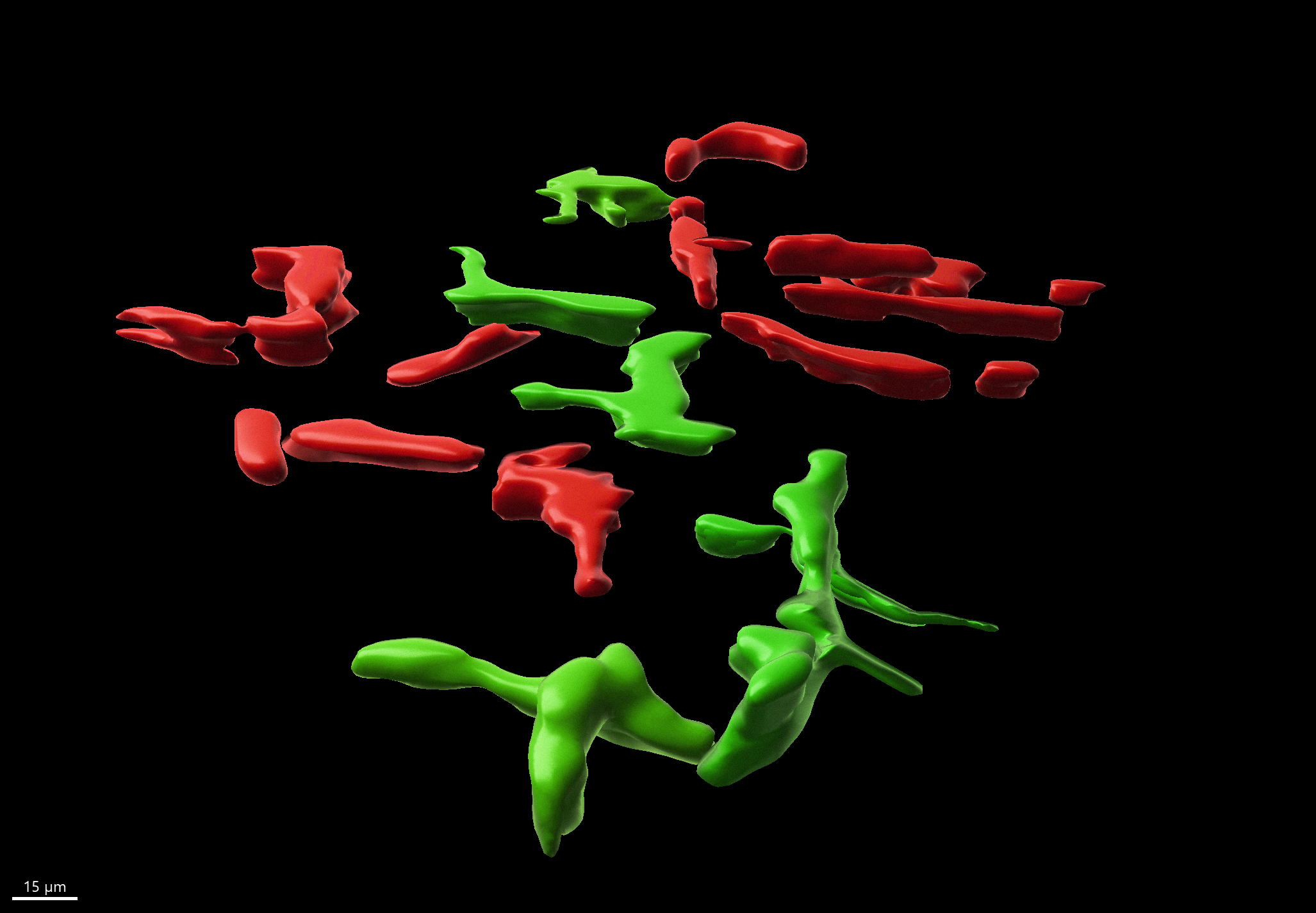

Supplement: Supplementary file 10 — Source data Fig. 1 [file 44318_2025_515_MOESM10_ESM.zip › Figure1/1D/Salmonella 3d.tif]

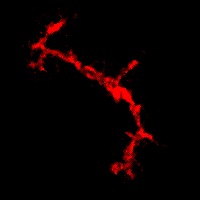

Supplement: Supplementary file 10 — Source data Fig. 1 [file 44318_2025_515_MOESM10_ESM.zip › Figure1/1D/Salmonella Exp1.tif]

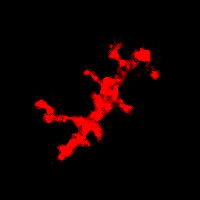

Supplement: Supplementary file 10 — Source data Fig. 1 [file 44318_2025_515_MOESM10_ESM.zip › Figure1/1D/Salmoenlla Exp2.tif]

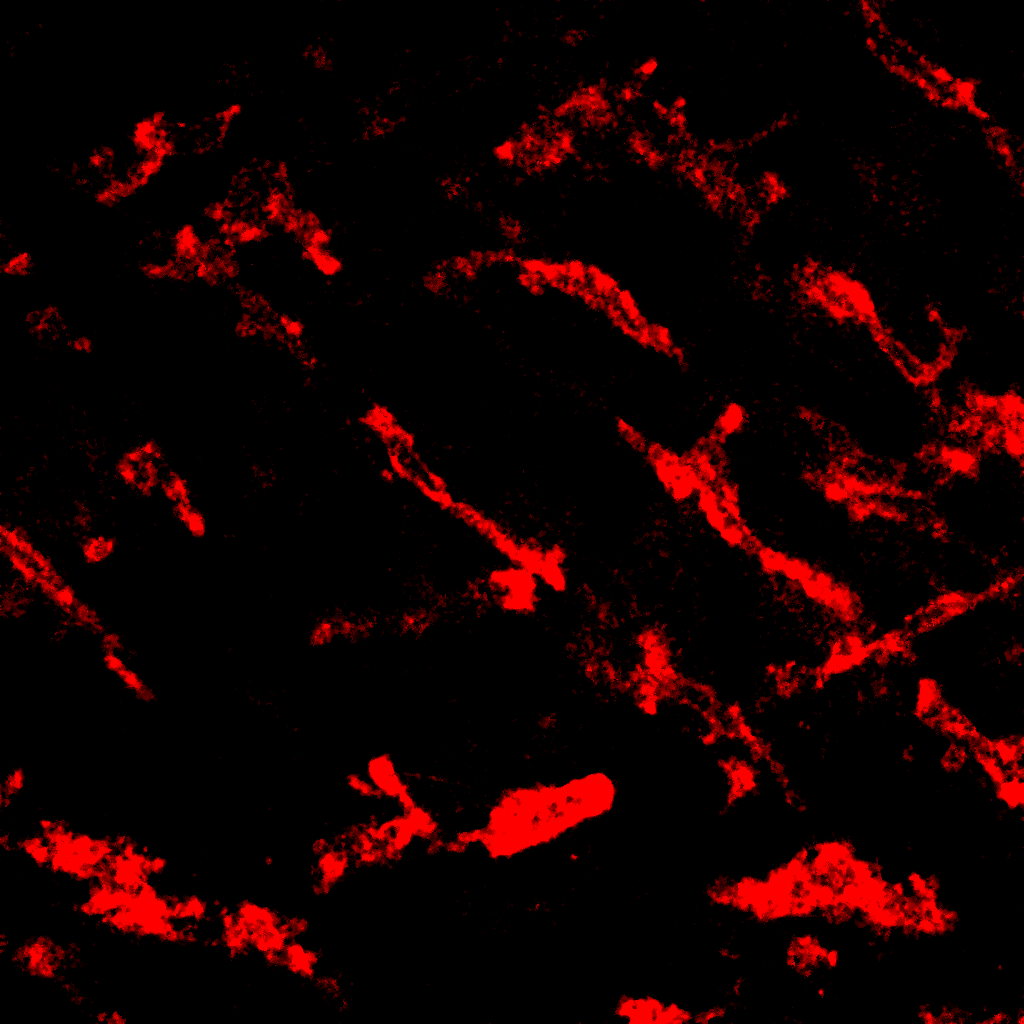

Supplement: Supplementary file 10 — Source data Fig. 1 [file 44318_2025_515_MOESM10_ESM.zip › Figure1/1D/Mock.tif]

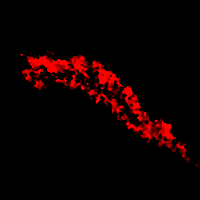

Supplement: Supplementary file 10 — Source data Fig. 1 [file 44318_2025_515_MOESM10_ESM.zip › Figure1/1D/Mock Exp1.tif]

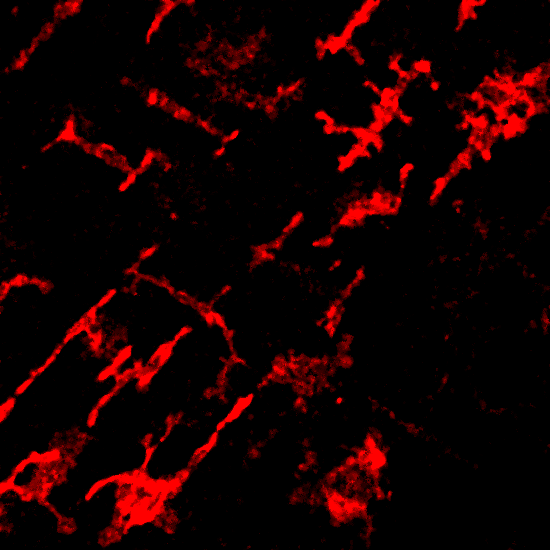

Supplement: Supplementary file 10 — Source data Fig. 1 [file 44318_2025_515_MOESM10_ESM.zip › Figure1/1D/Salmonella.tif]

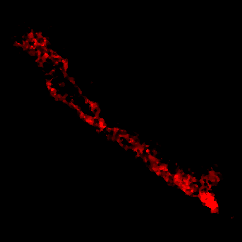

Supplement: Supplementary file 10 — Source data Fig. 1 [file 44318_2025_515_MOESM10_ESM.zip › Figure1/1D/Mock Exp2.tif]

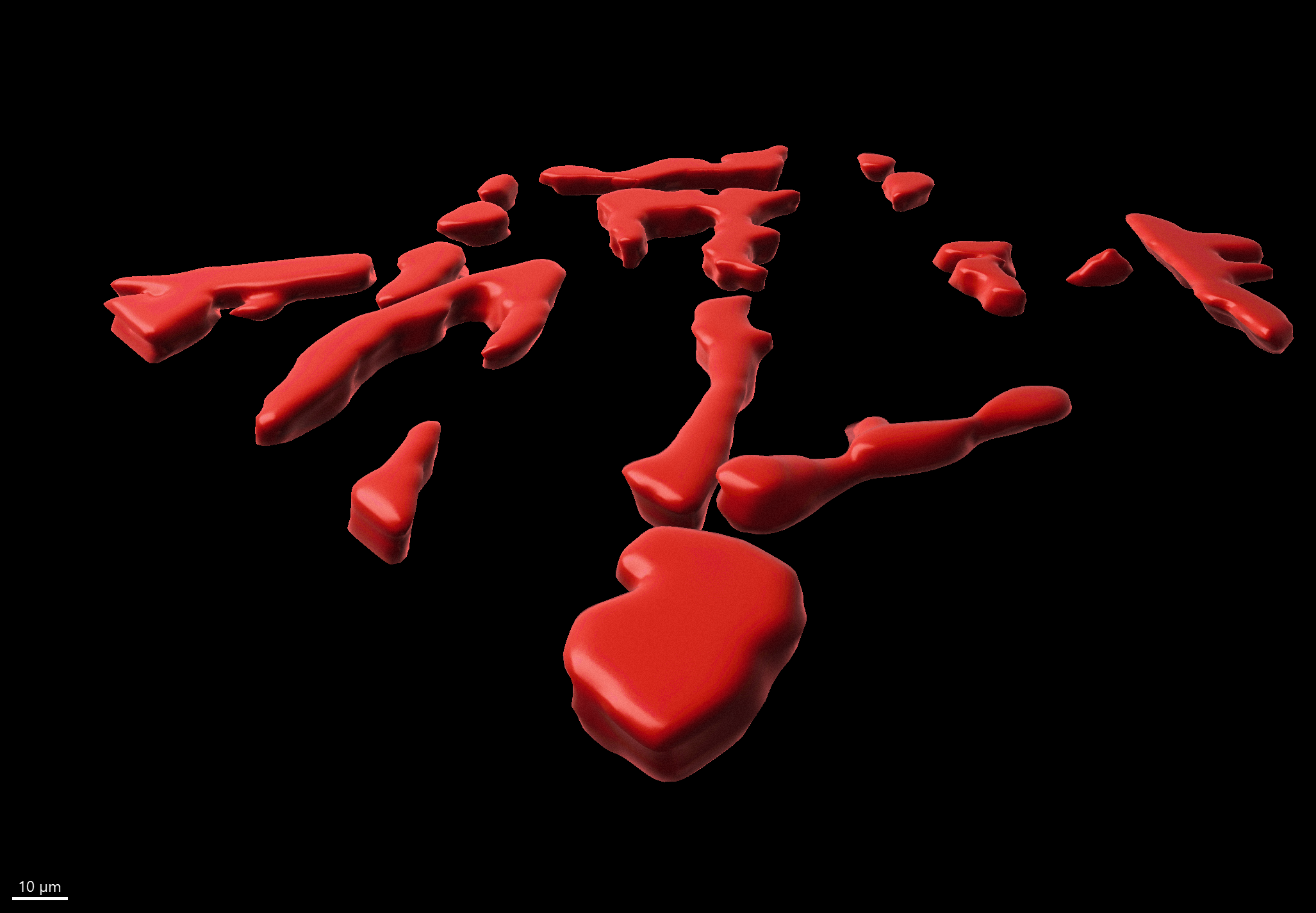

Supplement: Supplementary file 10 — Source data Fig. 1 [file 44318_2025_515_MOESM10_ESM.zip › Figure1/1D/Mock 3d.tif]

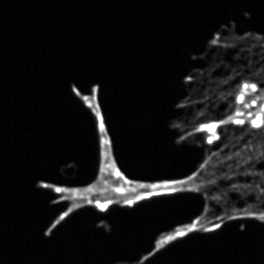

Supplement: Supplementary file 10 — Source data Fig. 1 [file 44318_2025_515_MOESM10_ESM.zip › Figure1/1M/DLP RAW data.tif]

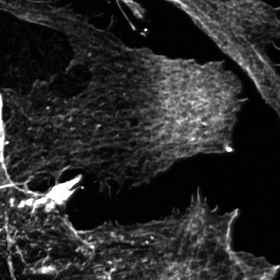

Supplement: Supplementary file 10 — Source data Fig. 1 [file 44318_2025_515_MOESM10_ESM.zip › Figure1/1M/lamenipodia RAW data.tif]

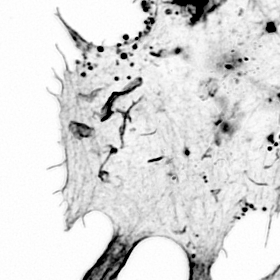

Supplement: Supplementary file 10 — Source data Fig. 1 [file 44318_2025_515_MOESM10_ESM.zip › Figure1/1M/filopodia.tif]

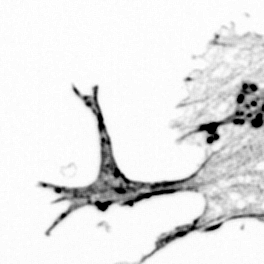

Supplement: Supplementary file 10 — Source data Fig. 1 [file 44318_2025_515_MOESM10_ESM.zip › Figure1/1M/DLP.tif]

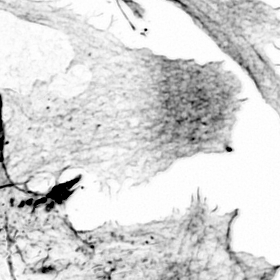

Supplement: Supplementary file 10 — Source data Fig. 1 [file 44318_2025_515_MOESM10_ESM.zip › Figure1/1M/lamenipodia.tif]

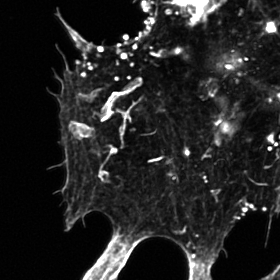

Supplement: Supplementary file 10 — Source data Fig. 1 [file 44318_2025_515_MOESM10_ESM.zip › Figure1/1M/filopodia RAW data.tif]

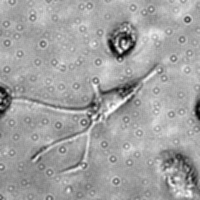

Supplement: Supplementary file 10 — Source data Fig. 1 [file 44318_2025_515_MOESM10_ESM.zip › Figure1/1Q/retraction 0h.tif]

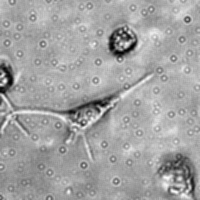

Supplement: Supplementary file 10 — Source data Fig. 1 [file 44318_2025_515_MOESM10_ESM.zip › Figure1/1Q/retraction 6h.tif]

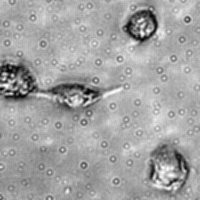

Supplement: Supplementary file 10 — Source data Fig. 1 [file 44318_2025_515_MOESM10_ESM.zip › Figure1/1Q/retraction 14h.tif]

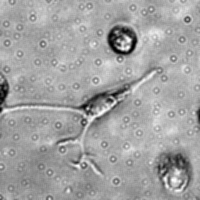

Supplement: Supplementary file 10 — Source data Fig. 1 [file 44318_2025_515_MOESM10_ESM.zip › Figure1/1Q/retraction 4h.tif]

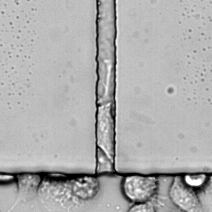

Supplement: Supplementary file 11 — Source data Fig. 2 [file 44318_2025_515_MOESM11_ESM.zip › Figure2/2B/Salmonella-RGB.tif]

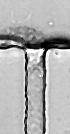

Supplement: Supplementary file 11 — Source data Fig. 2 [file 44318_2025_515_MOESM11_ESM.zip › Figure2/2D/Mock-0 min.tif]

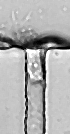

Supplement: Supplementary file 11 — Source data Fig. 2 [file 44318_2025_515_MOESM11_ESM.zip › Figure2/2D/Mock-100 min.tif]

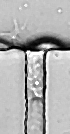

Supplement: Supplementary file 11 — Source data Fig. 2 [file 44318_2025_515_MOESM11_ESM.zip › Figure2/2D/Mock-274 min.tif]

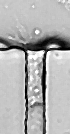

Supplement: Supplementary file 11 — Source data Fig. 2 [file 44318_2025_515_MOESM11_ESM.zip › Figure2/2D/Mock-365 min.tif]

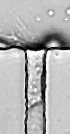

Supplement: Supplementary file 11 — Source data Fig. 2 [file 44318_2025_515_MOESM11_ESM.zip › Figure2/2D/Mock-430 min.tif]

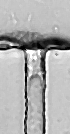

Supplement: Supplementary file 11 — Source data Fig. 2 [file 44318_2025_515_MOESM11_ESM.zip › Figure2/2D/Mock-69 min.tif]

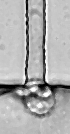

Supplement: Supplementary file 11 — Source data Fig. 2 [file 44318_2025_515_MOESM11_ESM.zip › Figure2/2D/Salmonella-0 min.tif]

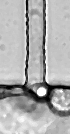

Supplement: Supplementary file 11 — Source data Fig. 2 [file 44318_2025_515_MOESM11_ESM.zip › Figure2/2D/Salmonella-167 min.tif]

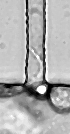

Supplement: Supplementary file 11 — Source data Fig. 2 [file 44318_2025_515_MOESM11_ESM.zip › Figure2/2D/Salmonella-230 min.tif]

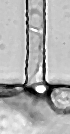

Supplement: Supplementary file 11 — Source data Fig. 2 [file 44318_2025_515_MOESM11_ESM.zip › Figure2/2D/Salmonella-251 min.tif]

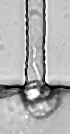

Supplement: Supplementary file 11 — Source data Fig. 2 [file 44318_2025_515_MOESM11_ESM.zip › Figure2/2D/Salmonella-41 min.tif]

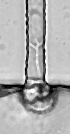

Supplement: Supplementary file 11 — Source data Fig. 2 [file 44318_2025_515_MOESM11_ESM.zip › Figure2/2D/Salmonella-59 min.tif]

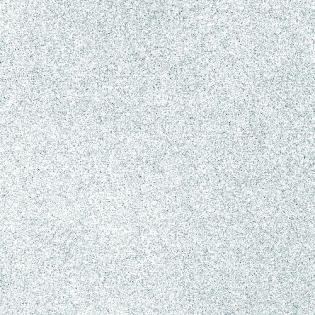

Supplement: Supplementary file 11 — Source data Fig. 2 [file 44318_2025_515_MOESM11_ESM.zip › Figure2/2J/Plane A pores 0.4um.tif]

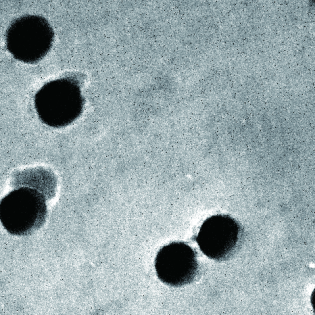

Supplement: Supplementary file 11 — Source data Fig. 2 [file 44318_2025_515_MOESM11_ESM.zip › Figure2/2J/Plane A pores 8um.tif]

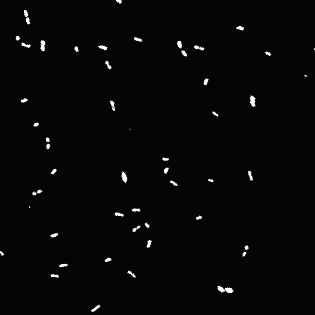

Supplement: Supplementary file 11 — Source data Fig. 2 [file 44318_2025_515_MOESM11_ESM.zip › Figure2/2J/Plane A salmonella 0.4um.tif]

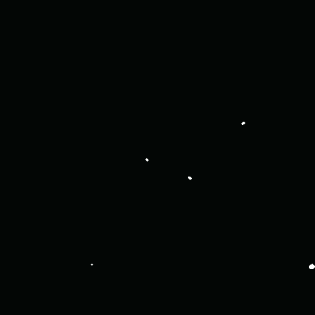

Supplement: Supplementary file 11 — Source data Fig. 2 [file 44318_2025_515_MOESM11_ESM.zip › Figure2/2J/Plane A salmonella 8um.tif]

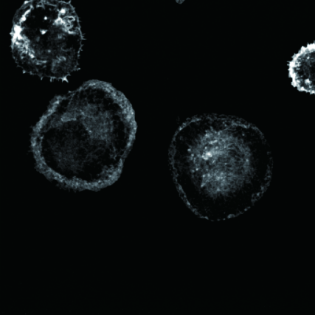

Supplement: Supplementary file 11 — Source data Fig. 2 [file 44318_2025_515_MOESM11_ESM.zip › Figure2/2J/Plane B Cell 0.4um.tif]

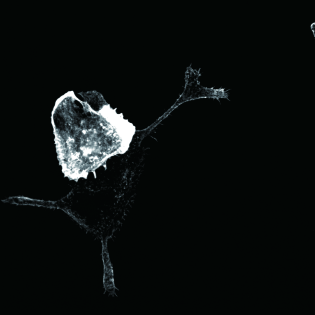

Supplement: Supplementary file 11 — Source data Fig. 2 [file 44318_2025_515_MOESM11_ESM.zip › Figure2/2J/Plane B Cell 8um.tif]

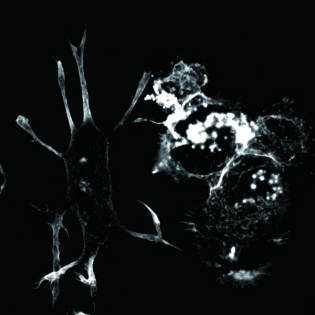

Supplement: Supplementary file 11 — Source data Fig. 2 [file 44318_2025_515_MOESM11_ESM.zip › Figure2/2J/Plane B Cell no pore.tif]

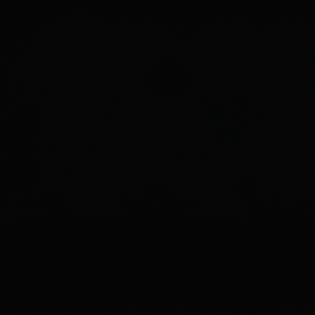

Supplement: Supplementary file 11 — Source data Fig. 2 [file 44318_2025_515_MOESM11_ESM.zip › Figure2/2J/Plane B salmonella 0.4um.tif]

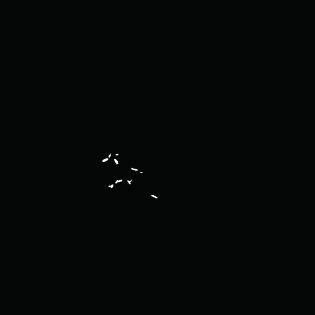

Supplement: Supplementary file 11 — Source data Fig. 2 [file 44318_2025_515_MOESM11_ESM.zip › Figure2/2J/Plane B salmonella 8um.tif]

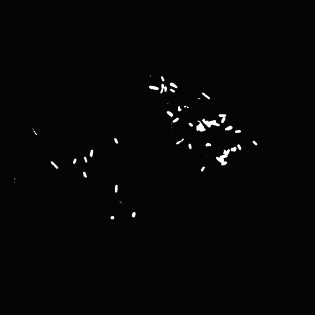

Supplement: Supplementary file 11 — Source data Fig. 2 [file 44318_2025_515_MOESM11_ESM.zip › Figure2/2J/Plane B salmonella no pore.tif]

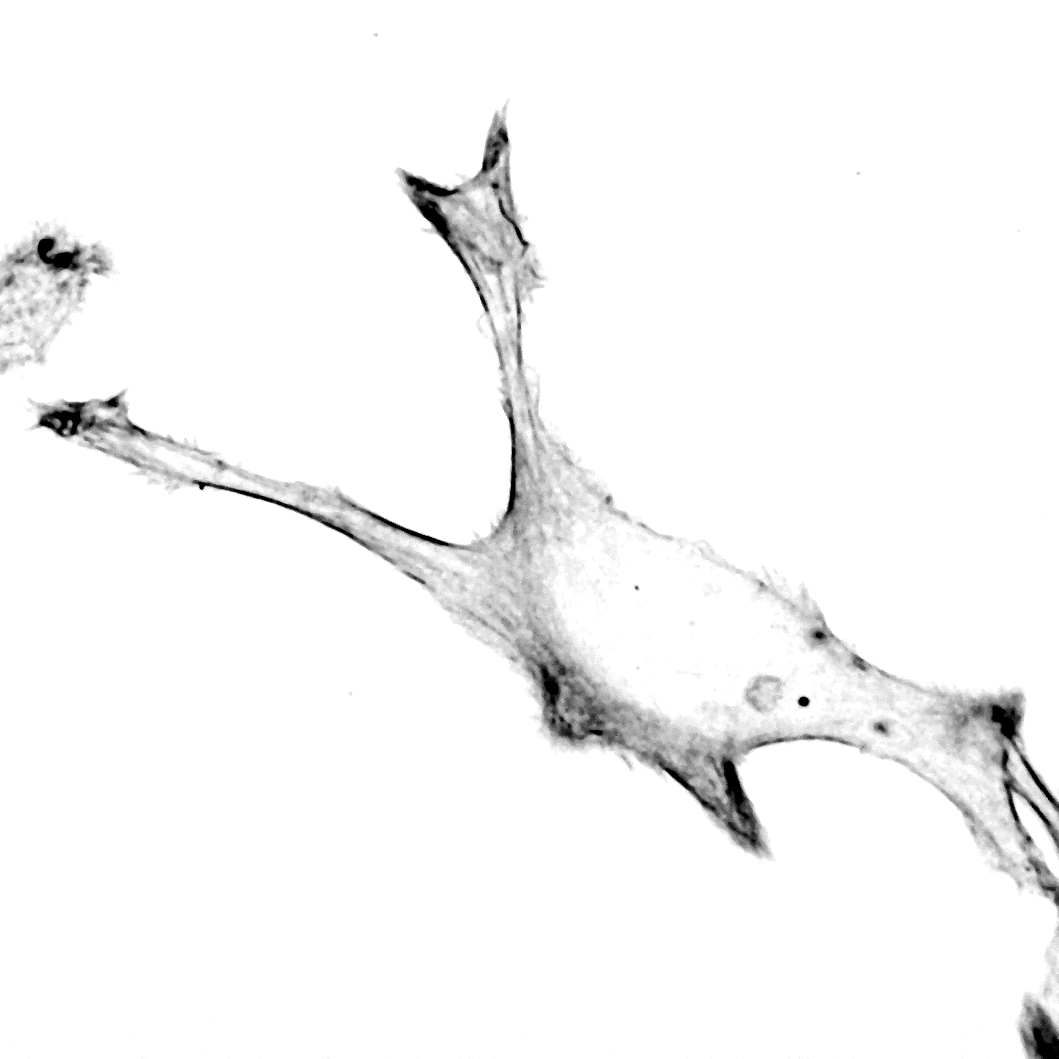

Supplement: Supplementary file 12 — Source data Fig. 3 [file 44318_2025_515_MOESM12_ESM.zip › Figure3/3A/actin example.tif]

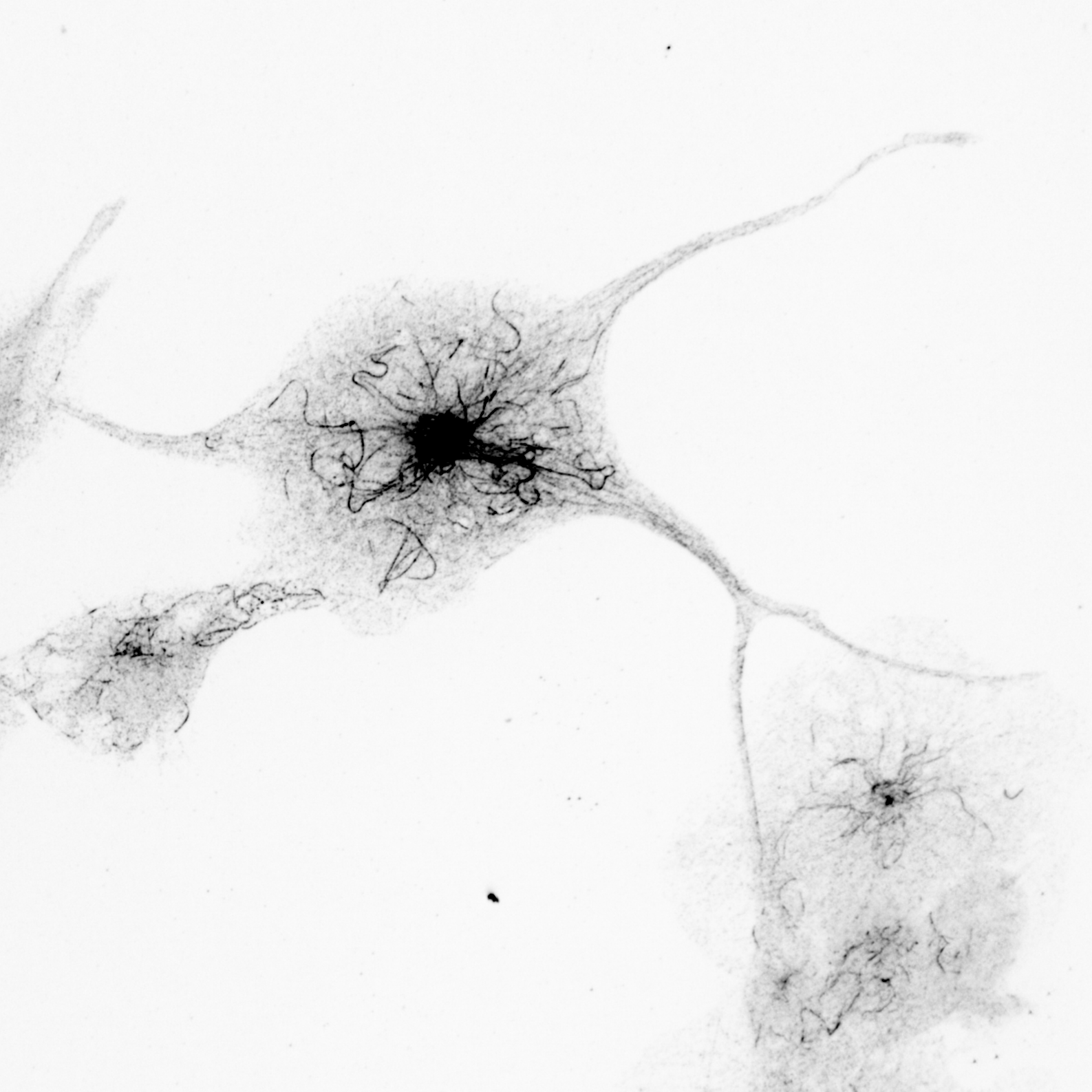

Supplement: Supplementary file 12 — Source data Fig. 3 [file 44318_2025_515_MOESM12_ESM.zip › Figure3/3A/tubulin example.tif]

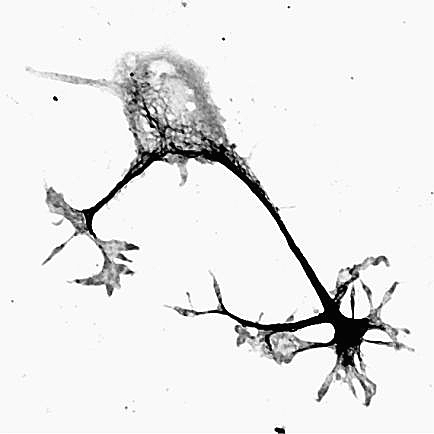

Supplement: Supplementary file 12 — Source data Fig. 3 [file 44318_2025_515_MOESM12_ESM.zip › Figure3/3A/vimentin example.tif]

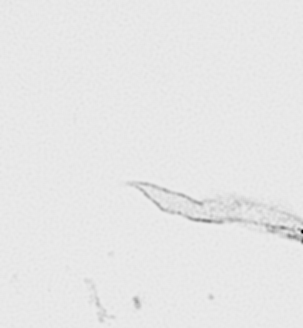

Supplement: Supplementary file 12 — Source data Fig. 3 [file 44318_2025_515_MOESM12_ESM.zip › Figure3/3C/0min.tif]

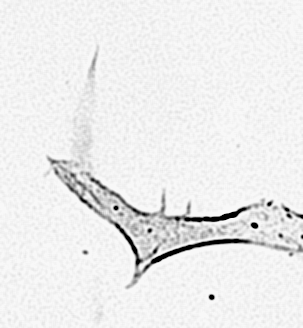

Supplement: Supplementary file 12 — Source data Fig. 3 [file 44318_2025_515_MOESM12_ESM.zip › Figure3/3C/102min.tif]

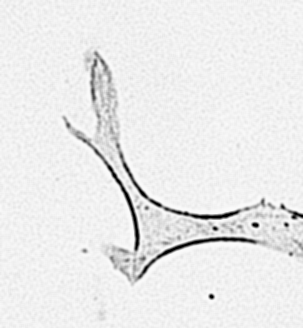

Supplement: Supplementary file 12 — Source data Fig. 3 [file 44318_2025_515_MOESM12_ESM.zip › Figure3/3C/114min.tif]

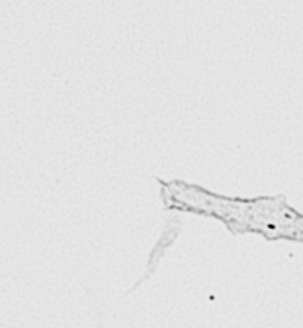

Supplement: Supplementary file 12 — Source data Fig. 3 [file 44318_2025_515_MOESM12_ESM.zip › Figure3/3C/18min.tif]

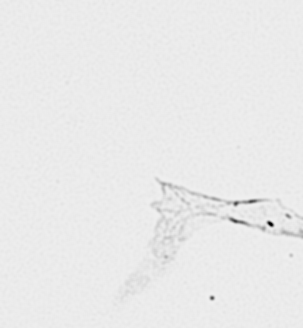

Supplement: Supplementary file 12 — Source data Fig. 3 [file 44318_2025_515_MOESM12_ESM.zip › Figure3/3C/24min.tif]

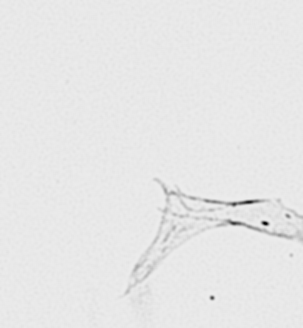

Supplement: Supplementary file 12 — Source data Fig. 3 [file 44318_2025_515_MOESM12_ESM.zip › Figure3/3C/30min.tif]

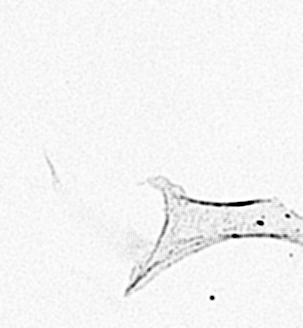

Supplement: Supplementary file 12 — Source data Fig. 3 [file 44318_2025_515_MOESM12_ESM.zip › Figure3/3C/54min.tif]

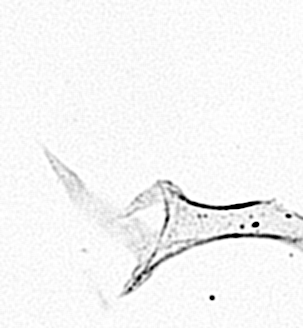

Supplement: Supplementary file 12 — Source data Fig. 3 [file 44318_2025_515_MOESM12_ESM.zip › Figure3/3C/60min.tif]

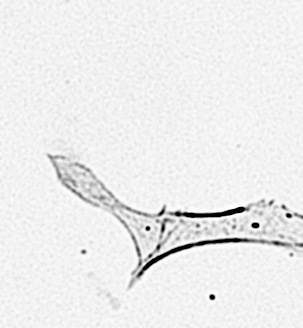

Supplement: Supplementary file 12 — Source data Fig. 3 [file 44318_2025_515_MOESM12_ESM.zip › Figure3/3C/84min.tif]

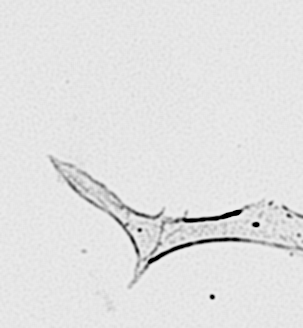

Supplement: Supplementary file 12 — Source data Fig. 3 [file 44318_2025_515_MOESM12_ESM.zip › Figure3/3C/90min.tif]

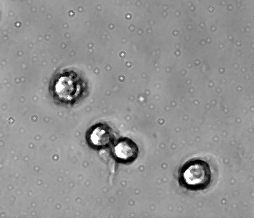

Supplement: Supplementary file 12 — Source data Fig. 3 [file 44318_2025_515_MOESM12_ESM.zip › Figure3/3F/Bleb-0 min.tif]

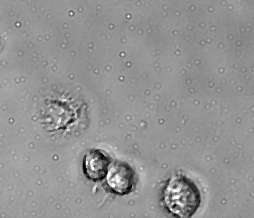

Supplement: Supplementary file 12 — Source data Fig. 3 [file 44318_2025_515_MOESM12_ESM.zip › Figure3/3F/Bleb-360 min.tif]

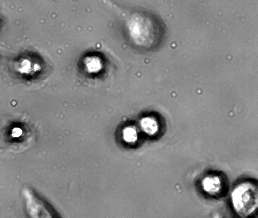

Supplement: Supplementary file 12 — Source data Fig. 3 [file 44318_2025_515_MOESM12_ESM.zip › Figure3/3F/ck666 0 min.tif]

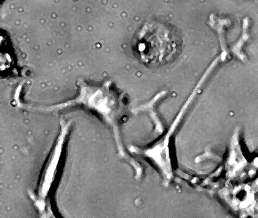

Supplement: Supplementary file 12 — Source data Fig. 3 [file 44318_2025_515_MOESM12_ESM.zip › Figure3/3F/ck666 360 min.tif]

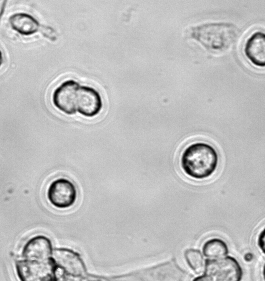

Supplement: Supplementary file 12 — Source data Fig. 3 [file 44318_2025_515_MOESM12_ESM.zip › Figure3/3F/DMSO 0h.tif]

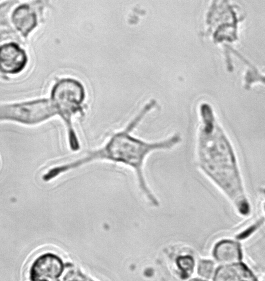

Supplement: Supplementary file 12 — Source data Fig. 3 [file 44318_2025_515_MOESM12_ESM.zip › Figure3/3F/DMSO 6h.tif]

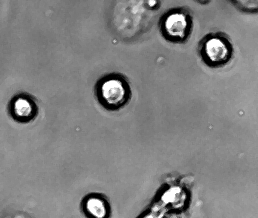

Supplement: Supplementary file 12 — Source data Fig. 3 [file 44318_2025_515_MOESM12_ESM.zip › Figure3/3F/LatB-0 min.tif]

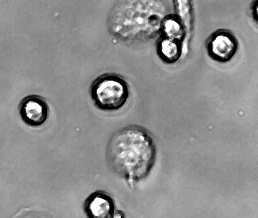

Supplement: Supplementary file 12 — Source data Fig. 3 [file 44318_2025_515_MOESM12_ESM.zip › Figure3/3F/LatB-360 min.tif]

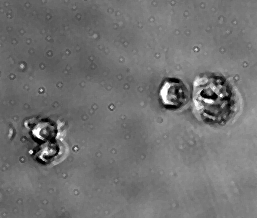

Supplement: Supplementary file 12 — Source data Fig. 3 [file 44318_2025_515_MOESM12_ESM.zip › Figure3/3F/NP-G2-044-0 min.tif]

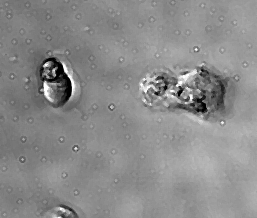

Supplement: Supplementary file 12 — Source data Fig. 3 [file 44318_2025_515_MOESM12_ESM.zip › Figure3/3F/NP-G2-044-360 min.tif]

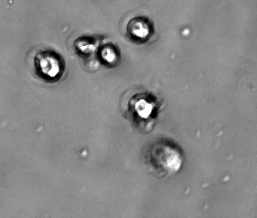

Supplement: Supplementary file 12 — Source data Fig. 3 [file 44318_2025_515_MOESM12_ESM.zip › Figure3/3F/SMIFH2-0 min.tif]

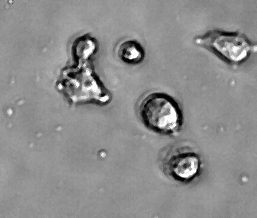

Supplement: Supplementary file 12 — Source data Fig. 3 [file 44318_2025_515_MOESM12_ESM.zip › Figure3/3F/SMIFH2-360 min.tif]

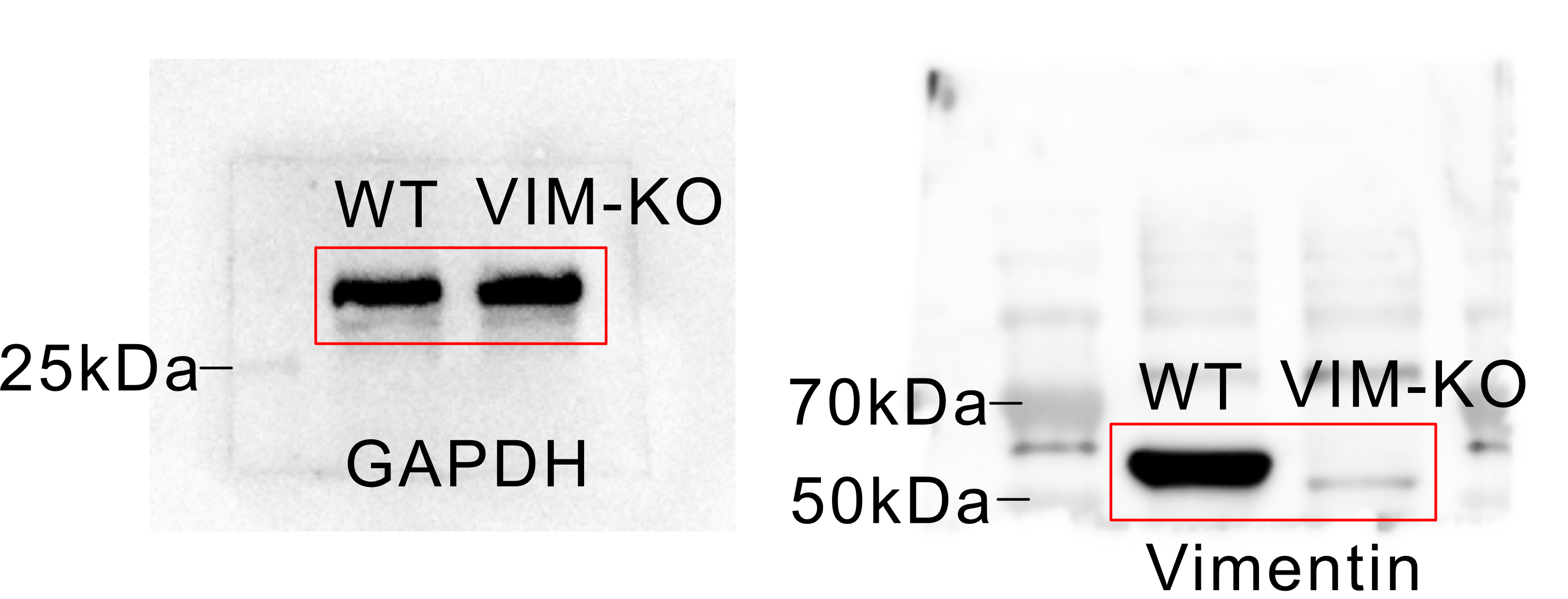

Supplement: Supplementary file 12 — Source data Fig. 3 [file 44318_2025_515_MOESM12_ESM.zip › Figure3/3G/WB cropped.tif]

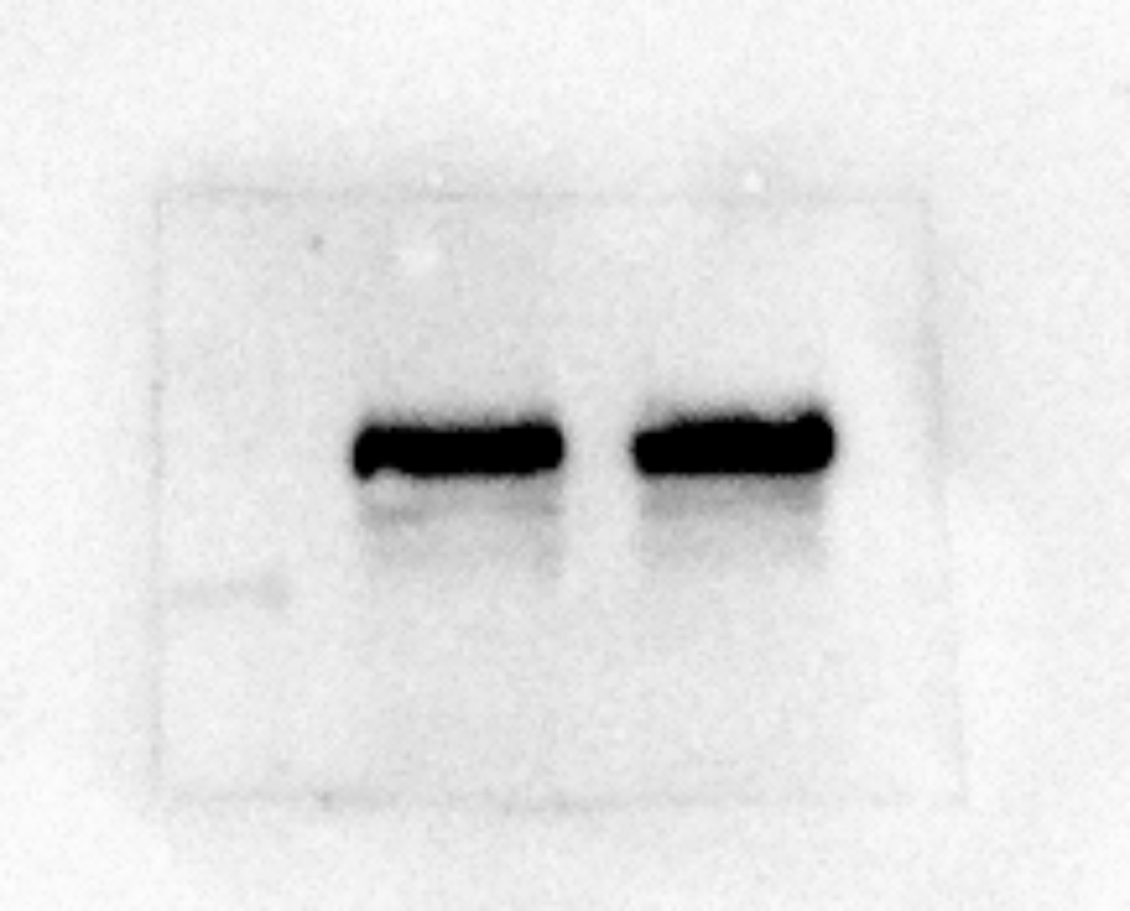

Supplement: Supplementary file 12 — Source data Fig. 3 [file 44318_2025_515_MOESM12_ESM.zip › Figure3/3G/WB GAPDH.tif]

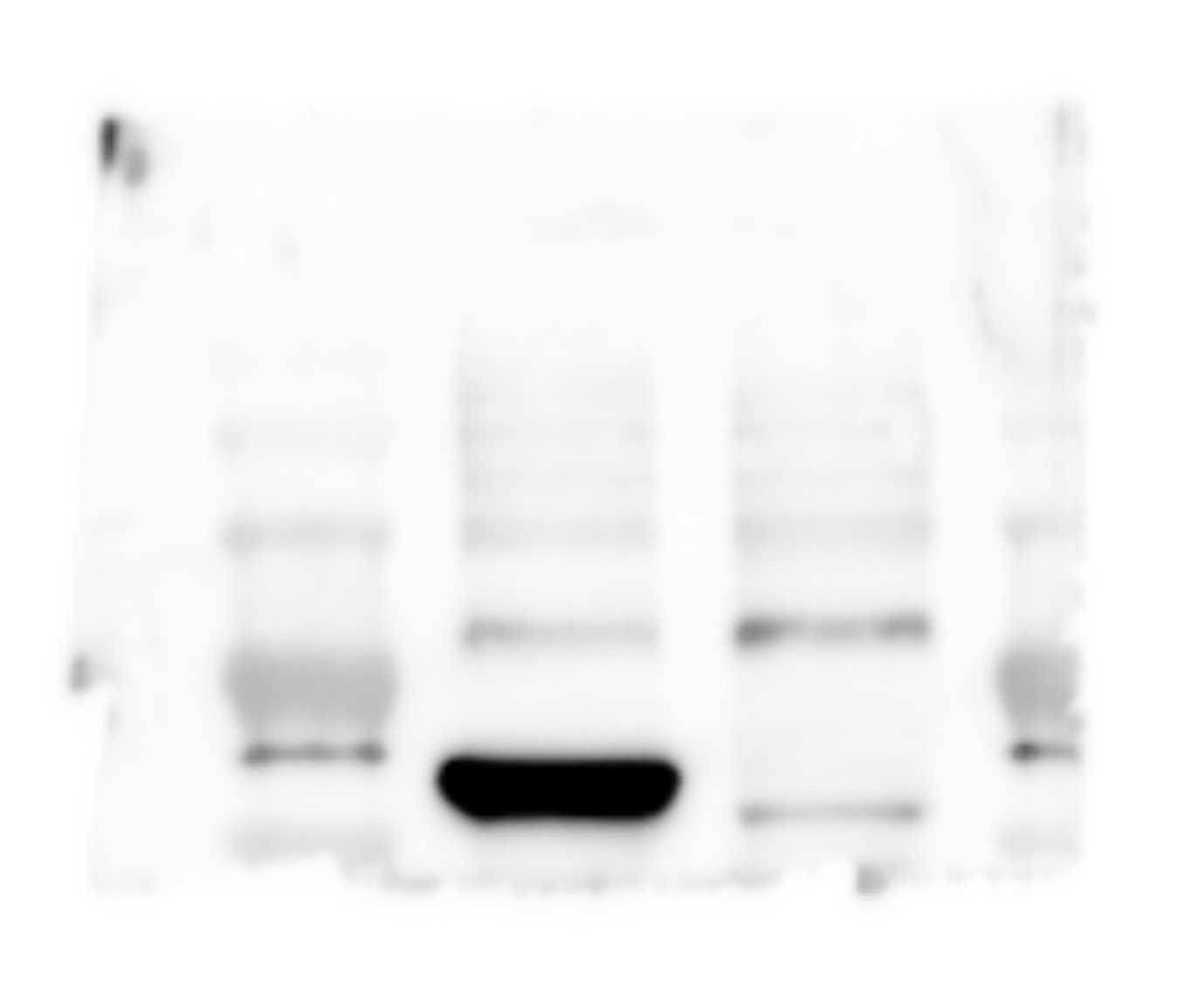

Supplement: Supplementary file 12 — Source data Fig. 3 [file 44318_2025_515_MOESM12_ESM.zip › Figure3/3G/WB VIM.tif]

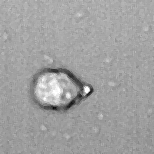

Supplement: Supplementary file 12 — Source data Fig. 3 [file 44318_2025_515_MOESM12_ESM.zip › Figure3/3I/VIM-KO 0min.tif]

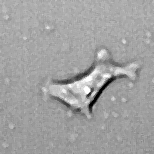

Supplement: Supplementary file 12 — Source data Fig. 3 [file 44318_2025_515_MOESM12_ESM.zip › Figure3/3I/VIM-KO 141min.tif]

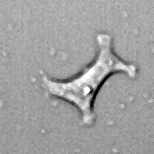

Supplement: Supplementary file 12 — Source data Fig. 3 [file 44318_2025_515_MOESM12_ESM.zip › Figure3/3I/VIM-KO 172min.tif]

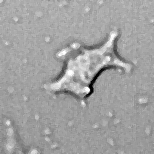

Supplement: Supplementary file 12 — Source data Fig. 3 [file 44318_2025_515_MOESM12_ESM.zip › Figure3/3I/VIM-KO 228min.tif]

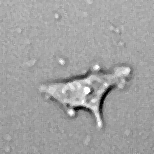

Supplement: Supplementary file 12 — Source data Fig. 3 [file 44318_2025_515_MOESM12_ESM.zip › Figure3/3I/VIM-KO 45min.tif]

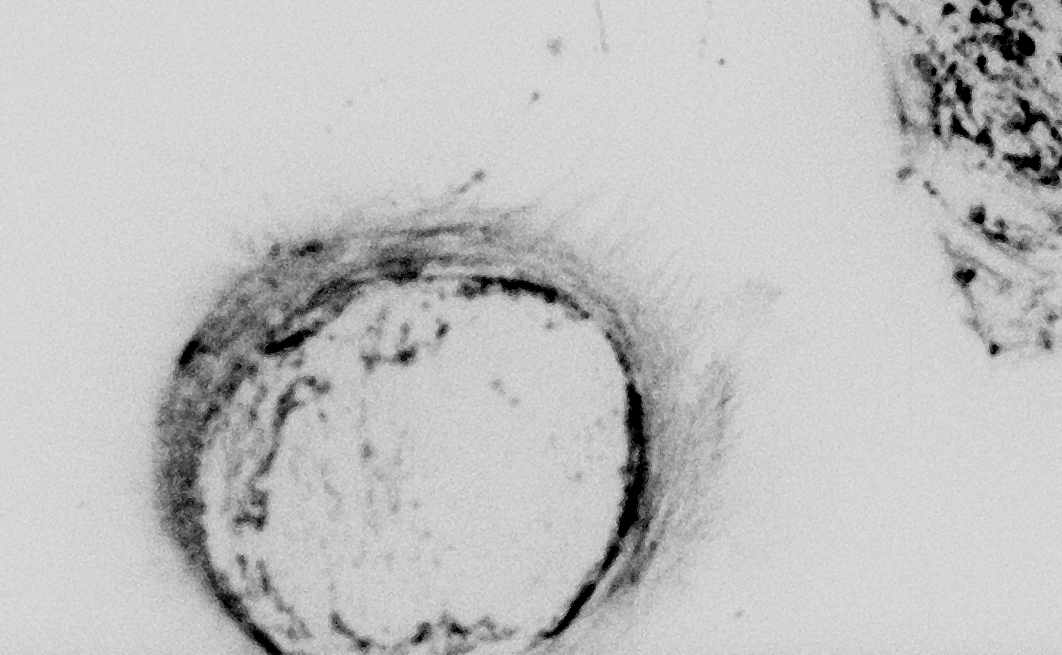

Supplement: Supplementary file 12 — Source data Fig. 3 [file 44318_2025_515_MOESM12_ESM.zip › Figure3/3J/VIM-KO 160min.tif]

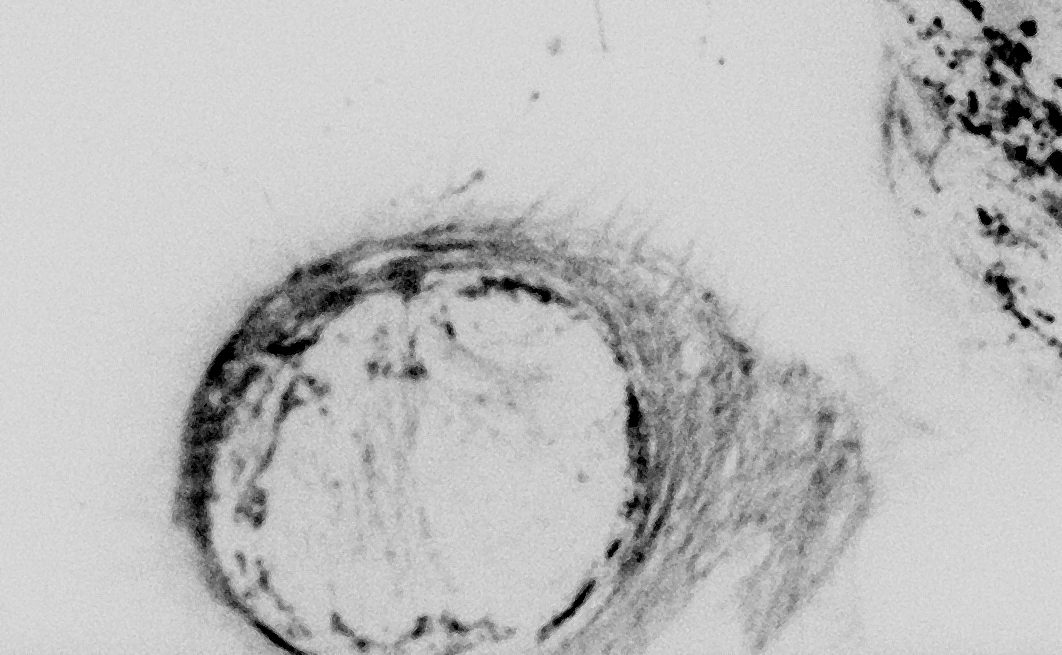

Supplement: Supplementary file 12 — Source data Fig. 3 [file 44318_2025_515_MOESM12_ESM.zip › Figure3/3J/VIM-KO 170min.tif]

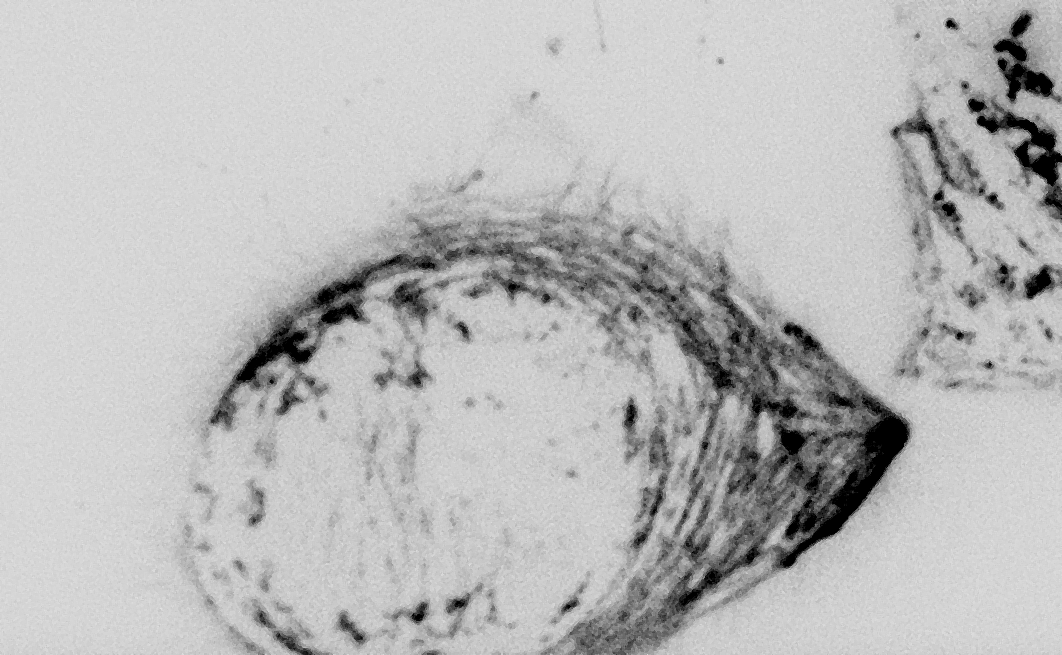

Supplement: Supplementary file 12 — Source data Fig. 3 [file 44318_2025_515_MOESM12_ESM.zip › Figure3/3J/VIM-KO 190min.tif]

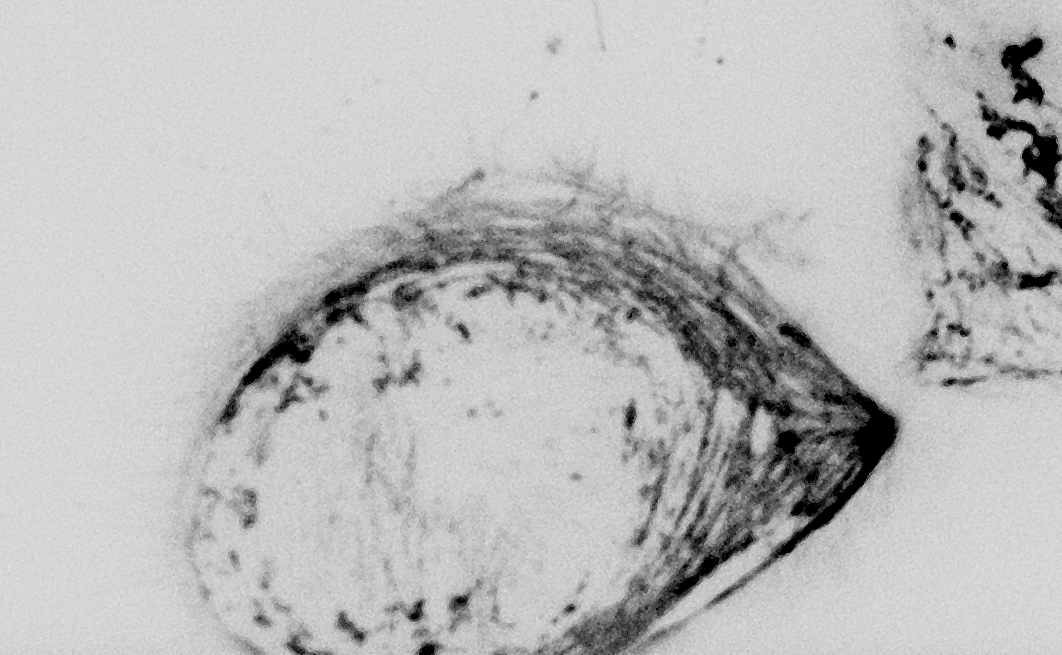

Supplement: Supplementary file 12 — Source data Fig. 3 [file 44318_2025_515_MOESM12_ESM.zip › Figure3/3J/VIM-KO 195min.tif]

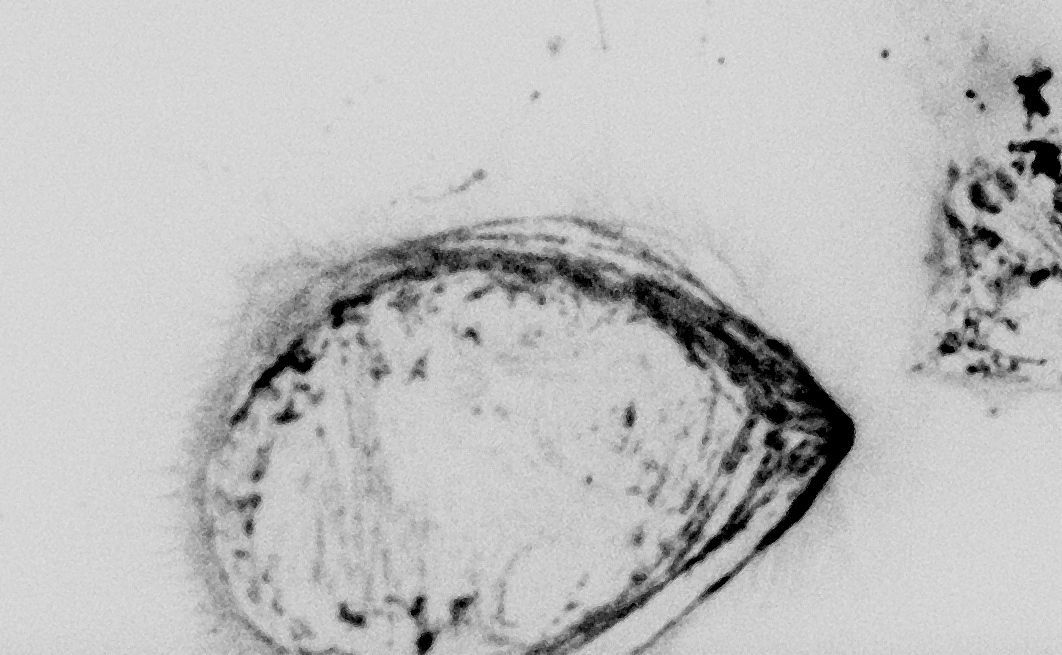

Supplement: Supplementary file 12 — Source data Fig. 3 [file 44318_2025_515_MOESM12_ESM.zip › Figure3/3J/VIM-KO 205min.tif]

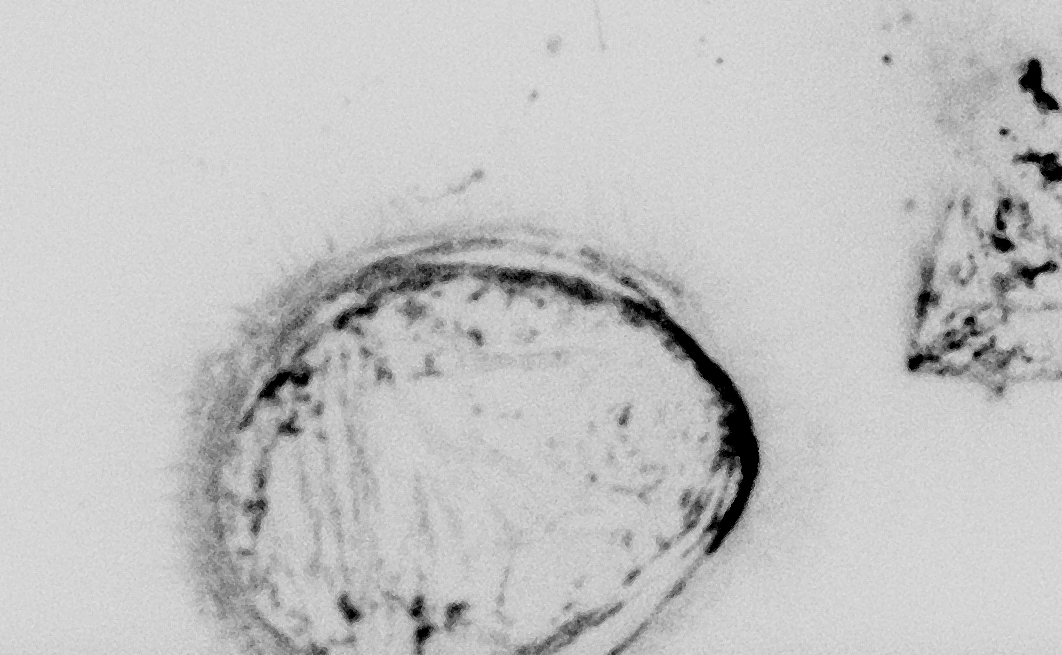

Supplement: Supplementary file 12 — Source data Fig. 3 [file 44318_2025_515_MOESM12_ESM.zip › Figure3/3J/VIM-KO 215min.tif]

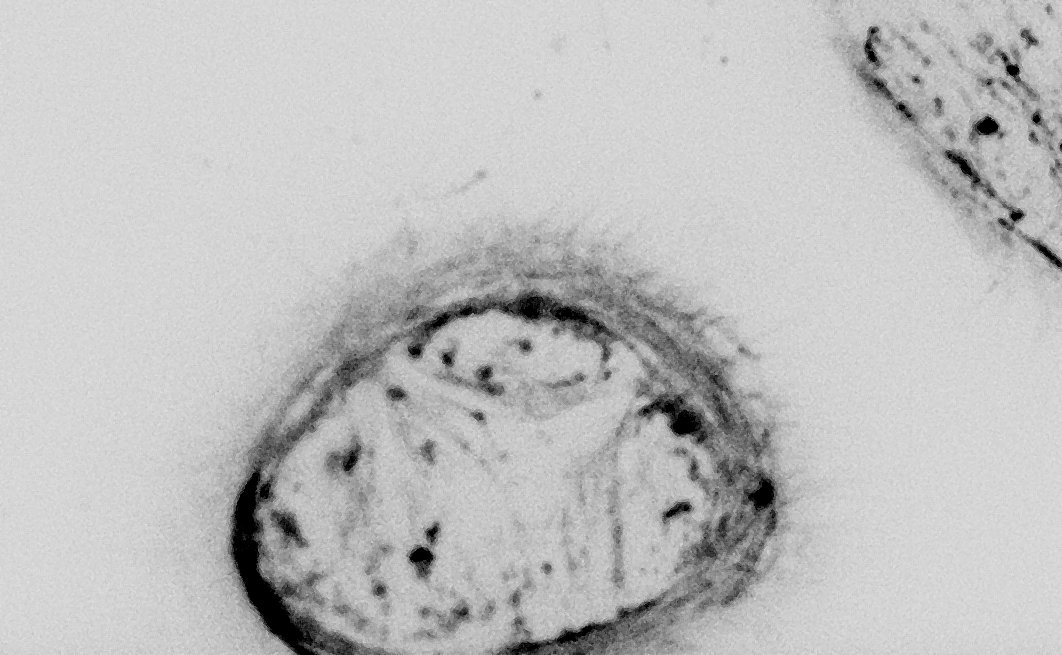

Supplement: Supplementary file 12 — Source data Fig. 3 [file 44318_2025_515_MOESM12_ESM.zip › Figure3/3J/VIM-KO 460min.tif]

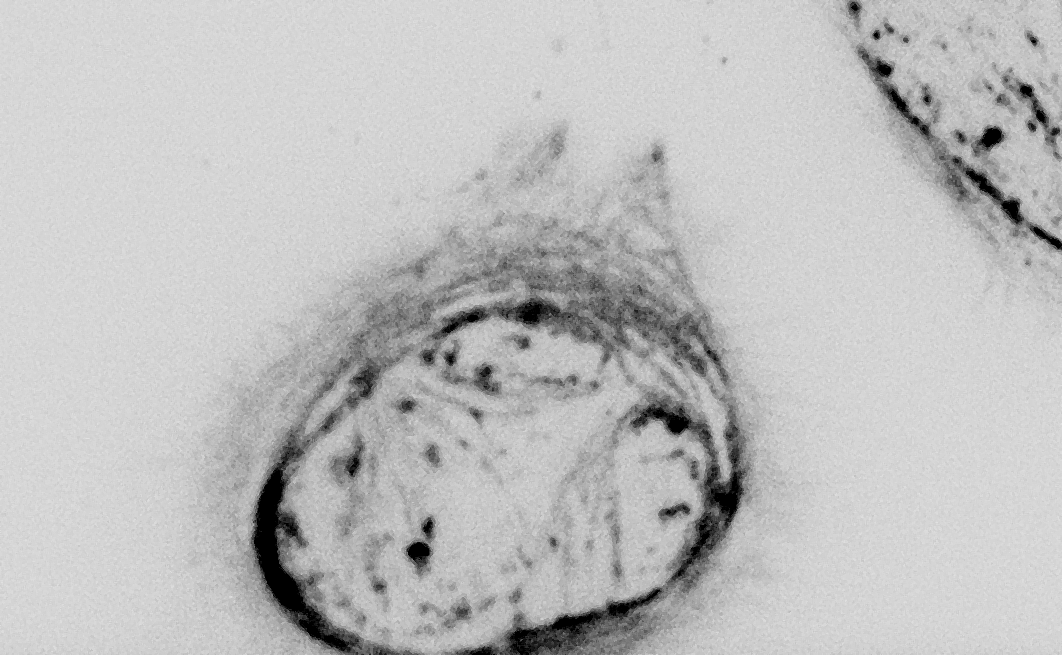

Supplement: Supplementary file 12 — Source data Fig. 3 [file 44318_2025_515_MOESM12_ESM.zip › Figure3/3J/VIM-KO 470min.tif]

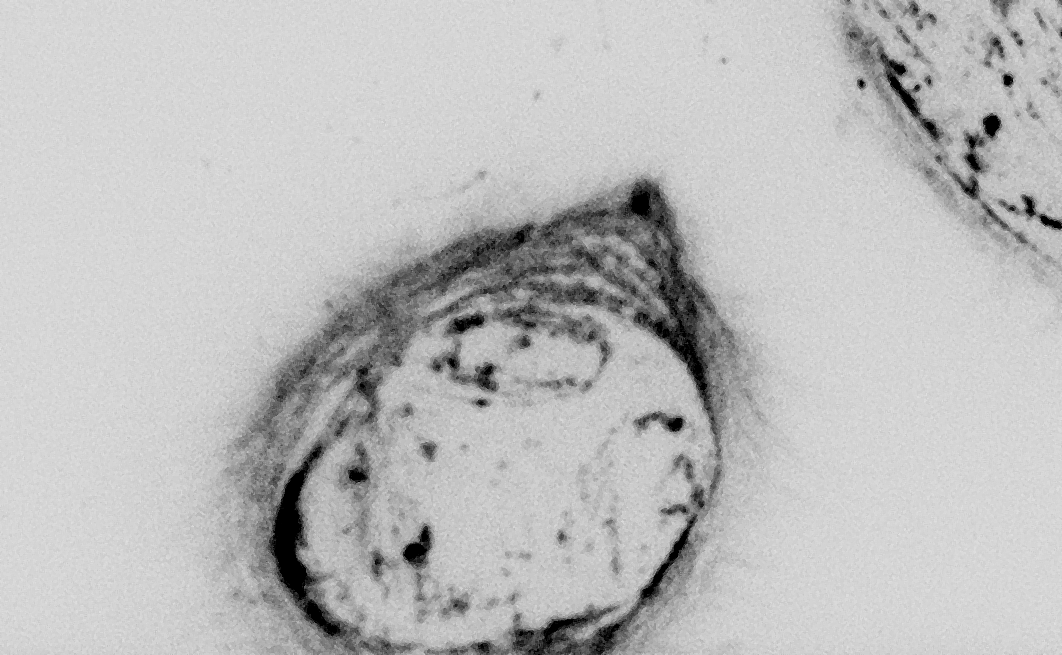

Supplement: Supplementary file 12 — Source data Fig. 3 [file 44318_2025_515_MOESM12_ESM.zip › Figure3/3J/VIM-KO 485min.tif]

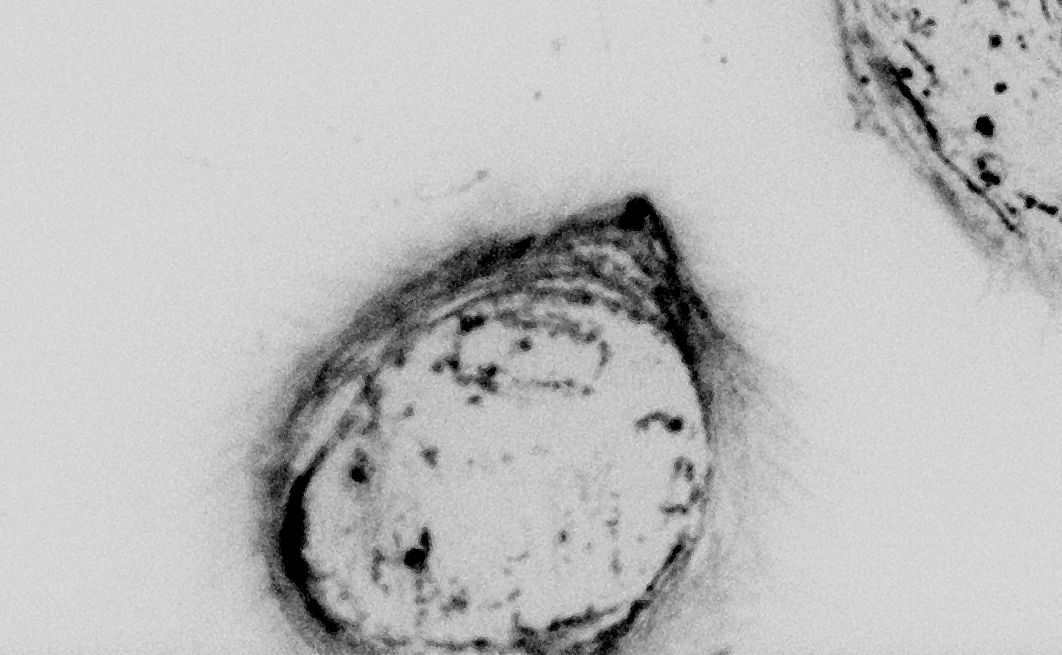

Supplement: Supplementary file 12 — Source data Fig. 3 [file 44318_2025_515_MOESM12_ESM.zip › Figure3/3J/VIM-KO 490min.tif]

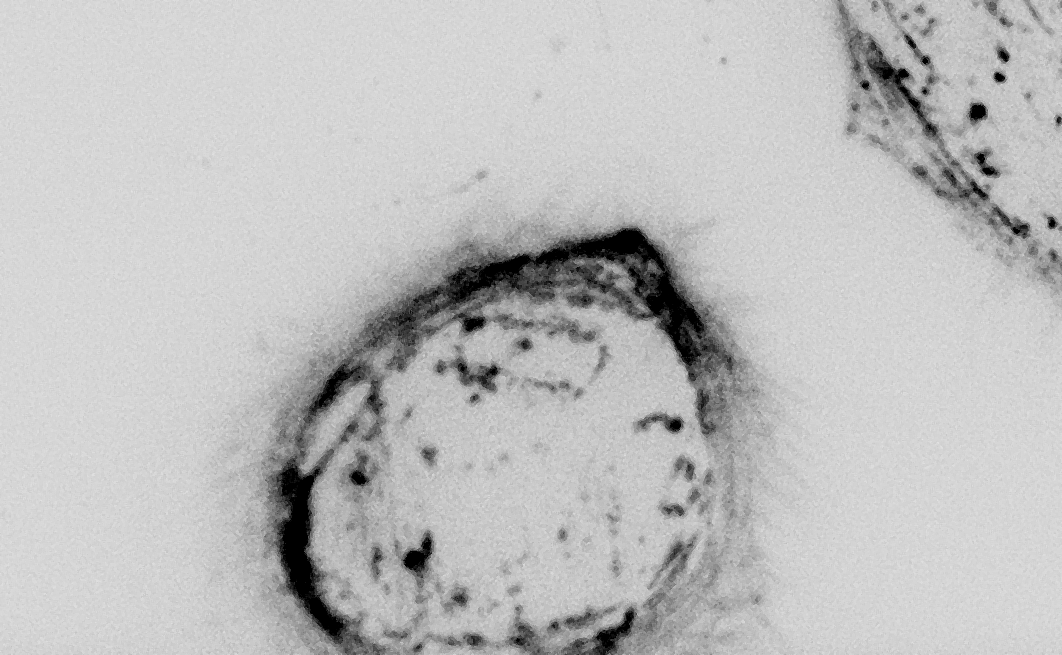

Supplement: Supplementary file 12 — Source data Fig. 3 [file 44318_2025_515_MOESM12_ESM.zip › Figure3/3J/VIM-KO 495min.tif]

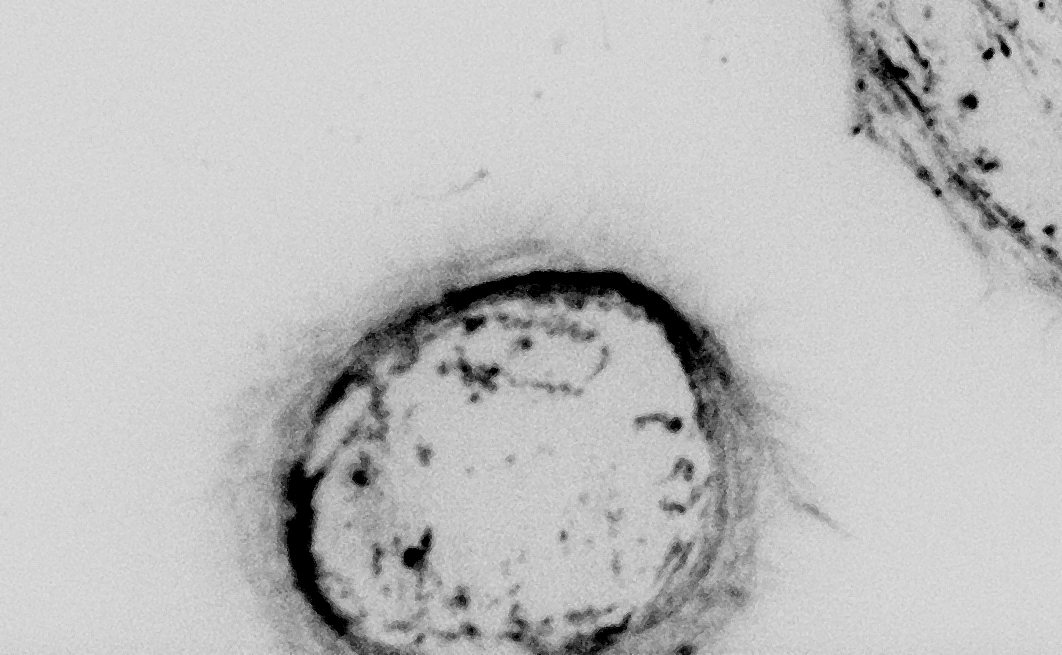

Supplement: Supplementary file 12 — Source data Fig. 3 [file 44318_2025_515_MOESM12_ESM.zip › Figure3/3J/VIM-KO 500min.tif]
