# Supplementary figures and images for: Macrophages form dendrite-like pseudopods to enhance bacterial ingestion (part 2 of 3)
Source: EMBO J. 2025 Jul 28;44(17):4772–802. doi: 10.1038/s44318-025-00515-z (PMC12402336; doi:10.1038/s44318-025-00515-z)

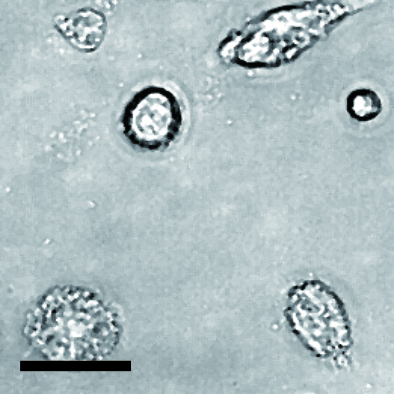

Supplement: Supplementary file 13 — Source data Fig. 4 [file 44318_2025_515_MOESM13_ESM.zip › Figure4/4A/Bacillus.tif]

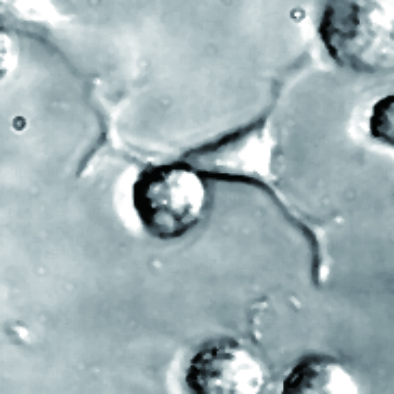

Supplement: Supplementary file 13 — Source data Fig. 4 [file 44318_2025_515_MOESM13_ESM.zip › Figure4/4A/E.coli.tif]

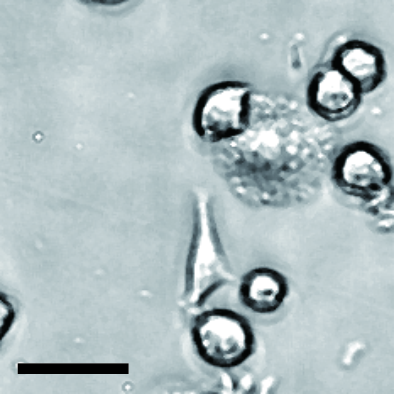

Supplement: Supplementary file 13 — Source data Fig. 4 [file 44318_2025_515_MOESM13_ESM.zip › Figure4/4A/Listeria.tif]

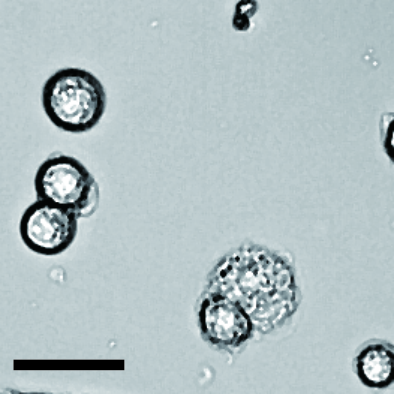

Supplement: Supplementary file 13 — Source data Fig. 4 [file 44318_2025_515_MOESM13_ESM.zip › Figure4/4A/Mock.tif]

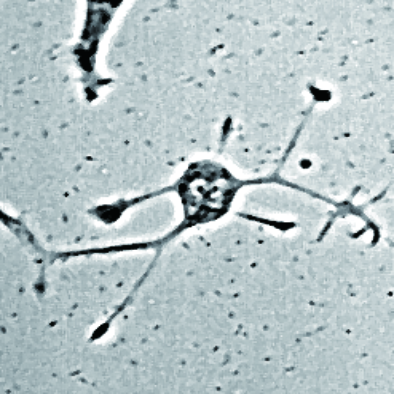

Supplement: Supplementary file 13 — Source data Fig. 4 [file 44318_2025_515_MOESM13_ESM.zip › Figure4/4A/Salmonella.tif]

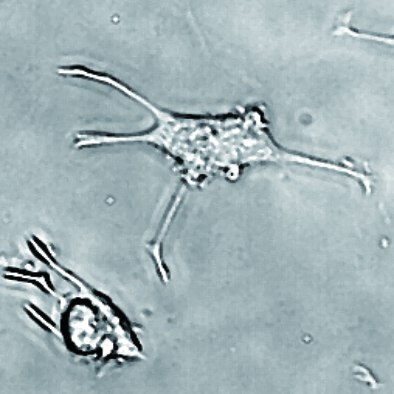

Supplement: Supplementary file 13 — Source data Fig. 4 [file 44318_2025_515_MOESM13_ESM.zip › Figure4/4A/shigella.tif]

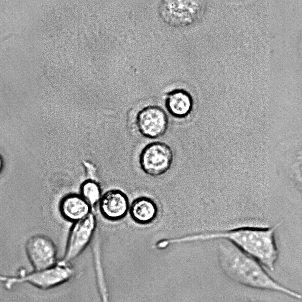

Supplement: Supplementary file 13 — Source data Fig. 4 [file 44318_2025_515_MOESM13_ESM.zip › Figure4/4B/LPS 0min.tif]

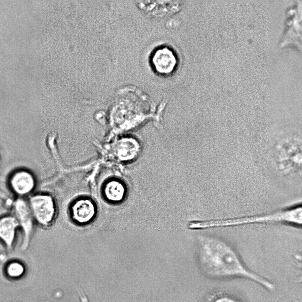

Supplement: Supplementary file 13 — Source data Fig. 4 [file 44318_2025_515_MOESM13_ESM.zip › Figure4/4B/LPS 273min.tif]

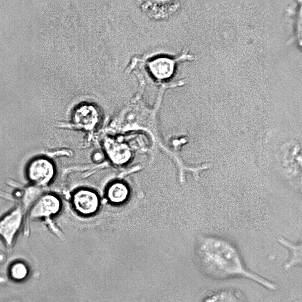

Supplement: Supplementary file 13 — Source data Fig. 4 [file 44318_2025_515_MOESM13_ESM.zip › Figure4/4B/LPS 366min.tif]

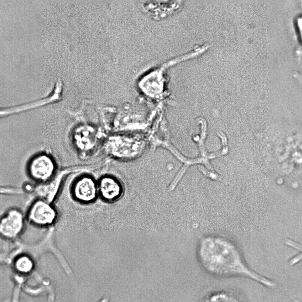

Supplement: Supplementary file 13 — Source data Fig. 4 [file 44318_2025_515_MOESM13_ESM.zip › Figure4/4B/LPS 477min.tif]

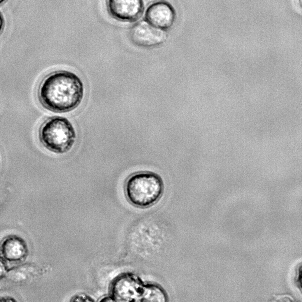

Supplement: Supplementary file 13 — Source data Fig. 4 [file 44318_2025_515_MOESM13_ESM.zip › Figure4/4B/LTA 0min.tif]

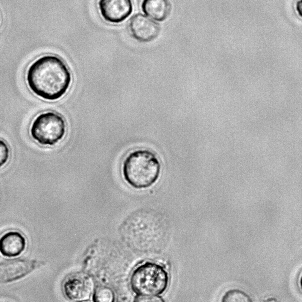

Supplement: Supplementary file 13 — Source data Fig. 4 [file 44318_2025_515_MOESM13_ESM.zip › Figure4/4B/LTA 231min.tif]

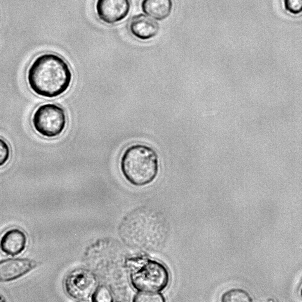

Supplement: Supplementary file 13 — Source data Fig. 4 [file 44318_2025_515_MOESM13_ESM.zip › Figure4/4B/LTA 342min.tif]

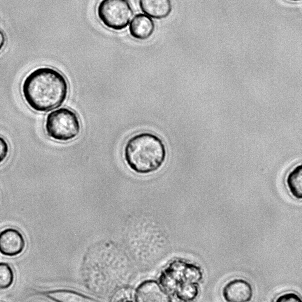

Supplement: Supplementary file 13 — Source data Fig. 4 [file 44318_2025_515_MOESM13_ESM.zip › Figure4/4B/LTA 429min.tif]

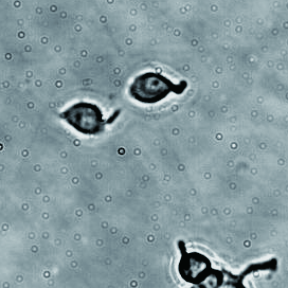

Supplement: Supplementary file 13 — Source data Fig. 4 [file 44318_2025_515_MOESM13_ESM.zip › Figure4/4H/iBMDM LPS 0min.tif]

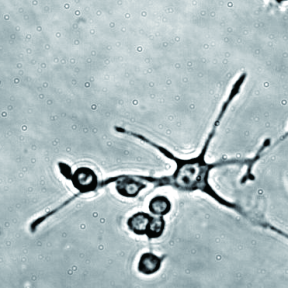

Supplement: Supplementary file 13 — Source data Fig. 4 [file 44318_2025_515_MOESM13_ESM.zip › Figure4/4H/iBMDM LPS 253min.tif]

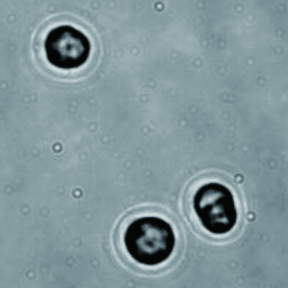

Supplement: Supplementary file 13 — Source data Fig. 4 [file 44318_2025_515_MOESM13_ESM.zip › Figure4/4H/iBMDM Veh 0min.tif]

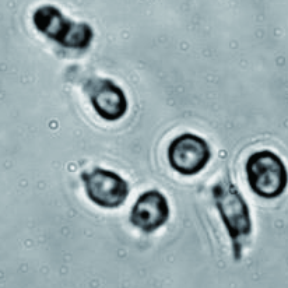

Supplement: Supplementary file 13 — Source data Fig. 4 [file 44318_2025_515_MOESM13_ESM.zip › Figure4/4H/iBMDM Veh 253min.tif]

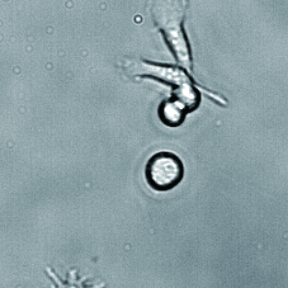

Supplement: Supplementary file 13 — Source data Fig. 4 [file 44318_2025_515_MOESM13_ESM.zip › Figure4/4H/RAW264.7 LPS 0min.tif]

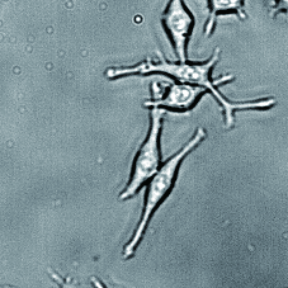

Supplement: Supplementary file 13 — Source data Fig. 4 [file 44318_2025_515_MOESM13_ESM.zip › Figure4/4H/RAW264.7 LPS 432min.tif]

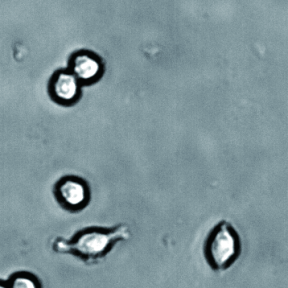

Supplement: Supplementary file 13 — Source data Fig. 4 [file 44318_2025_515_MOESM13_ESM.zip › Figure4/4H/RAW264.7 Veh 0min.tif]

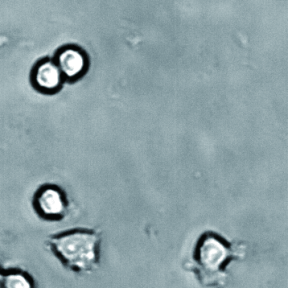

Supplement: Supplementary file 13 — Source data Fig. 4 [file 44318_2025_515_MOESM13_ESM.zip › Figure4/4H/RAW264.7 Veh 432min.tif]

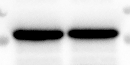

Supplement: Supplementary file 13 — Source data Fig. 4 [file 44318_2025_515_MOESM13_ESM.zip › Figure4/4J/WB GAPDH.tif]

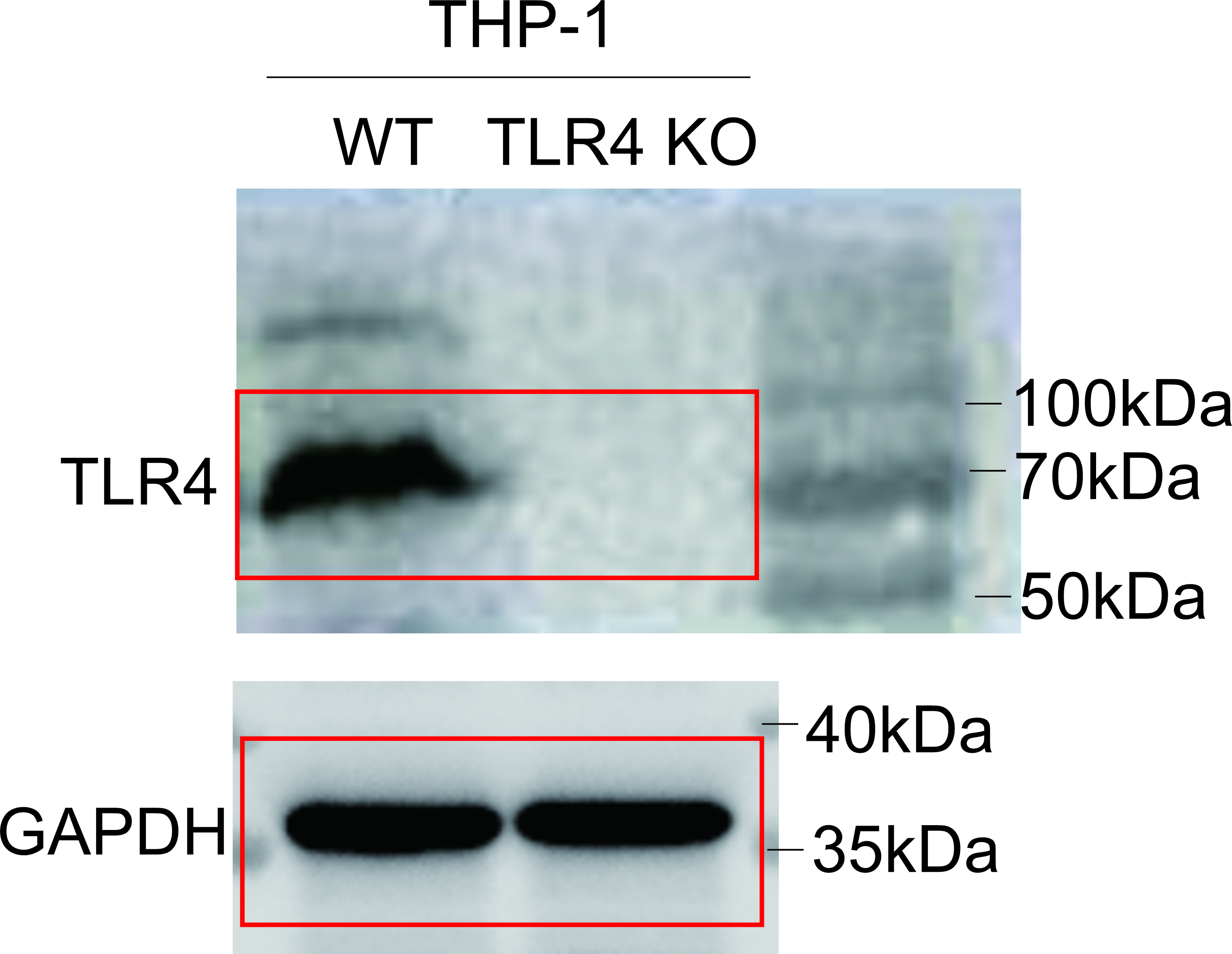

Supplement: Supplementary file 13 — Source data Fig. 4 [file 44318_2025_515_MOESM13_ESM.zip › Figure4/4J/WB source.tif]

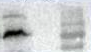

Supplement: Supplementary file 13 — Source data Fig. 4 [file 44318_2025_515_MOESM13_ESM.zip › Figure4/4J/WB TLR4.tif]

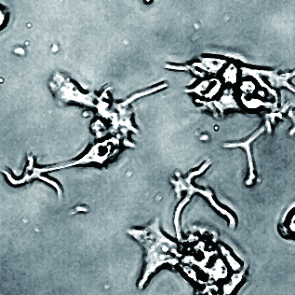

Supplement: Supplementary file 13 — Source data Fig. 4 [file 44318_2025_515_MOESM13_ESM.zip › Figure4/4K/THP-1 LPS.tif]

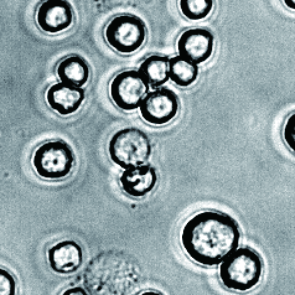

Supplement: Supplementary file 13 — Source data Fig. 4 [file 44318_2025_515_MOESM13_ESM.zip › Figure4/4K/THP-1 TLR4 KO LPS.tif]

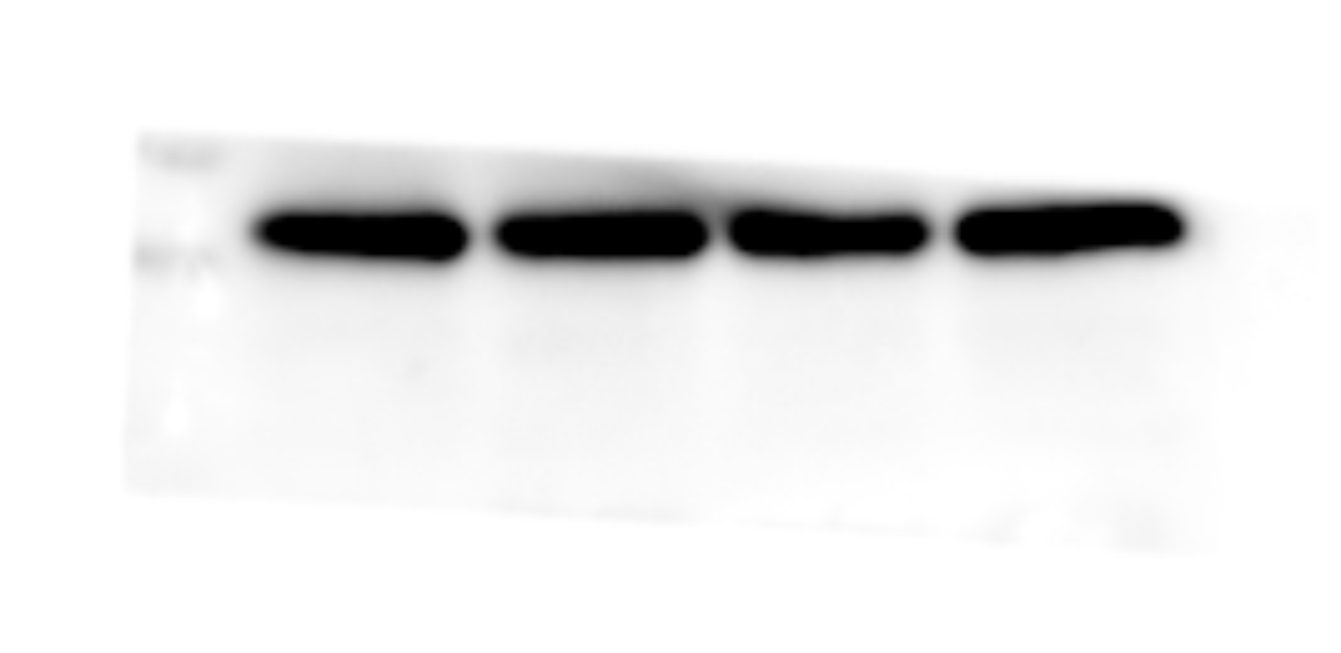

Supplement: Supplementary file 13 — Source data Fig. 4 [file 44318_2025_515_MOESM13_ESM.zip › Figure4/4M/WB GAPDH.tif]

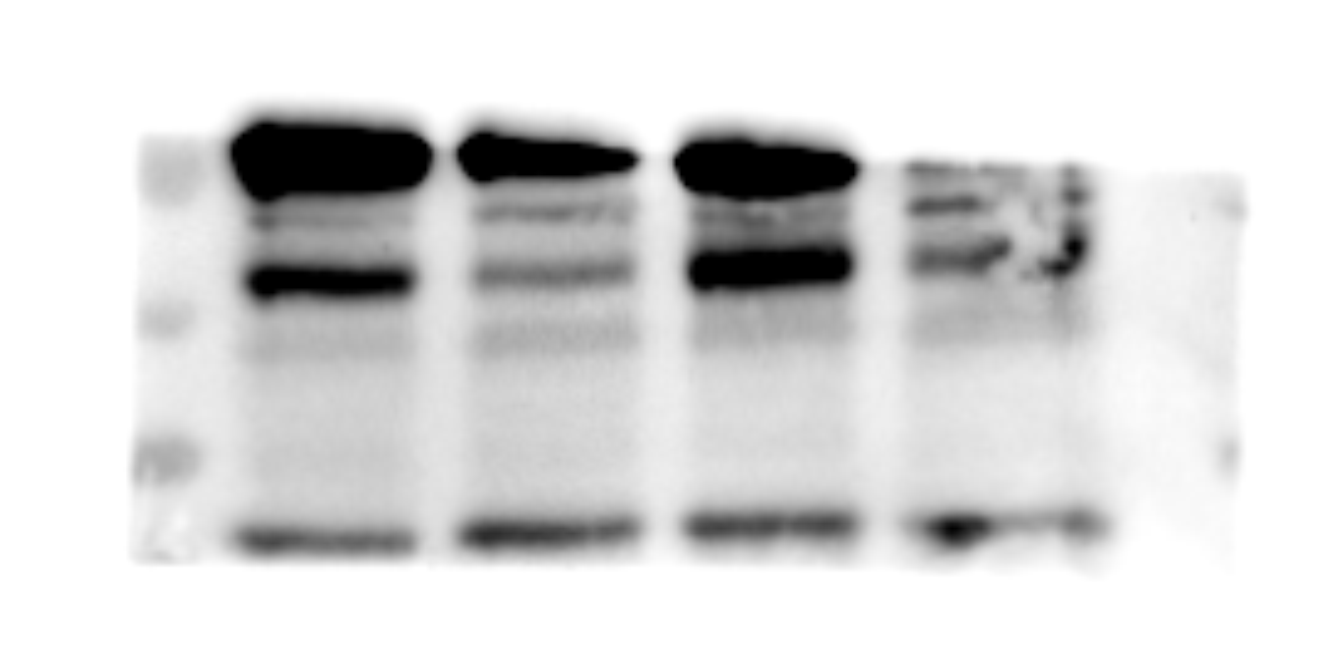

Supplement: Supplementary file 13 — Source data Fig. 4 [file 44318_2025_515_MOESM13_ESM.zip › Figure4/4M/WB P-P65.tif]

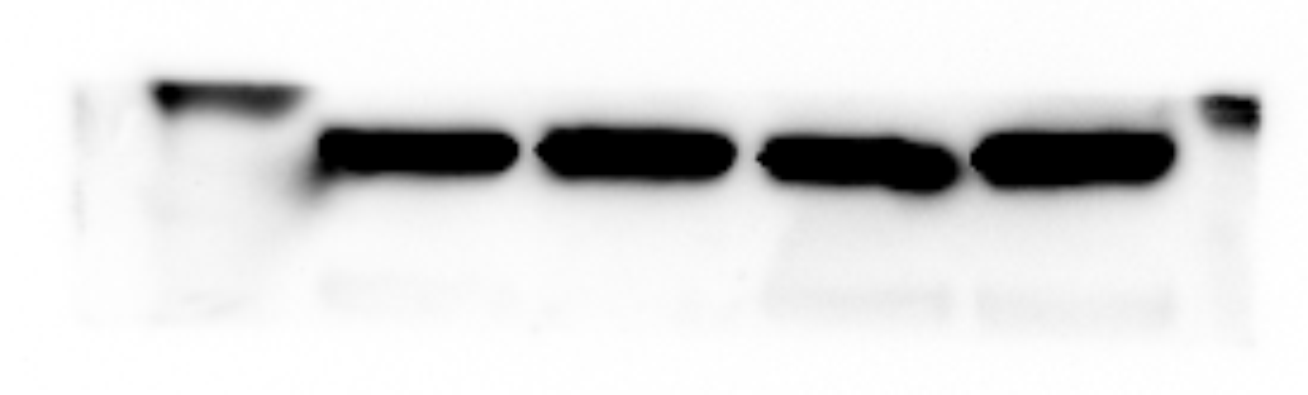

Supplement: Supplementary file 13 — Source data Fig. 4 [file 44318_2025_515_MOESM13_ESM.zip › Figure4/4M/WB P65.tif]

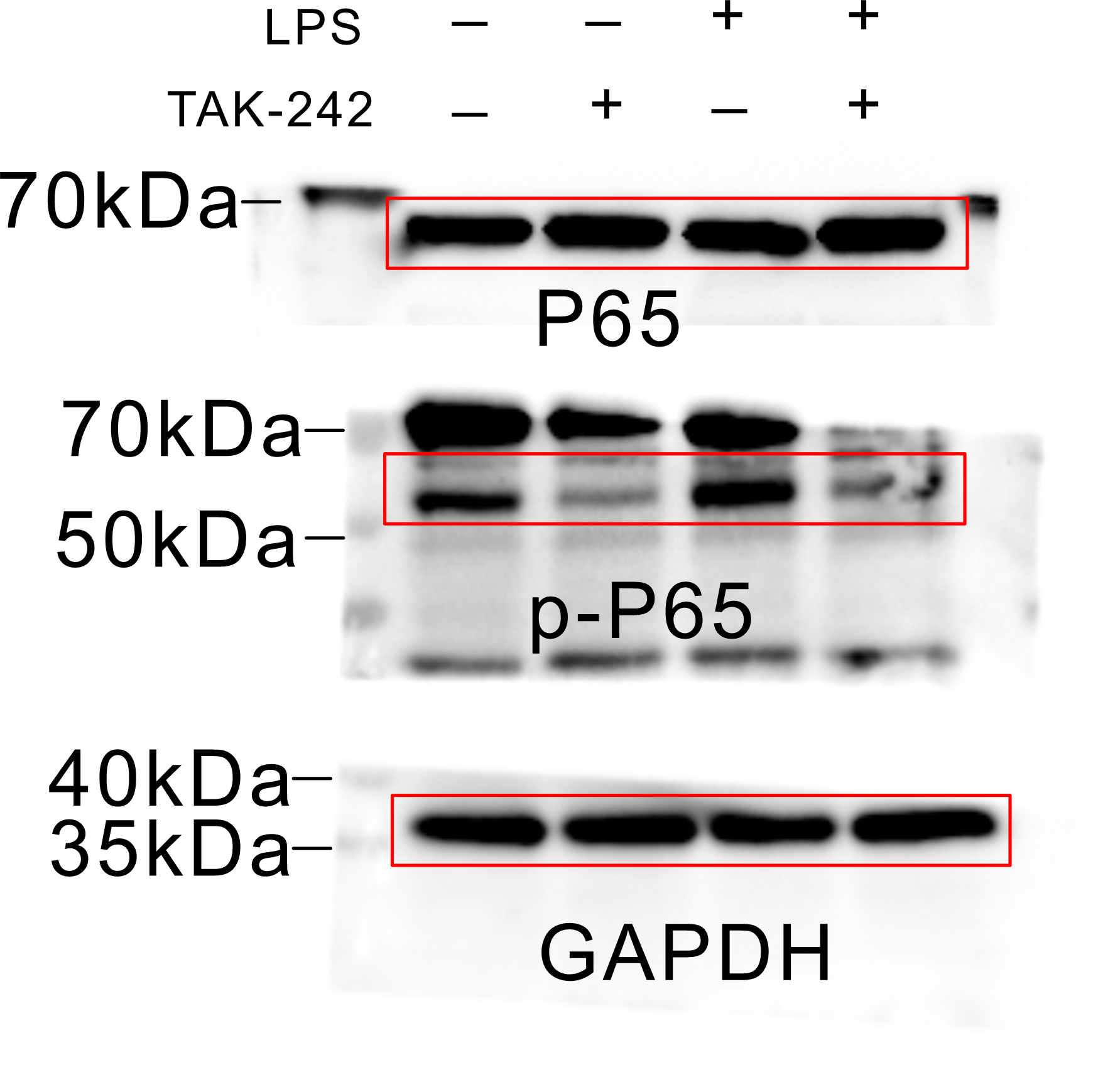

Supplement: Supplementary file 13 — Source data Fig. 4 [file 44318_2025_515_MOESM13_ESM.zip › Figure4/4M/WB source.tif]

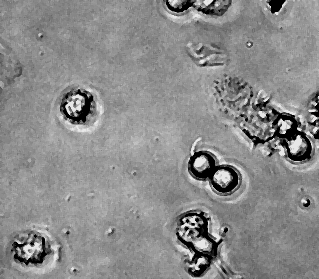

Supplement: Supplementary file 13 — Source data Fig. 4 [file 44318_2025_515_MOESM13_ESM.zip › Figure4/4N/0 min.tif]

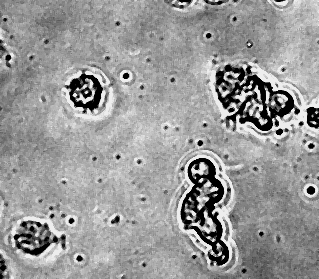

Supplement: Supplementary file 13 — Source data Fig. 4 [file 44318_2025_515_MOESM13_ESM.zip › Figure4/4N/180 min.tif]

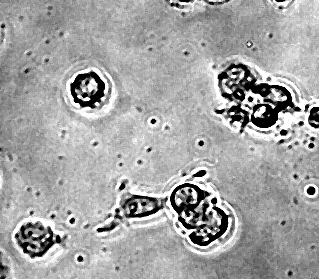

Supplement: Supplementary file 13 — Source data Fig. 4 [file 44318_2025_515_MOESM13_ESM.zip › Figure4/4N/270 min.tif]

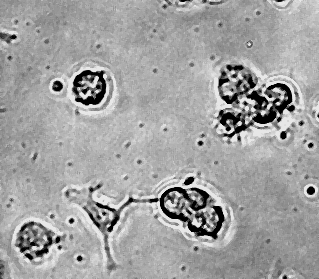

Supplement: Supplementary file 13 — Source data Fig. 4 [file 44318_2025_515_MOESM13_ESM.zip › Figure4/4N/360 min.tif]

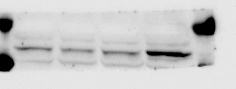

Supplement: Supplementary file 14 — Source data Fig. 5 [file 44318_2025_515_MOESM14_ESM.zip › Figure5/5H/WB ARHGEF3 RAW data.tif]

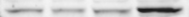

Supplement: Supplementary file 14 — Source data Fig. 5 [file 44318_2025_515_MOESM14_ESM.zip › Figure5/5H/WB ARHGEF3.tif]

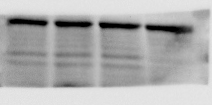

Supplement: Supplementary file 14 — Source data Fig. 5 [file 44318_2025_515_MOESM14_ESM.zip › Figure5/5H/WB GAPDH RAW data.tif]

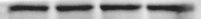

Supplement: Supplementary file 14 — Source data Fig. 5 [file 44318_2025_515_MOESM14_ESM.zip › Figure5/5H/WB GAPDH.tif]

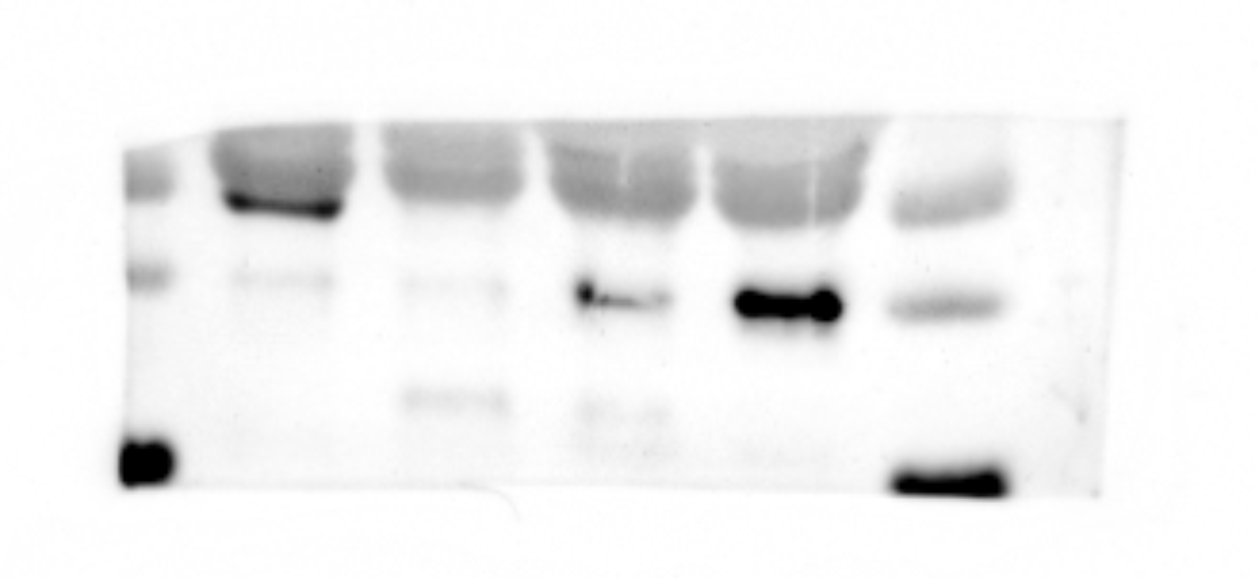

Supplement: Supplementary file 14 — Source data Fig. 5 [file 44318_2025_515_MOESM14_ESM.zip › Figure5/5I/WB Active RhoA.tif]

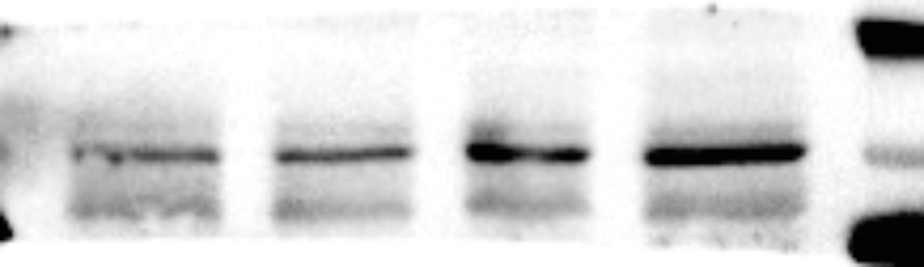

Supplement: Supplementary file 14 — Source data Fig. 5 [file 44318_2025_515_MOESM14_ESM.zip › Figure5/5I/WB ARHGEF3.tif]

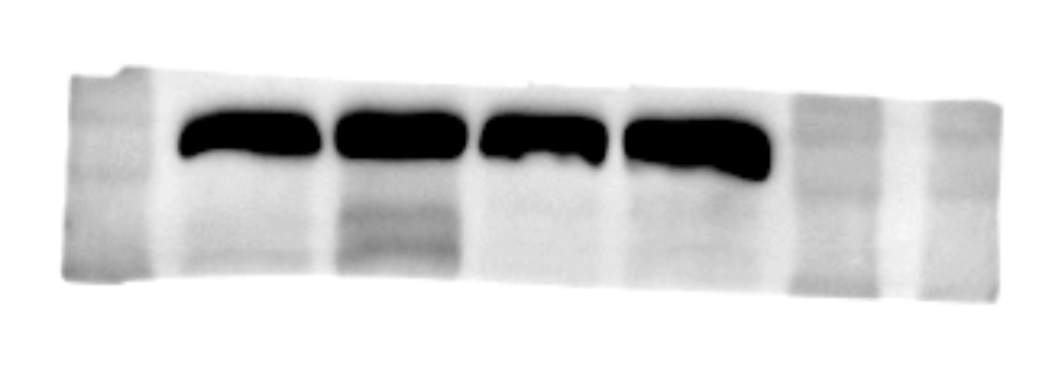

Supplement: Supplementary file 14 — Source data Fig. 5 [file 44318_2025_515_MOESM14_ESM.zip › Figure5/5I/WB GAPDH.tif]

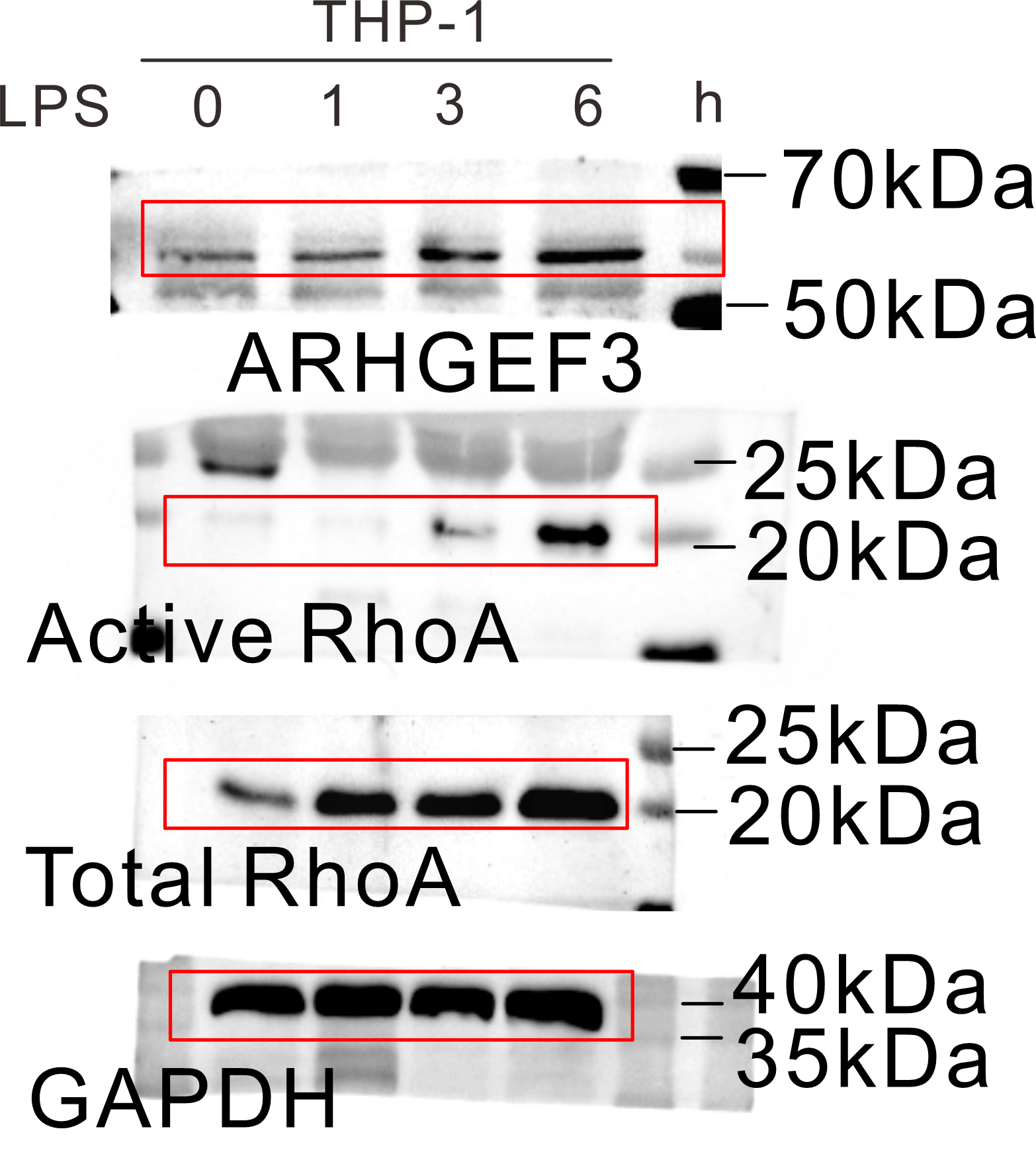

Supplement: Supplementary file 14 — Source data Fig. 5 [file 44318_2025_515_MOESM14_ESM.zip › Figure5/5I/WB source.tif]

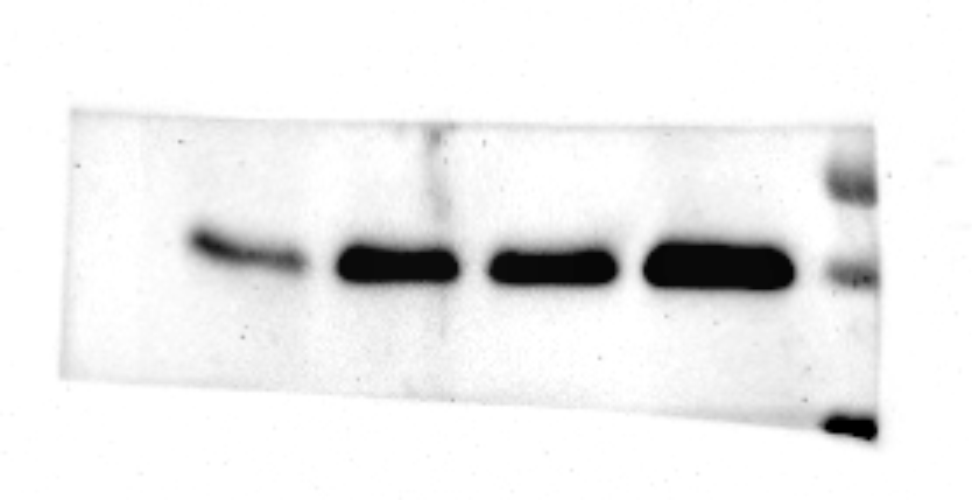

Supplement: Supplementary file 14 — Source data Fig. 5 [file 44318_2025_515_MOESM14_ESM.zip › Figure5/5I/WB Total RhoA.tif]

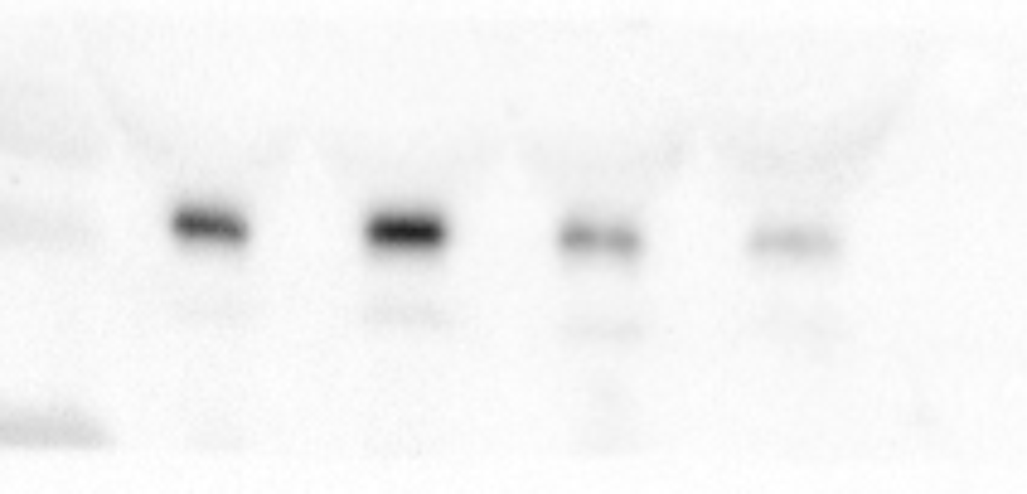

Supplement: Supplementary file 14 — Source data Fig. 5 [file 44318_2025_515_MOESM14_ESM.zip › Figure5/5K/WB active RhoA.tif]

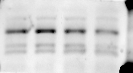

Supplement: Supplementary file 14 — Source data Fig. 5 [file 44318_2025_515_MOESM14_ESM.zip › Figure5/5K/WB ARHGEF3.tif]

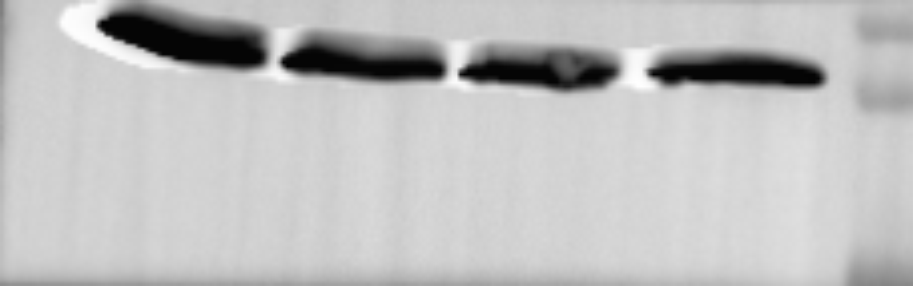

Supplement: Supplementary file 14 — Source data Fig. 5 [file 44318_2025_515_MOESM14_ESM.zip › Figure5/5K/WB GAPDH.tif]

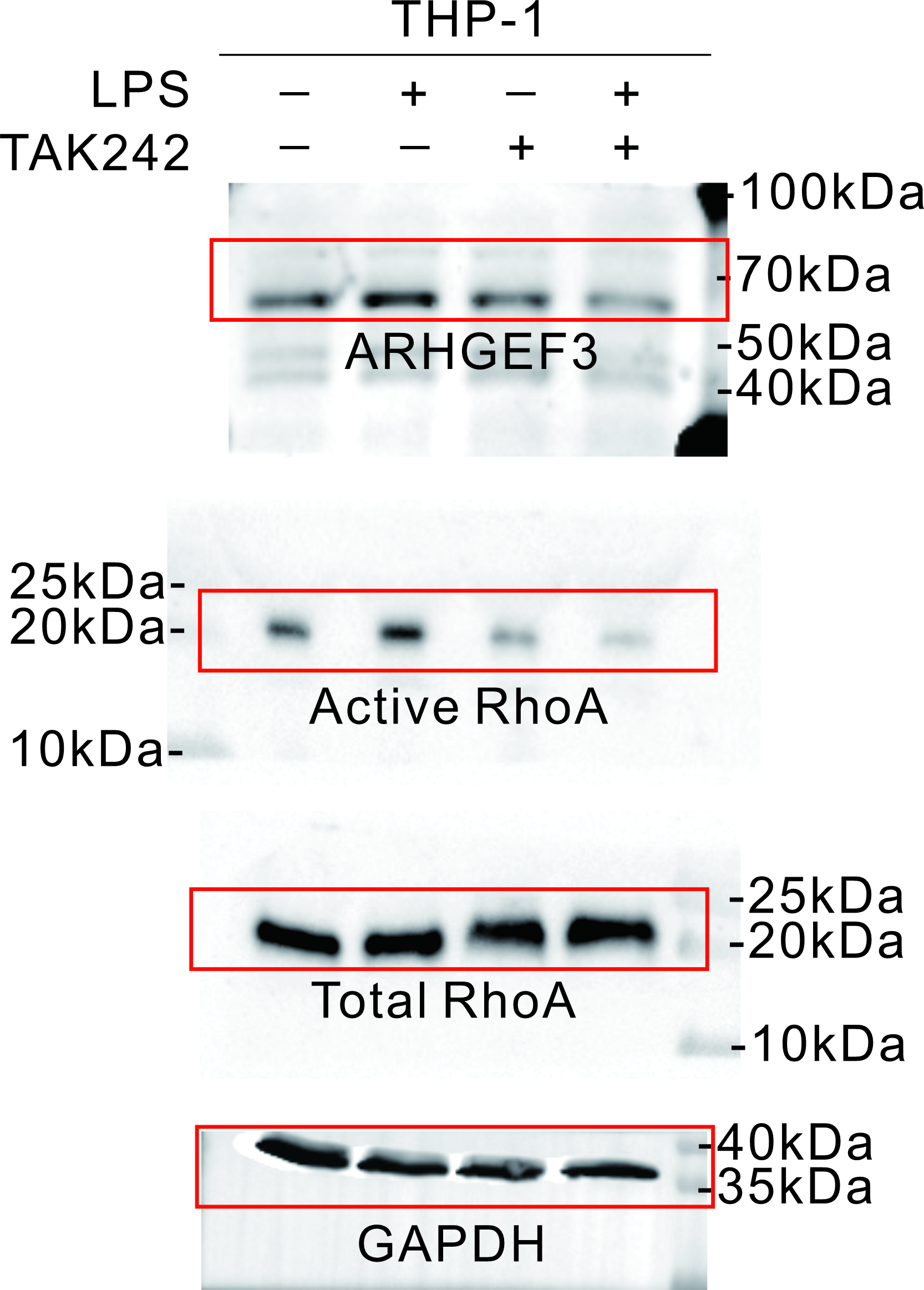

Supplement: Supplementary file 14 — Source data Fig. 5 [file 44318_2025_515_MOESM14_ESM.zip › Figure5/5K/WB source.tif]

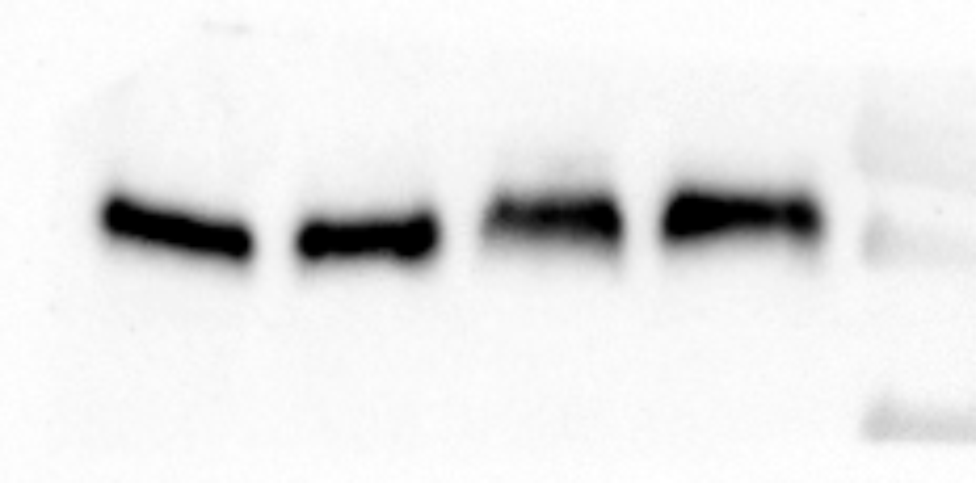

Supplement: Supplementary file 14 — Source data Fig. 5 [file 44318_2025_515_MOESM14_ESM.zip › Figure5/5K/WB total RhoA.tif]

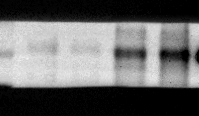

Supplement: Supplementary file 14 — Source data Fig. 5 [file 44318_2025_515_MOESM14_ESM.zip › Figure5/5P/IP ARHGEF3.tif]

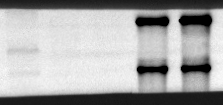

Supplement: Supplementary file 14 — Source data Fig. 5 [file 44318_2025_515_MOESM14_ESM.zip › Figure5/5P/IP TLR4.tif]

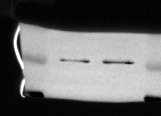

Supplement: Supplementary file 14 — Source data Fig. 5 [file 44318_2025_515_MOESM14_ESM.zip › Figure5/5P/TCL ARHGEF3.tif]

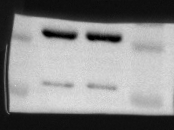

Supplement: Supplementary file 14 — Source data Fig. 5 [file 44318_2025_515_MOESM14_ESM.zip › Figure5/5P/TCL GAPDH.tif]

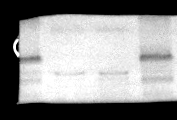

Supplement: Supplementary file 14 — Source data Fig. 5 [file 44318_2025_515_MOESM14_ESM.zip › Figure5/5P/TCL TLR4.tif]

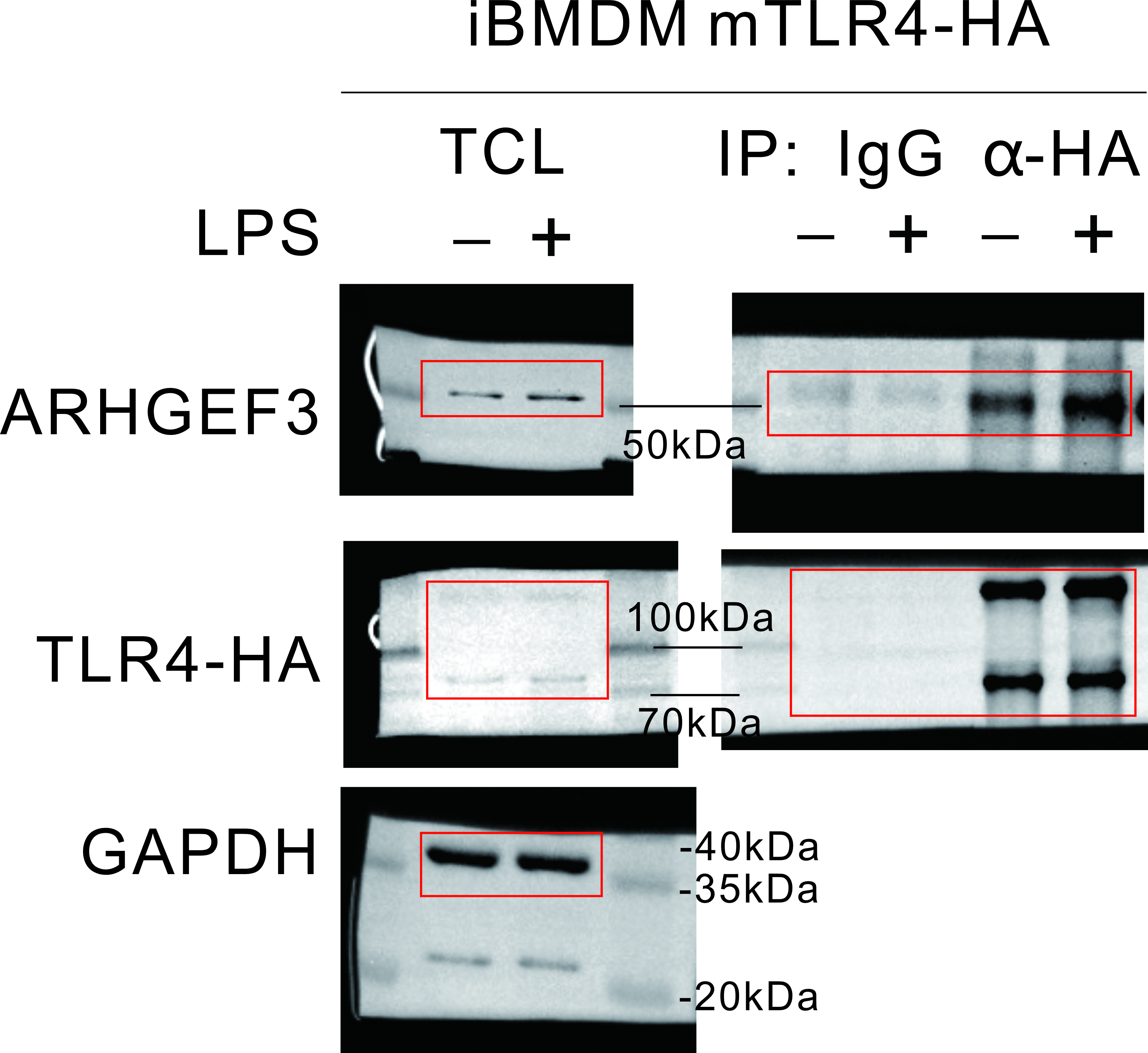

Supplement: Supplementary file 14 — Source data Fig. 5 [file 44318_2025_515_MOESM14_ESM.zip › Figure5/5P/WB source.tif]

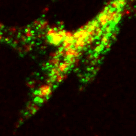

Supplement: Supplementary file 14 — Source data Fig. 5 [file 44318_2025_515_MOESM14_ESM.zip › Figure5/5Q/THP-1 croped LPA merge.tif (RGB).tif]

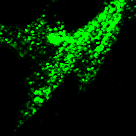

Supplement: Supplementary file 14 — Source data Fig. 5 [file 44318_2025_515_MOESM14_ESM.zip › Figure5/5Q/THP-1 croped LPS merge.tif (RGB) GEF3.tif]

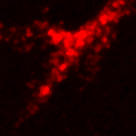

Supplement: Supplementary file 14 — Source data Fig. 5 [file 44318_2025_515_MOESM14_ESM.zip › Figure5/5Q/THP-1 croped LPS merge.tif (RGB) TLR4.tif]

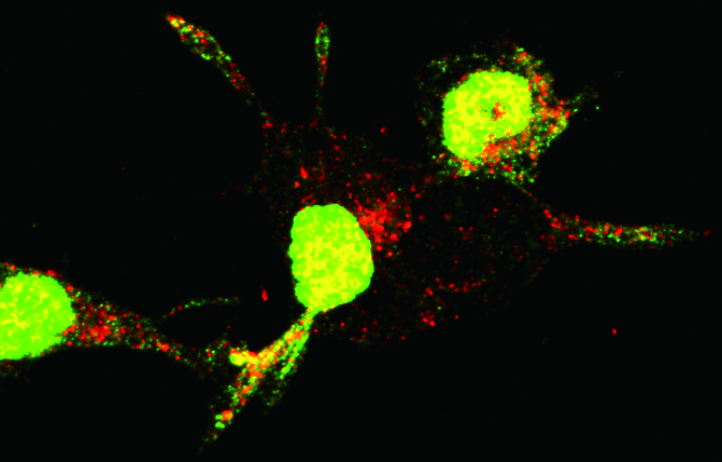

Supplement: Supplementary file 14 — Source data Fig. 5 [file 44318_2025_515_MOESM14_ESM.zip › Figure5/5Q/THP-1 LPS merge.tif]

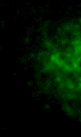

Supplement: Supplementary file 14 — Source data Fig. 5 [file 44318_2025_515_MOESM14_ESM.zip › Figure5/5Q/THP-1 TAK242 cropped GEF3.tif]

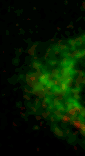

Supplement: Supplementary file 14 — Source data Fig. 5 [file 44318_2025_515_MOESM14_ESM.zip › Figure5/5Q/THP-1 TAK242 cropped merge.tif]

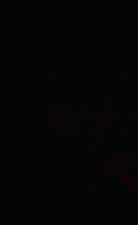

Supplement: Supplementary file 14 — Source data Fig. 5 [file 44318_2025_515_MOESM14_ESM.zip › Figure5/5Q/THP-1 TAK242 cropped TLR4.tif]

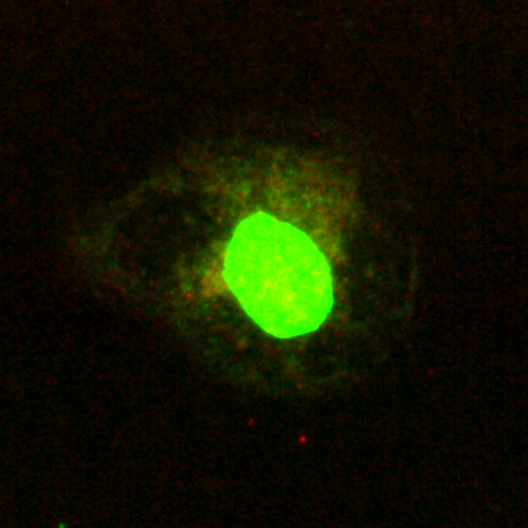

Supplement: Supplementary file 14 — Source data Fig. 5 [file 44318_2025_515_MOESM14_ESM.zip › Figure5/5Q/THP-1 TAK242 merge.tif]

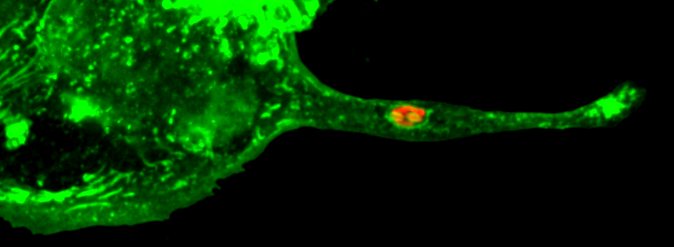

Supplement: Supplementary file 15 — Source data Fig. 6 [file 44318_2025_515_MOESM15_ESM.zip › Figure6/6A/S.ty uptake.tif]

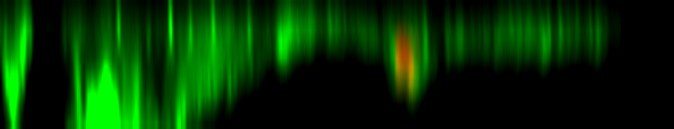

Supplement: Supplementary file 15 — Source data Fig. 6 [file 44318_2025_515_MOESM15_ESM.zip › Figure6/6A/XZ.tif]

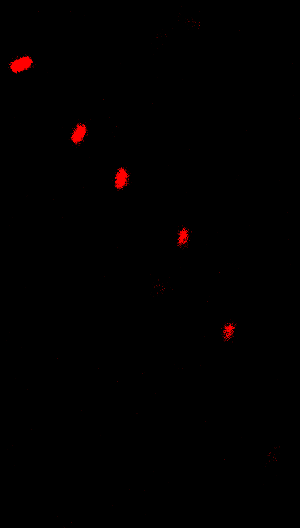

Supplement: Supplementary file 15 — Source data Fig. 6 [file 44318_2025_515_MOESM15_ESM.zip › Figure6/6B/Montage.tif]

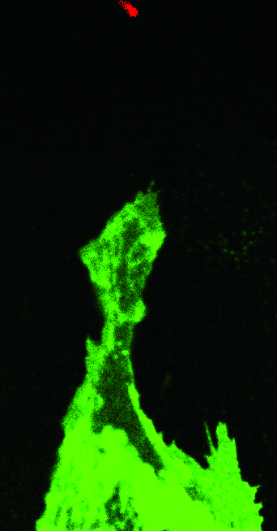

Supplement: Supplementary file 15 — Source data Fig. 6 [file 44318_2025_515_MOESM15_ESM.zip › Figure6/6B/Uptake 0s.tif]

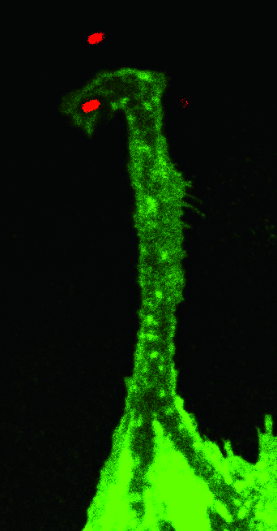

Supplement: Supplementary file 15 — Source data Fig. 6 [file 44318_2025_515_MOESM15_ESM.zip › Figure6/6B/Uptake 1350s.tif]

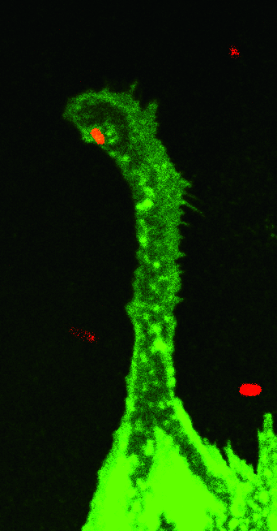

Supplement: Supplementary file 15 — Source data Fig. 6 [file 44318_2025_515_MOESM15_ESM.zip › Figure6/6B/Uptake 1980s.tif]

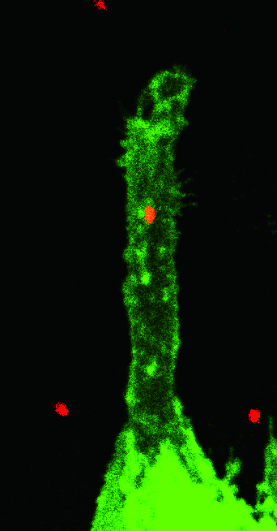

Supplement: Supplementary file 15 — Source data Fig. 6 [file 44318_2025_515_MOESM15_ESM.zip › Figure6/6B/Uptake 2610s.tif]

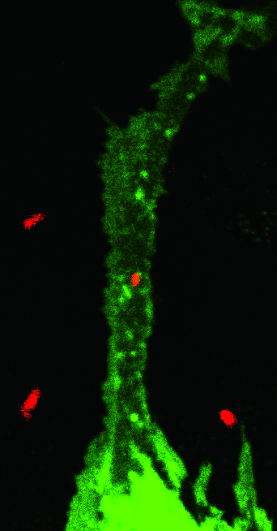

Supplement: Supplementary file 15 — Source data Fig. 6 [file 44318_2025_515_MOESM15_ESM.zip › Figure6/6B/Uptake 3210s.tif]

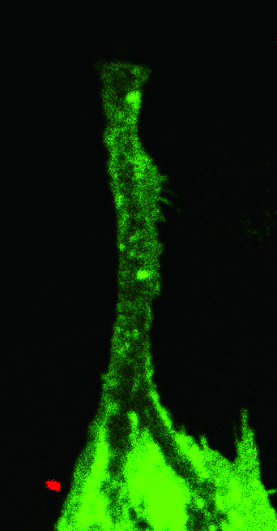

Supplement: Supplementary file 15 — Source data Fig. 6 [file 44318_2025_515_MOESM15_ESM.zip › Figure6/6B/Uptake 630s.tif]

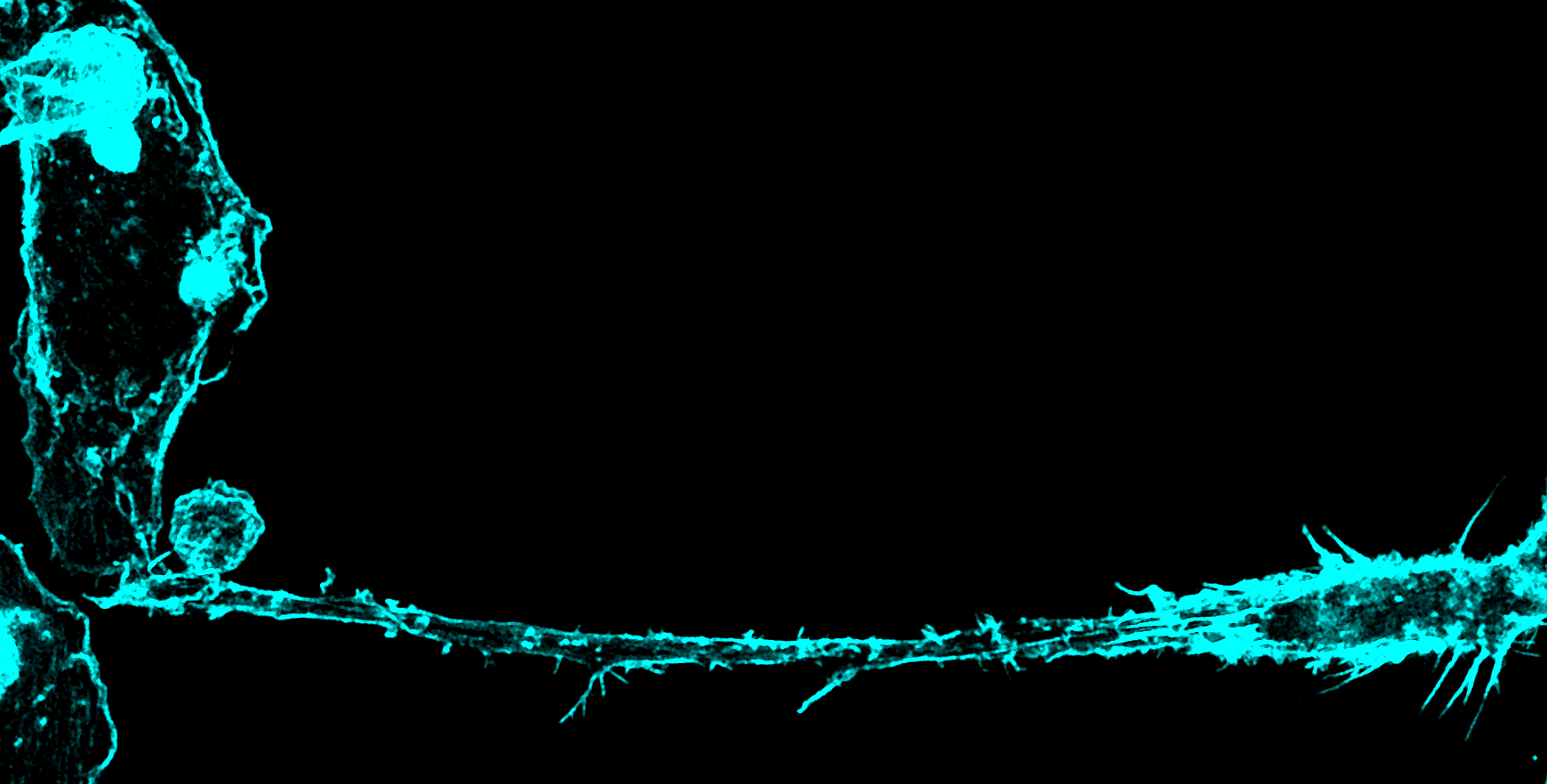

Supplement: Supplementary file 15 — Source data Fig. 6 [file 44318_2025_515_MOESM15_ESM.zip › Figure6/6C/C1-MAX_Process_03_3ADVMLE.vsi - CF_488, CF_640, CF_561-1.tif]

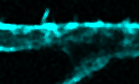

Supplement: Supplementary file 15 — Source data Fig. 6 [file 44318_2025_515_MOESM15_ESM.zip › Figure6/6C/C1-MAX_Process_03_3ADVMLE.vsi - CF_488, CF_640, CF_561-crop 2.tif]

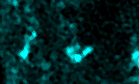

Supplement: Supplementary file 15 — Source data Fig. 6 [file 44318_2025_515_MOESM15_ESM.zip › Figure6/6C/C1-MAX_Process_03_3ADVMLE.vsi - CF_488, CF_640, CF_561-crop.tif]

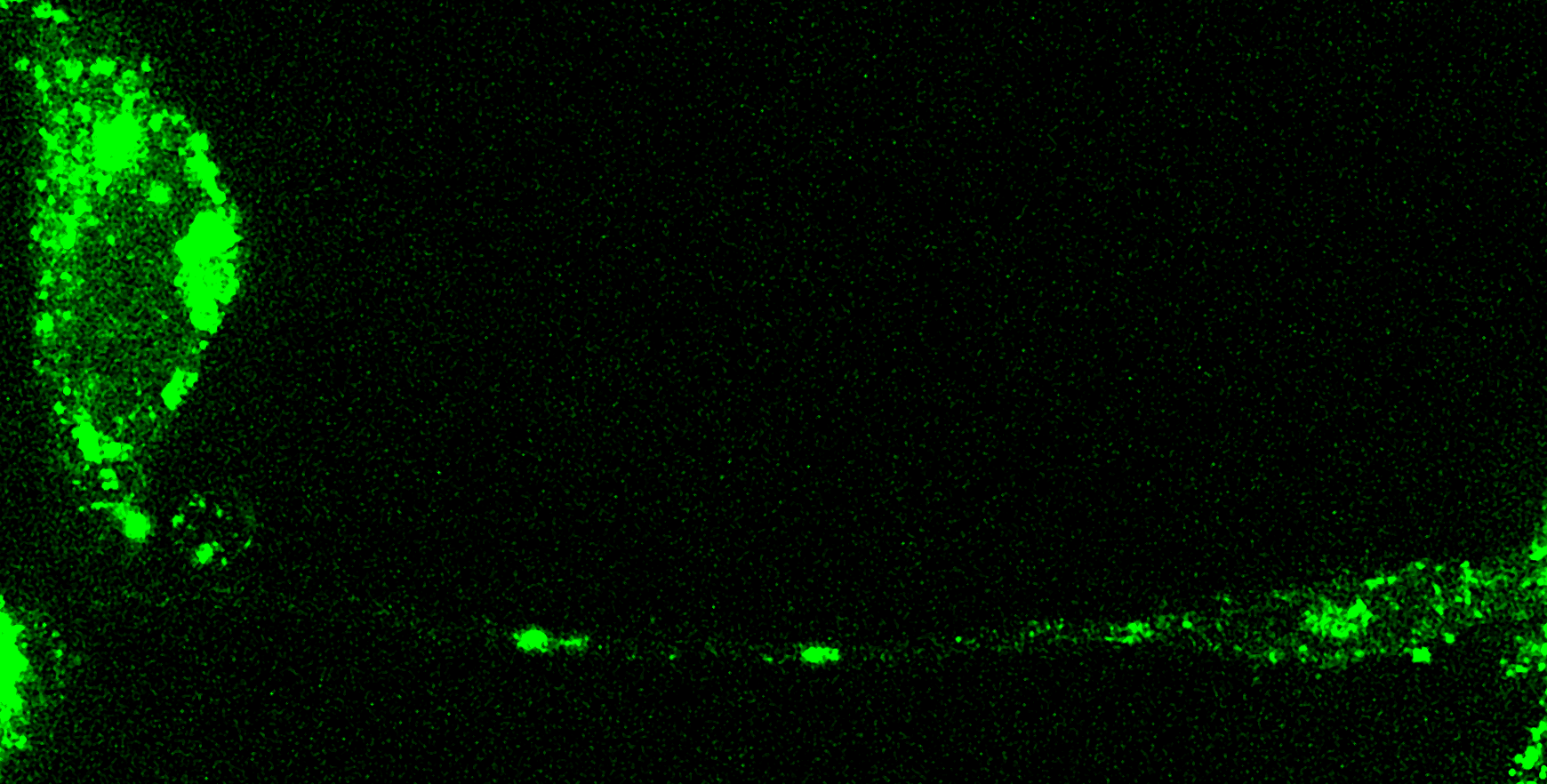

Supplement: Supplementary file 15 — Source data Fig. 6 [file 44318_2025_515_MOESM15_ESM.zip › Figure6/6C/C2-MAX_Process_03_3ADVMLE.vsi - CF_488, CF_640, CF_561-1.tif]

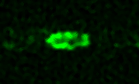

Supplement: Supplementary file 15 — Source data Fig. 6 [file 44318_2025_515_MOESM15_ESM.zip › Figure6/6C/C2-MAX_Process_03_3ADVMLE.vsi - CF_488, CF_640, CF_561-crop 2.tif]

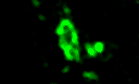

Supplement: Supplementary file 15 — Source data Fig. 6 [file 44318_2025_515_MOESM15_ESM.zip › Figure6/6C/C2-MAX_Process_03_3ADVMLE.vsi - CF_488, CF_640, CF_561-crop.tif]

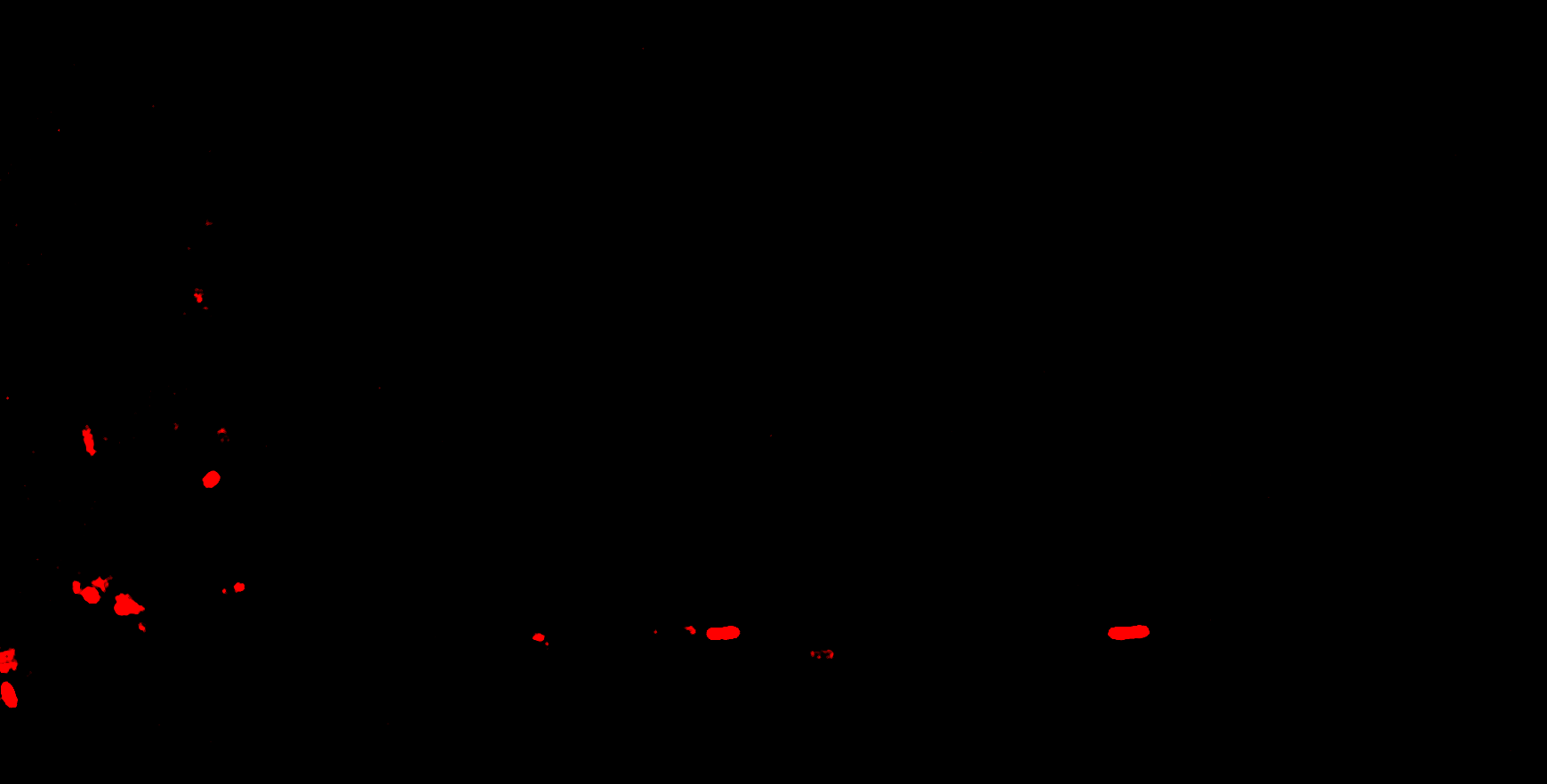

Supplement: Supplementary file 15 — Source data Fig. 6 [file 44318_2025_515_MOESM15_ESM.zip › Figure6/6C/C3-MAX_Process_03_3ADVMLE.vsi - CF_488, CF_640, CF_561-1.tif]

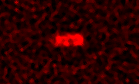

Supplement: Supplementary file 15 — Source data Fig. 6 [file 44318_2025_515_MOESM15_ESM.zip › Figure6/6C/C3-MAX_Process_03_3ADVMLE.vsi - CF_488, CF_640, CF_561-crop 2.tif]

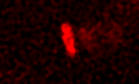

Supplement: Supplementary file 15 — Source data Fig. 6 [file 44318_2025_515_MOESM15_ESM.zip › Figure6/6C/C3-MAX_Process_03_3ADVMLE.vsi - CF_488, CF_640, CF_561-crop.tif]

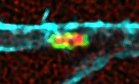

Supplement: Supplementary file 15 — Source data Fig. 6 [file 44318_2025_515_MOESM15_ESM.zip › Figure6/6C/Composite crop 2.tif]

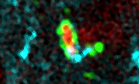

Supplement: Supplementary file 15 — Source data Fig. 6 [file 44318_2025_515_MOESM15_ESM.zip › Figure6/6C/Composite crop.tif]

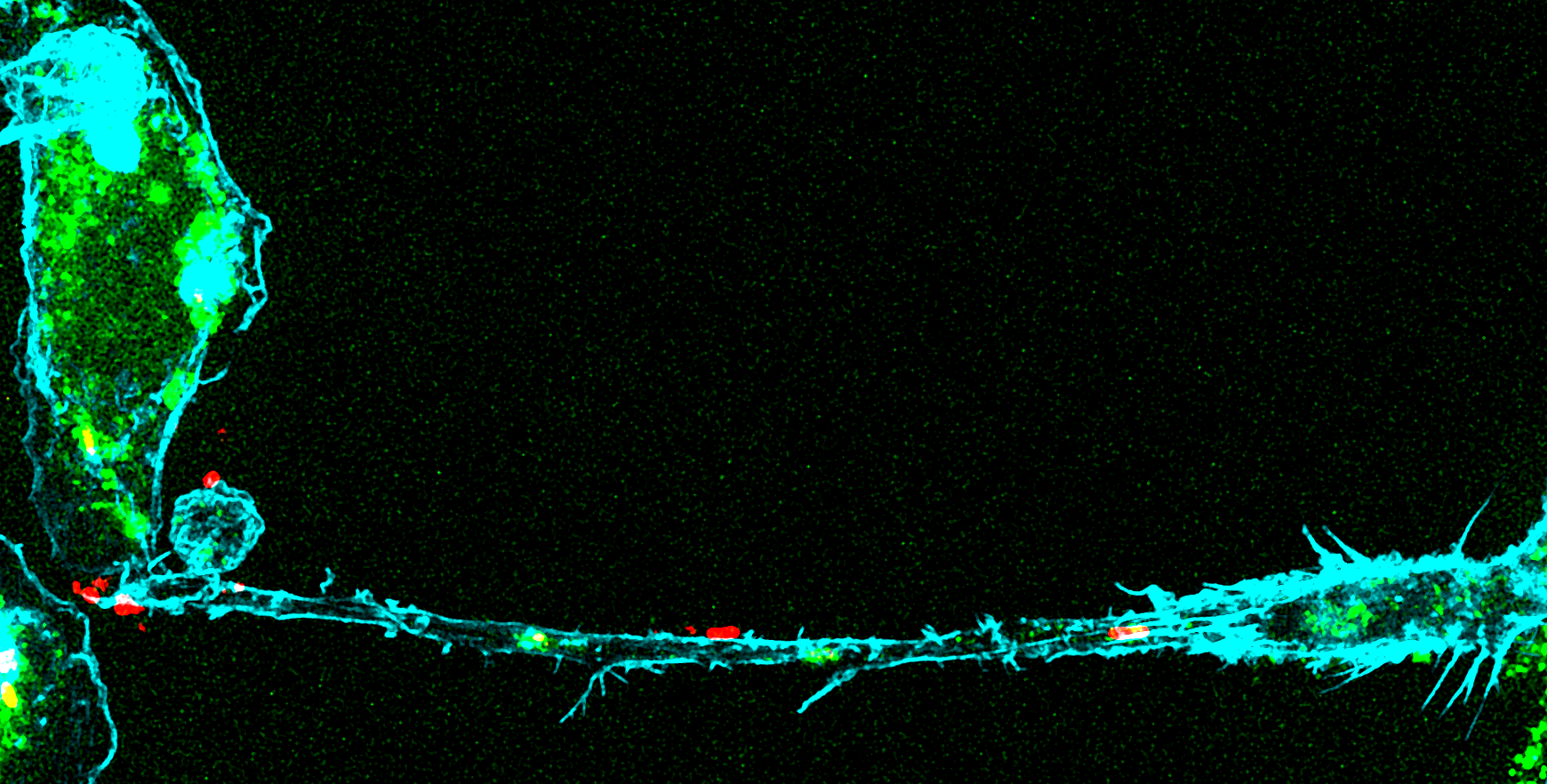

Supplement: Supplementary file 15 — Source data Fig. 6 [file 44318_2025_515_MOESM15_ESM.zip › Figure6/6C/Composite.tif]

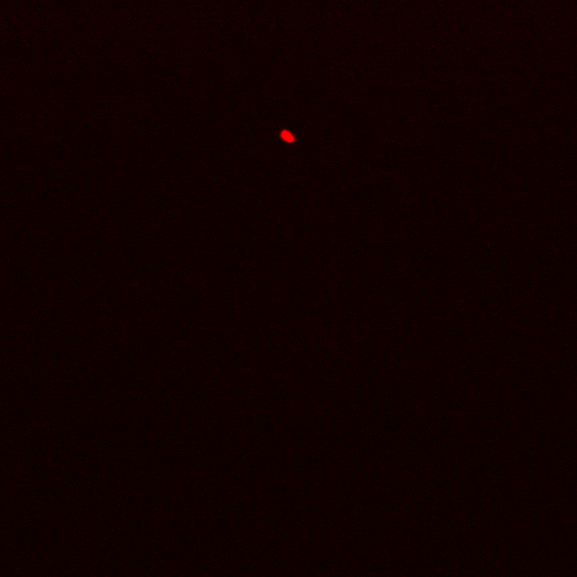

Supplement: Supplementary file 15 — Source data Fig. 6 [file 44318_2025_515_MOESM15_ESM.zip › Figure6/6D/C2-MOI=1 round.tif]

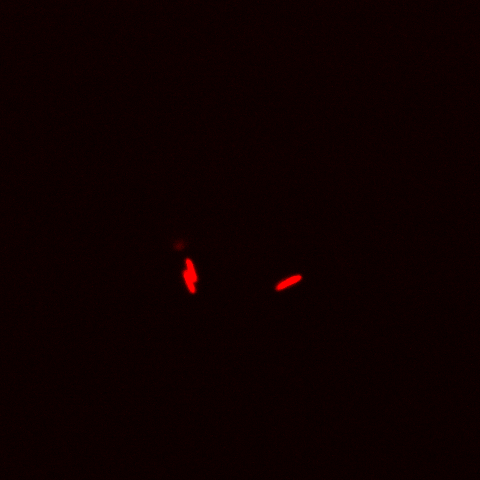

Supplement: Supplementary file 15 — Source data Fig. 6 [file 44318_2025_515_MOESM15_ESM.zip › Figure6/6D/C2-MOI=10 round.tif]

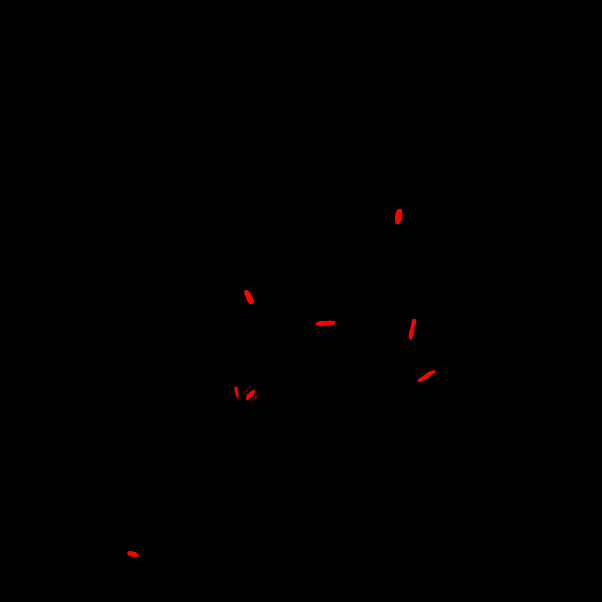

Supplement: Supplementary file 15 — Source data Fig. 6 [file 44318_2025_515_MOESM15_ESM.zip › Figure6/6D/C2-MOI=20 round.tif]

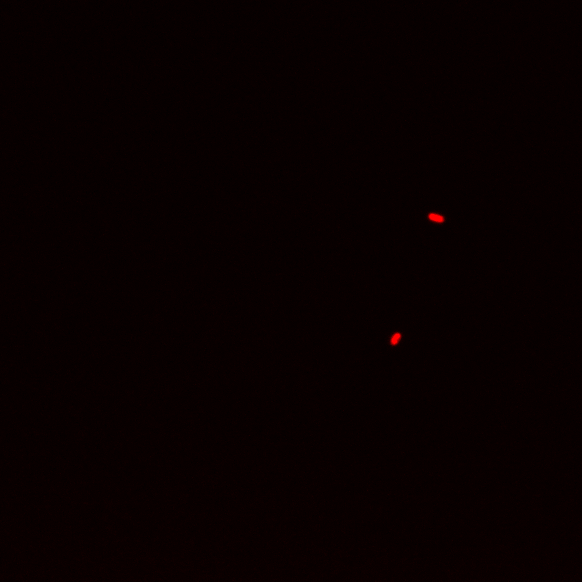

Supplement: Supplementary file 15 — Source data Fig. 6 [file 44318_2025_515_MOESM15_ESM.zip › Figure6/6D/C2-MOI=5 round.tif]

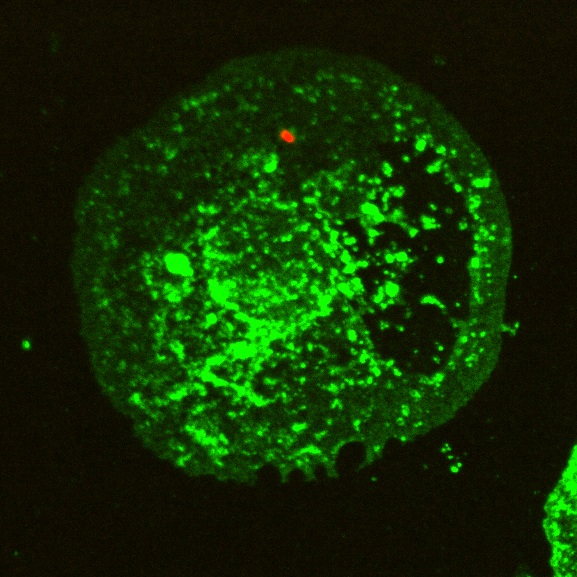

Supplement: Supplementary file 15 — Source data Fig. 6 [file 44318_2025_515_MOESM15_ESM.zip › Figure6/6D/MOI=1 round.tif (RGB).tif]

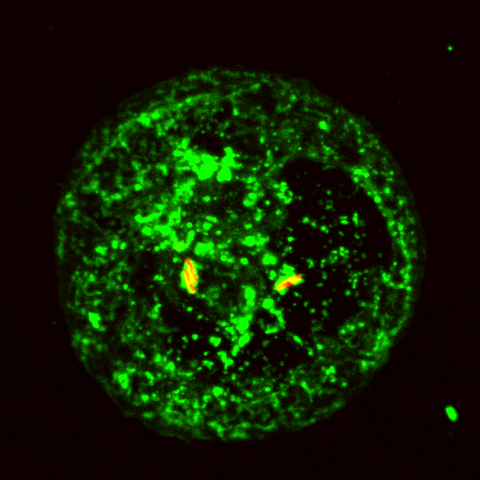

Supplement: Supplementary file 15 — Source data Fig. 6 [file 44318_2025_515_MOESM15_ESM.zip › Figure6/6D/MOI=10 round.tif (RGB).tif]

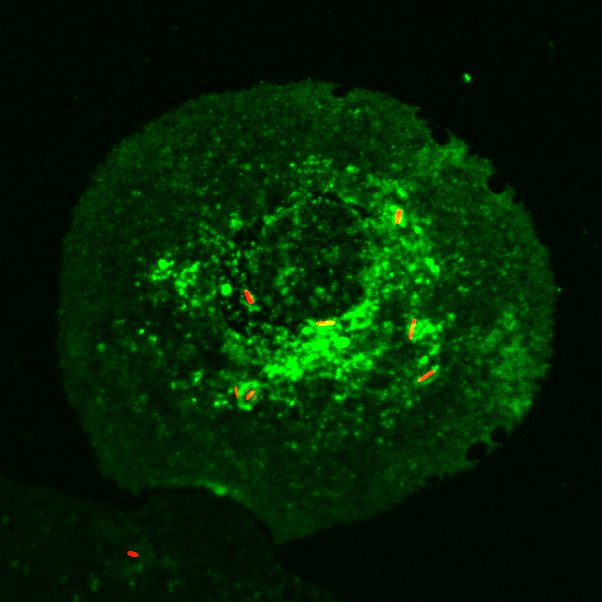

Supplement: Supplementary file 15 — Source data Fig. 6 [file 44318_2025_515_MOESM15_ESM.zip › Figure6/6D/MOI=20 round.tif (RGB).tif]

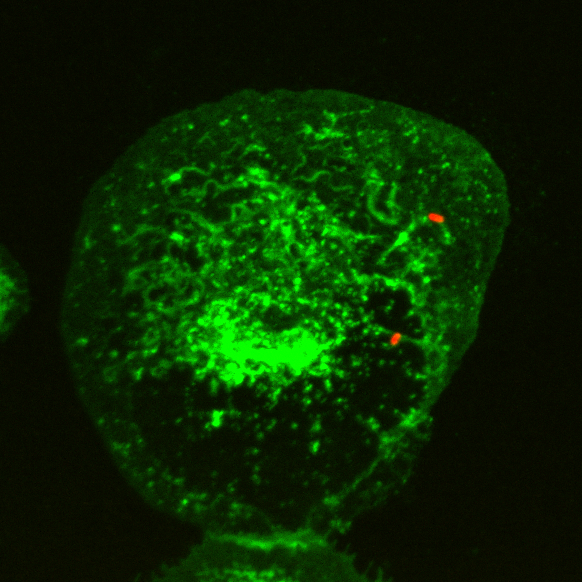

Supplement: Supplementary file 15 — Source data Fig. 6 [file 44318_2025_515_MOESM15_ESM.zip › Figure6/6D/MOI=5 round.tif (RGB).tif]

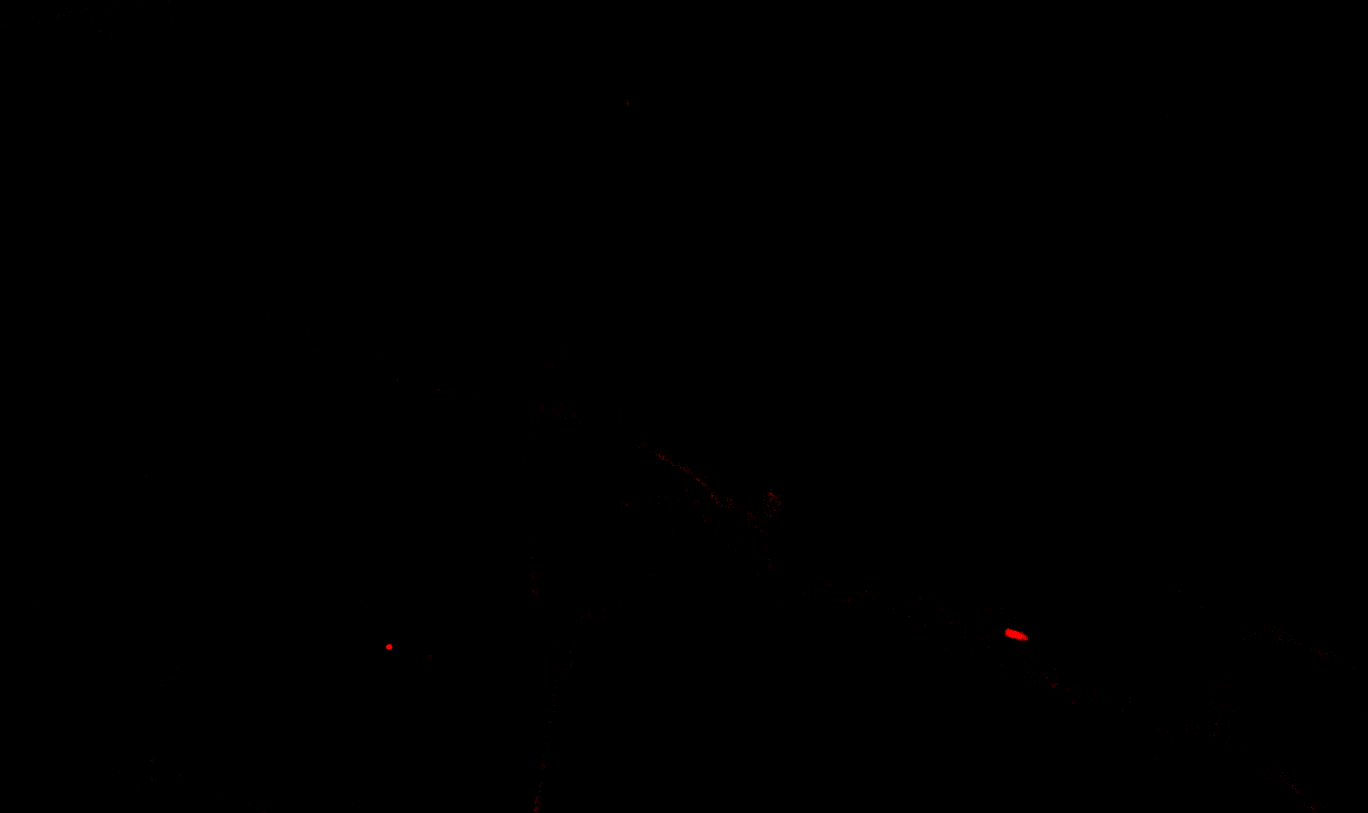

Supplement: Supplementary file 15 — Source data Fig. 6 [file 44318_2025_515_MOESM15_ESM.zip › Figure6/6E/C1-MOI=1 macropodia.tif]

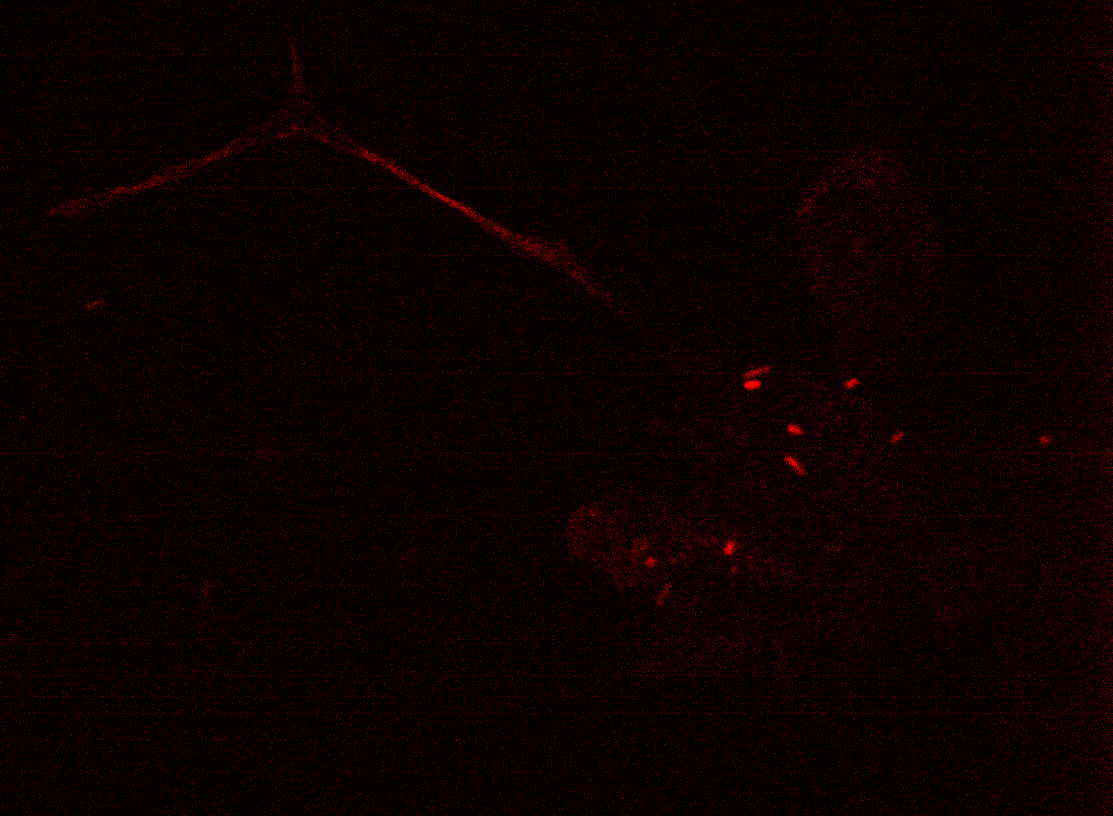

Supplement: Supplementary file 15 — Source data Fig. 6 [file 44318_2025_515_MOESM15_ESM.zip › Figure6/6E/C1-MOI=10 macropodia.tif]

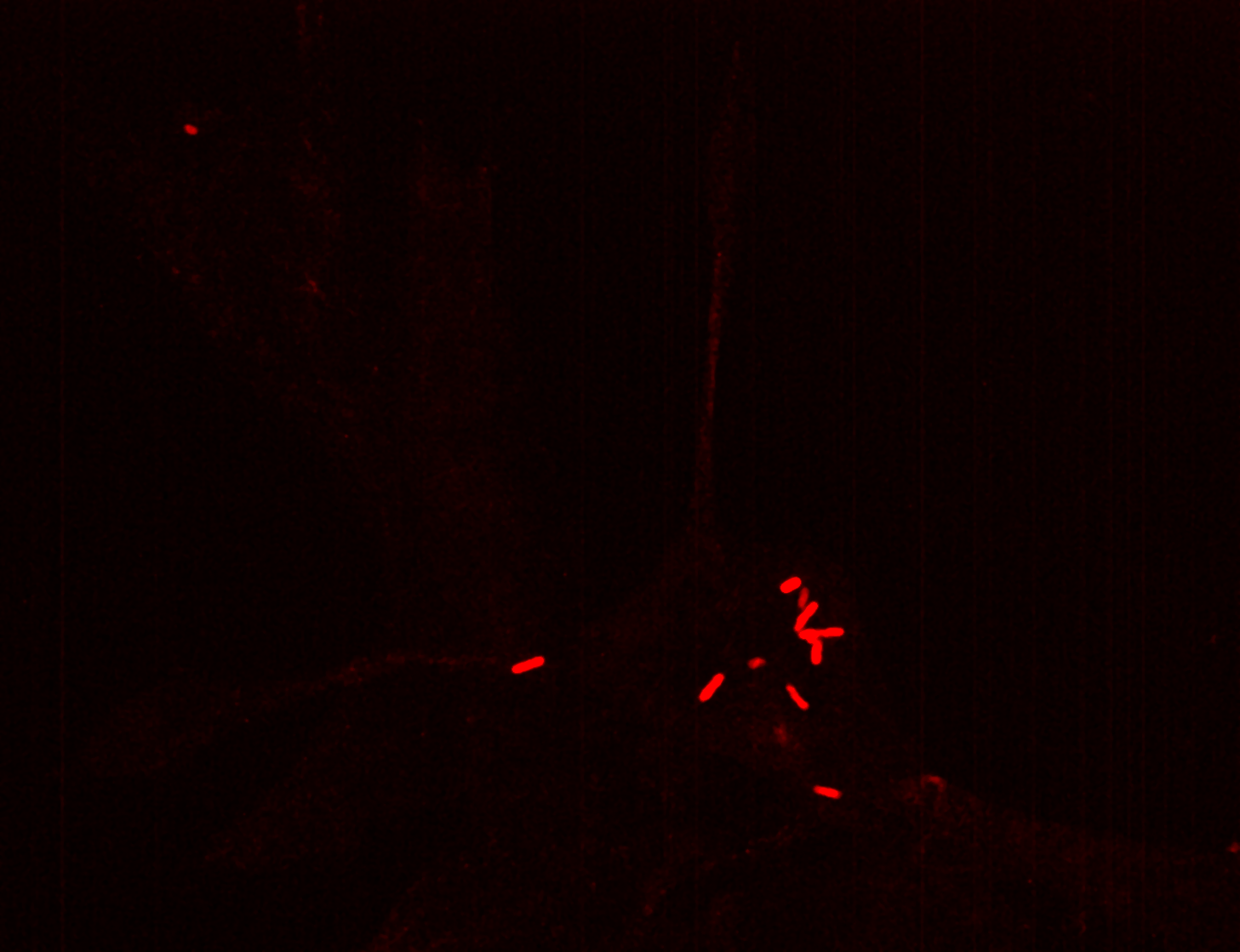

Supplement: Supplementary file 15 — Source data Fig. 6 [file 44318_2025_515_MOESM15_ESM.zip › Figure6/6E/C1-MOI=20 macropodia.tif]

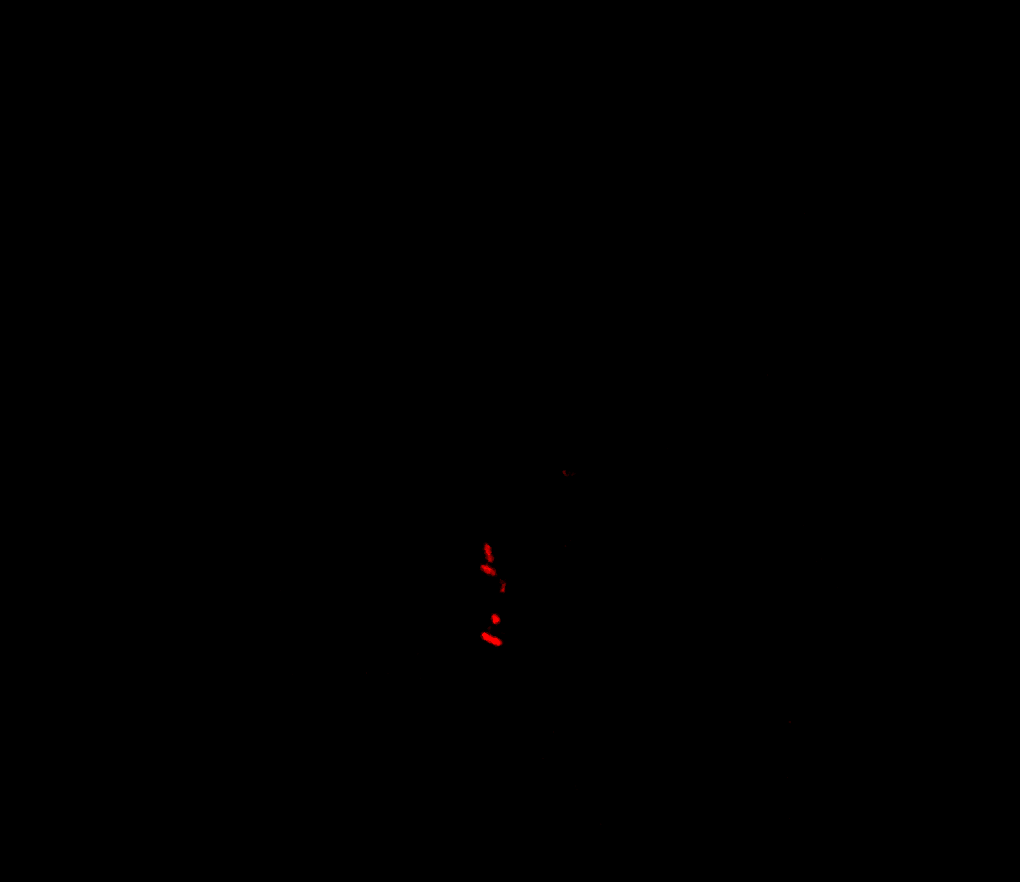

Supplement: Supplementary file 15 — Source data Fig. 6 [file 44318_2025_515_MOESM15_ESM.zip › Figure6/6E/C1-MOI=5 macropodia.tif]

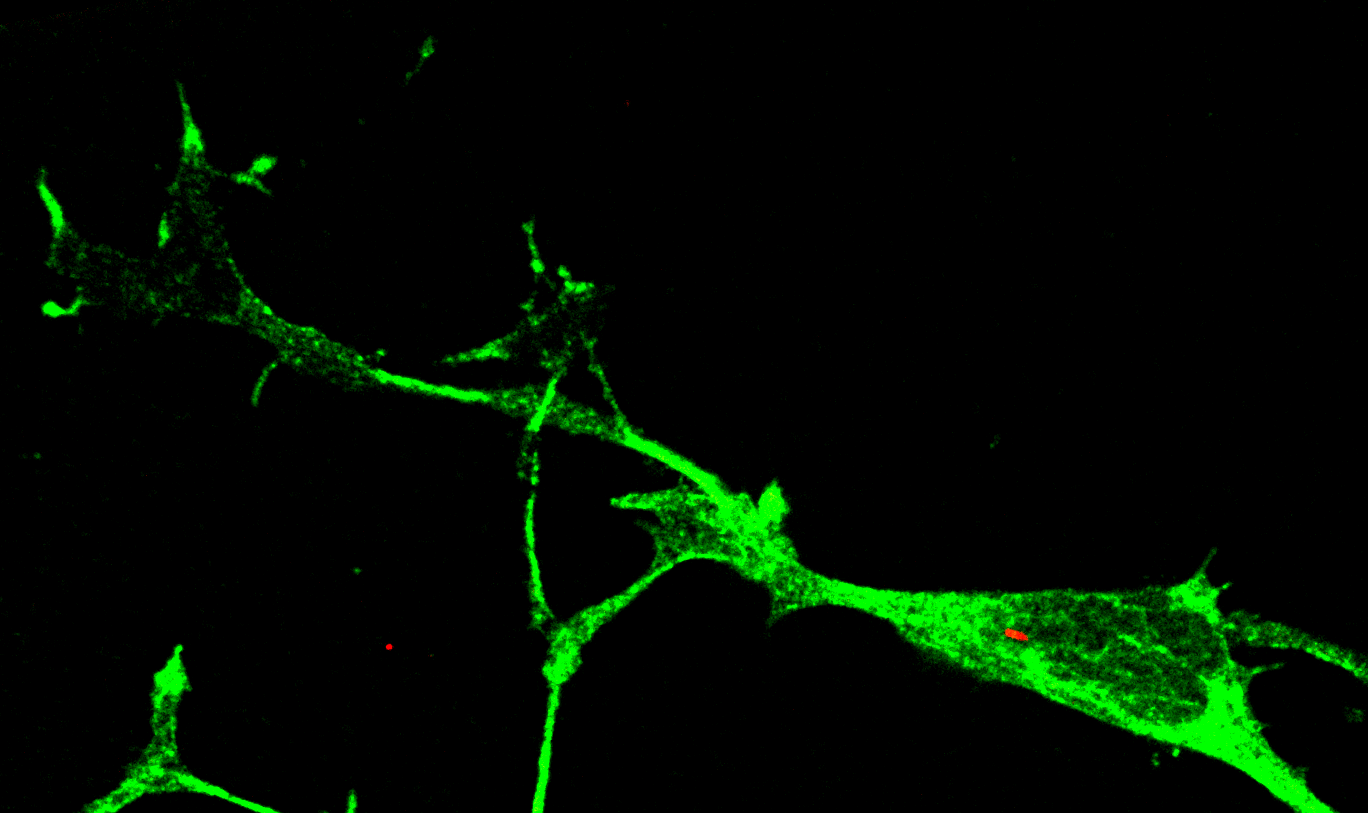

Supplement: Supplementary file 15 — Source data Fig. 6 [file 44318_2025_515_MOESM15_ESM.zip › Figure6/6E/MOI=1 macropodia.tif (RGB).tif]

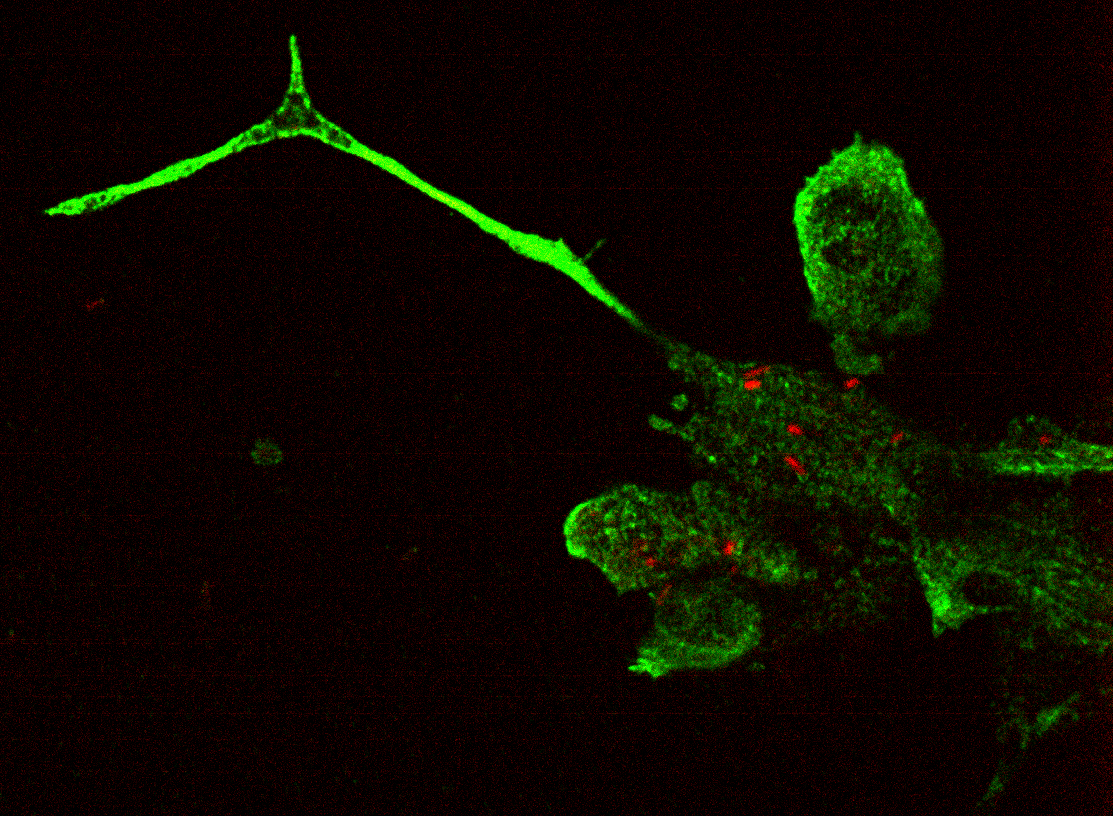

Supplement: Supplementary file 15 — Source data Fig. 6 [file 44318_2025_515_MOESM15_ESM.zip › Figure6/6E/MOI=10 macropodia.tif (RGB).tif]

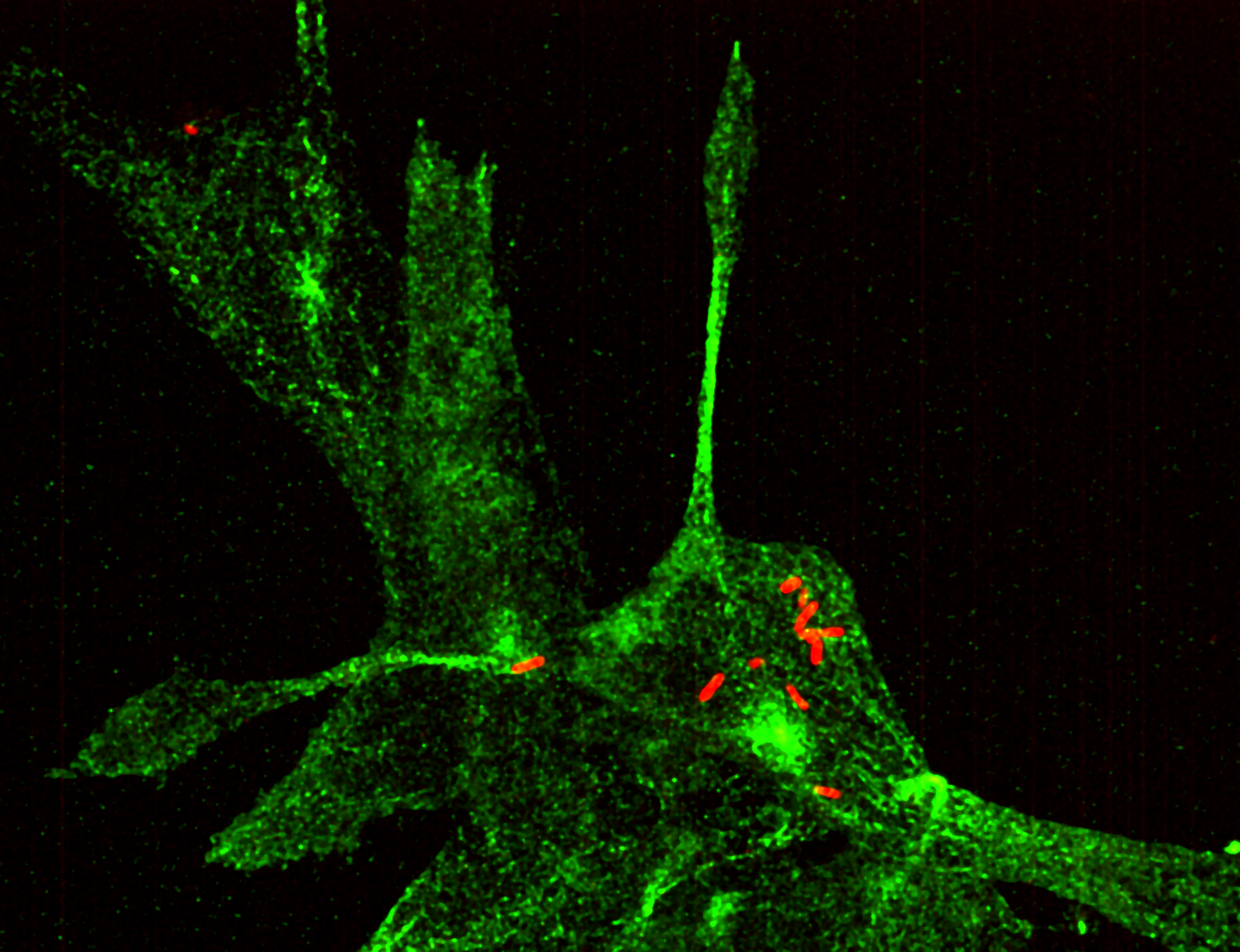

Supplement: Supplementary file 15 — Source data Fig. 6 [file 44318_2025_515_MOESM15_ESM.zip › Figure6/6E/MOI=20 macropodia.tif (RGB).tif]

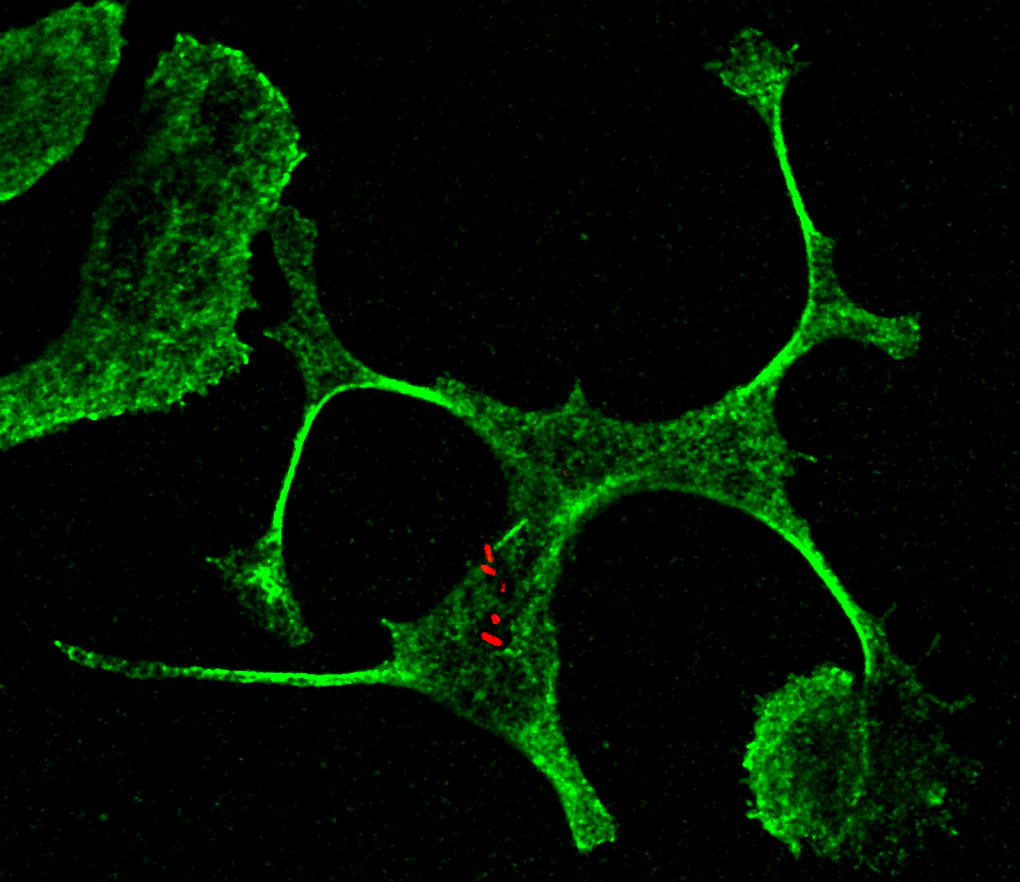

Supplement: Supplementary file 15 — Source data Fig. 6 [file 44318_2025_515_MOESM15_ESM.zip › Figure6/6E/MOI=5 macropodia.tif (RGB).tif]
