# Supplementary figures and images for: Macrophages form dendrite-like pseudopods to enhance bacterial ingestion (part 3 of 3)
Source: EMBO J. 2025 Jul 28;44(17):4772–802. doi: 10.1038/s44318-025-00515-z (PMC12402336; doi:10.1038/s44318-025-00515-z)

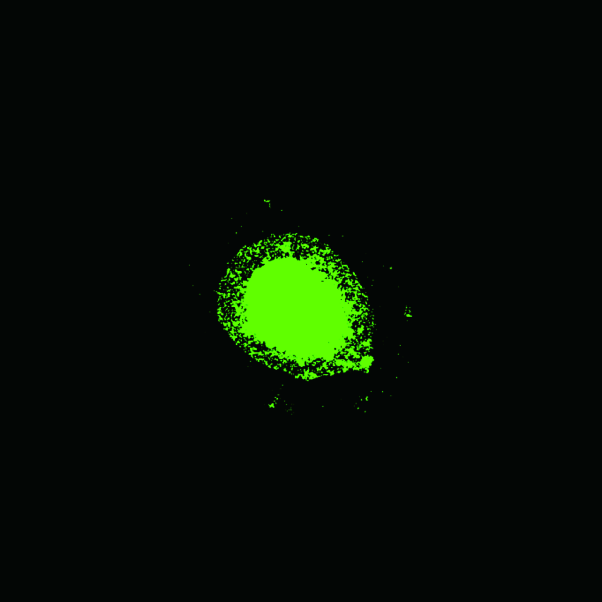

Supplement: Supplementary file 16 — Figure EV1 Source Data [file 44318_2025_515_MOESM16_ESM.zip › FigureEV1/1F/sholl shape I.tif]

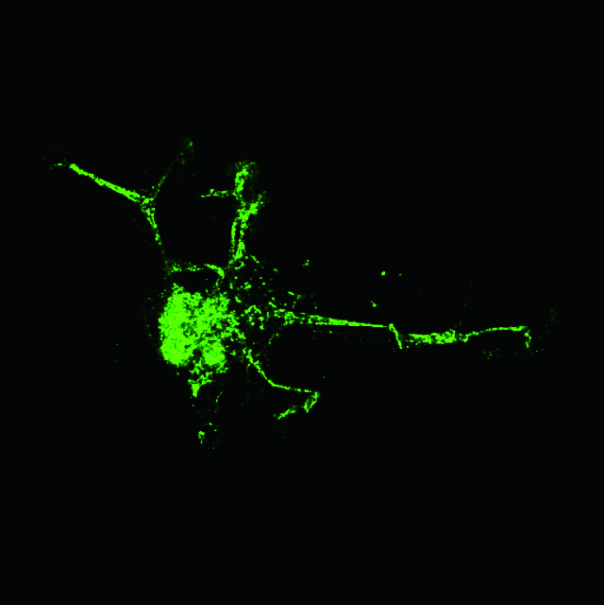

Supplement: Supplementary file 16 — Figure EV1 Source Data [file 44318_2025_515_MOESM16_ESM.zip › FigureEV1/1F/sholl shape II.tif]

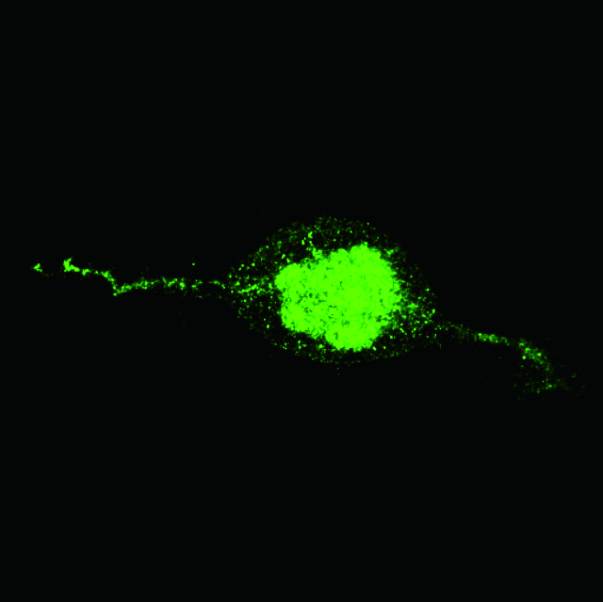

Supplement: Supplementary file 16 — Figure EV1 Source Data [file 44318_2025_515_MOESM16_ESM.zip › FigureEV1/1F/sholl shape III.tif]

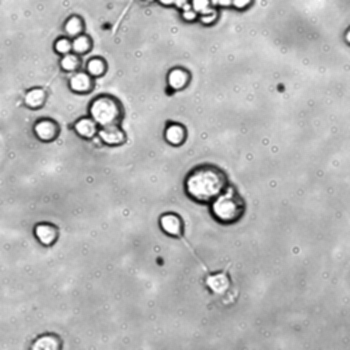

Supplement: Supplementary file 16 — Figure EV1 Source Data [file 44318_2025_515_MOESM16_ESM.zip › FigureEV1/1G/Cell debris 0min.tif]

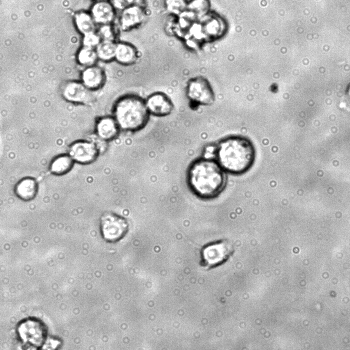

Supplement: Supplementary file 16 — Figure EV1 Source Data [file 44318_2025_515_MOESM16_ESM.zip › FigureEV1/1G/Cell debris 288min.tif]

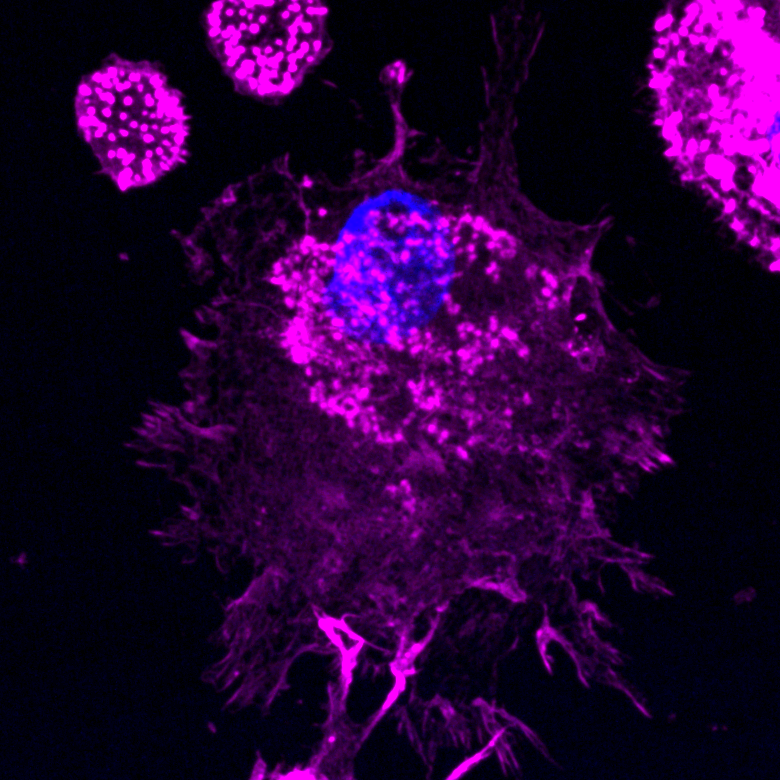

Supplement: Supplementary file 16 — Figure EV1 Source Data [file 44318_2025_515_MOESM16_ESM.zip › FigureEV1/1I/MoDC infection 1.tif]

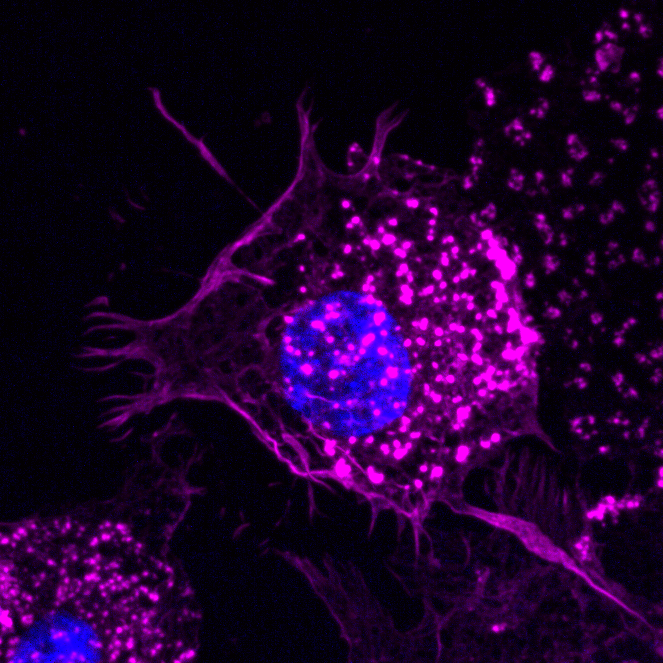

Supplement: Supplementary file 16 — Figure EV1 Source Data [file 44318_2025_515_MOESM16_ESM.zip › FigureEV1/1I/MoDC infection 2.tif]

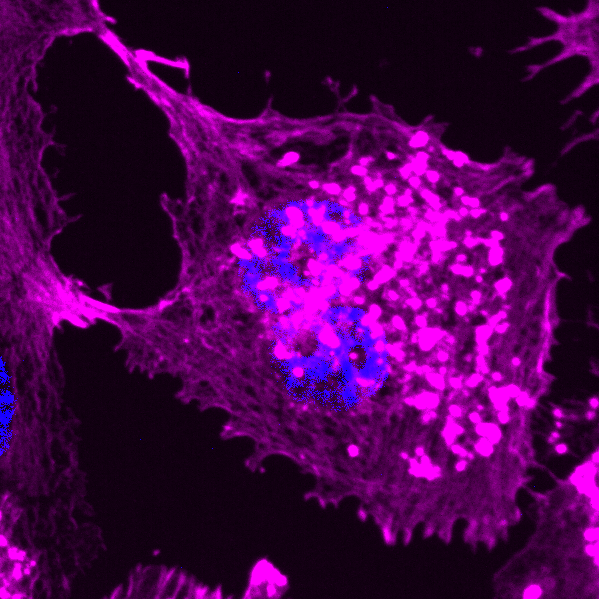

Supplement: Supplementary file 16 — Figure EV1 Source Data [file 44318_2025_515_MOESM16_ESM.zip › FigureEV1/1I/MoDC infection 3.tif]

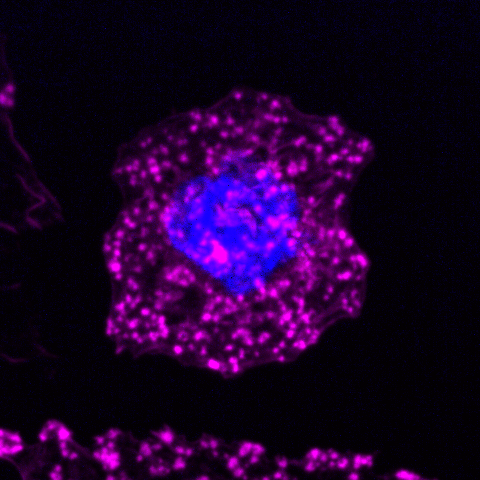

Supplement: Supplementary file 16 — Figure EV1 Source Data [file 44318_2025_515_MOESM16_ESM.zip › FigureEV1/1I/MoDC mock 2.tif]

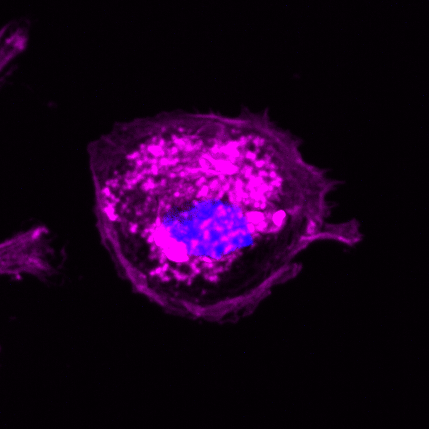

Supplement: Supplementary file 16 — Figure EV1 Source Data [file 44318_2025_515_MOESM16_ESM.zip › FigureEV1/1I/MoDC mock 3.tif]

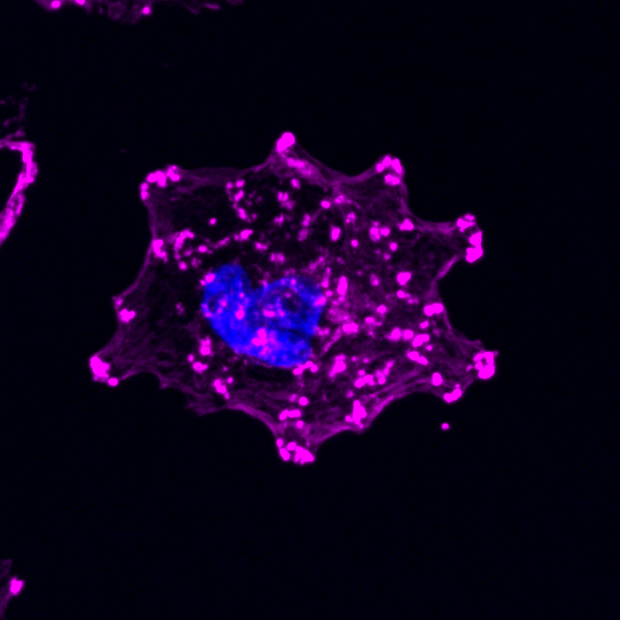

Supplement: Supplementary file 16 — Figure EV1 Source Data [file 44318_2025_515_MOESM16_ESM.zip › FigureEV1/1I/MoDC mock1.tif]

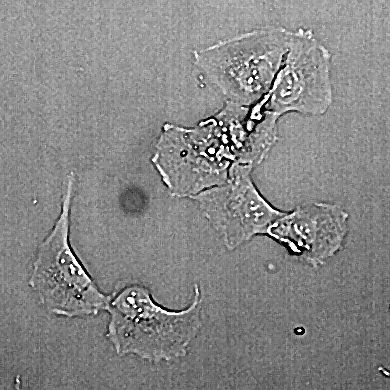

Supplement: Supplementary file 16 — Figure EV1 Source Data [file 44318_2025_515_MOESM16_ESM.zip › FigureEV1/1K/U2OS-0 min.tif]

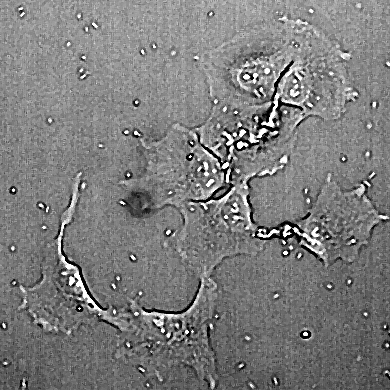

Supplement: Supplementary file 16 — Figure EV1 Source Data [file 44318_2025_515_MOESM16_ESM.zip › FigureEV1/1K/U2OS-180 min.tif]

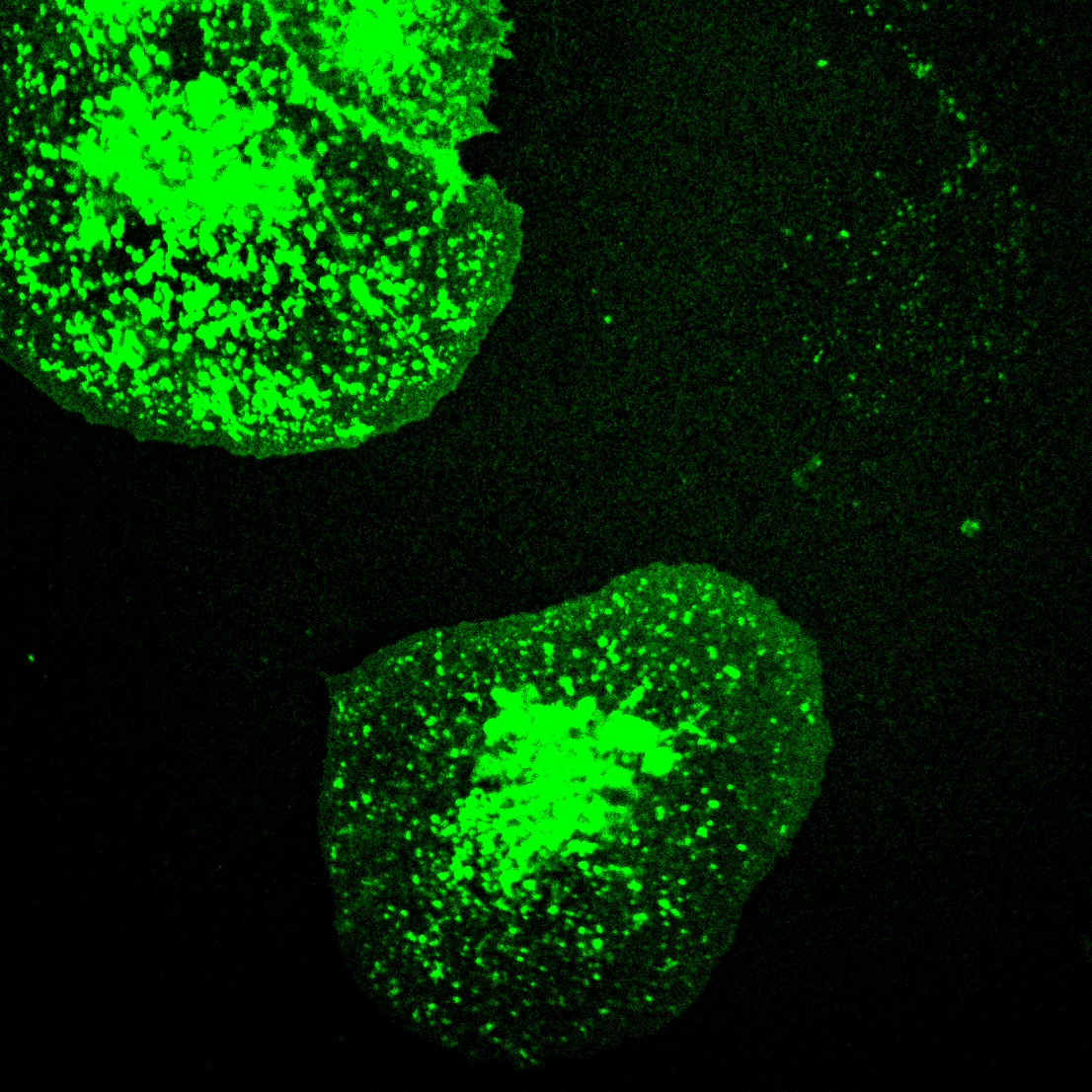

Supplement: Supplementary file 16 — Figure EV1 Source Data [file 44318_2025_515_MOESM16_ESM.zip › FigureEV1/1L/DLP 0min.tif]

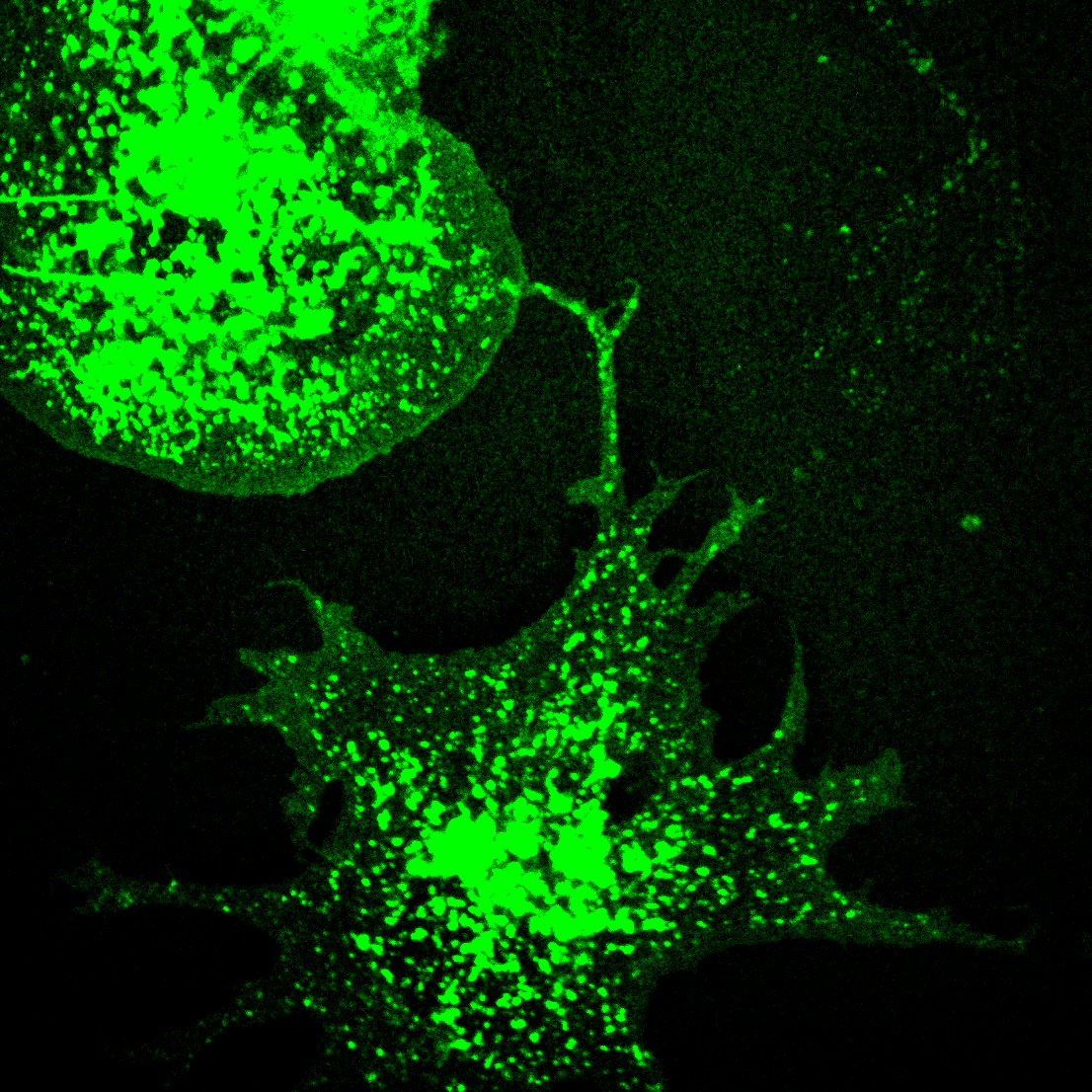

Supplement: Supplementary file 16 — Figure EV1 Source Data [file 44318_2025_515_MOESM16_ESM.zip › FigureEV1/1L/DLP 384min.tif]

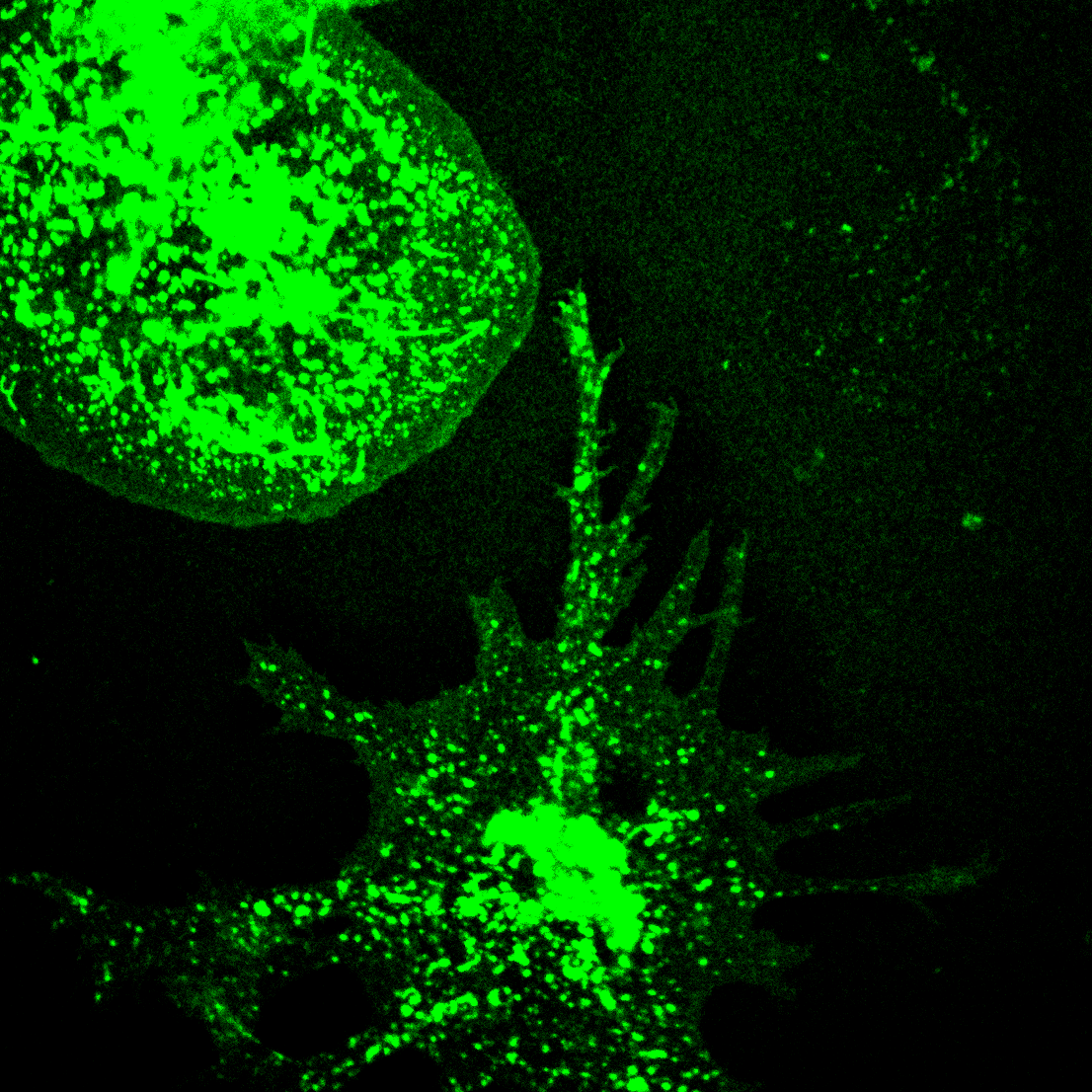

Supplement: Supplementary file 16 — Figure EV1 Source Data [file 44318_2025_515_MOESM16_ESM.zip › FigureEV1/1L/DLP 476min.tif]

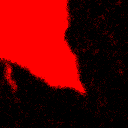

Supplement: Supplementary file 16 — Figure EV1 Source Data [file 44318_2025_515_MOESM16_ESM.zip › FigureEV1/1L/filopodia example 4min interval 0min.tif]

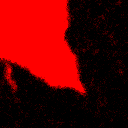

Supplement: Supplementary file 16 — Figure EV1 Source Data [file 44318_2025_515_MOESM16_ESM.zip › FigureEV1/1L/filopodia example 4min interval 12min.tif]

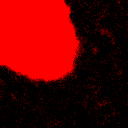

Supplement: Supplementary file 16 — Figure EV1 Source Data [file 44318_2025_515_MOESM16_ESM.zip › FigureEV1/1L/filopodia example 4min interval 32min.tif]

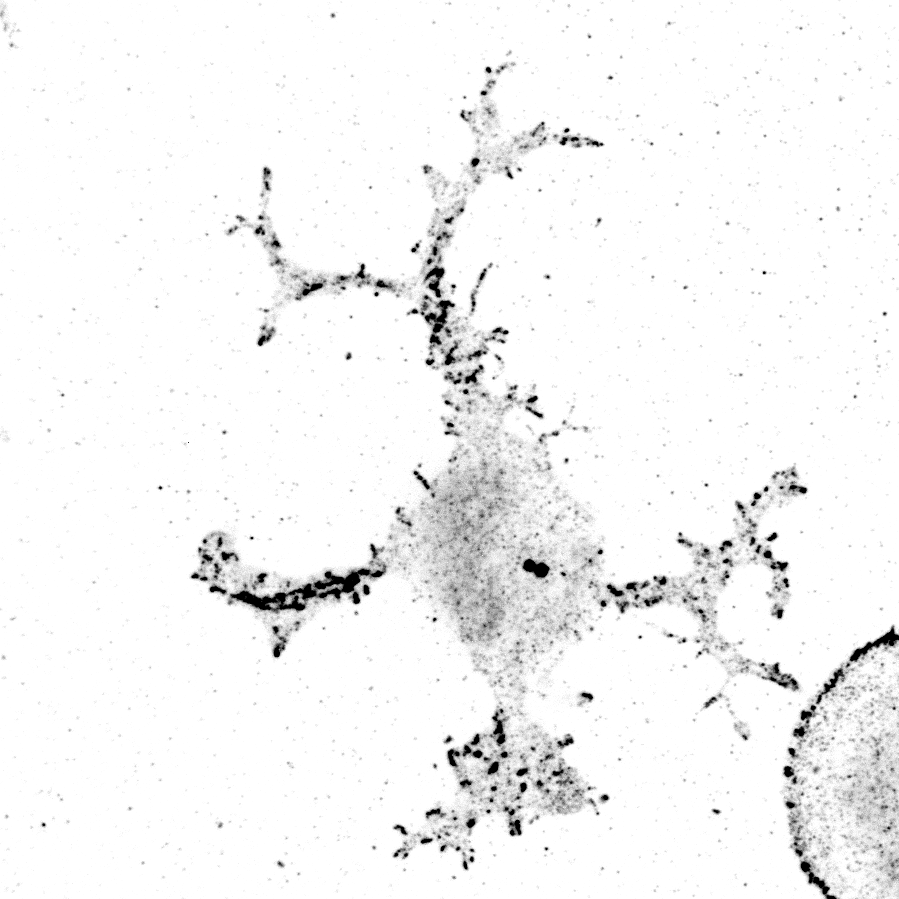

Supplement: Supplementary file 16 — Figure EV1 Source Data [file 44318_2025_515_MOESM16_ESM.zip › FigureEV1/1N/vinculin 2.tif]

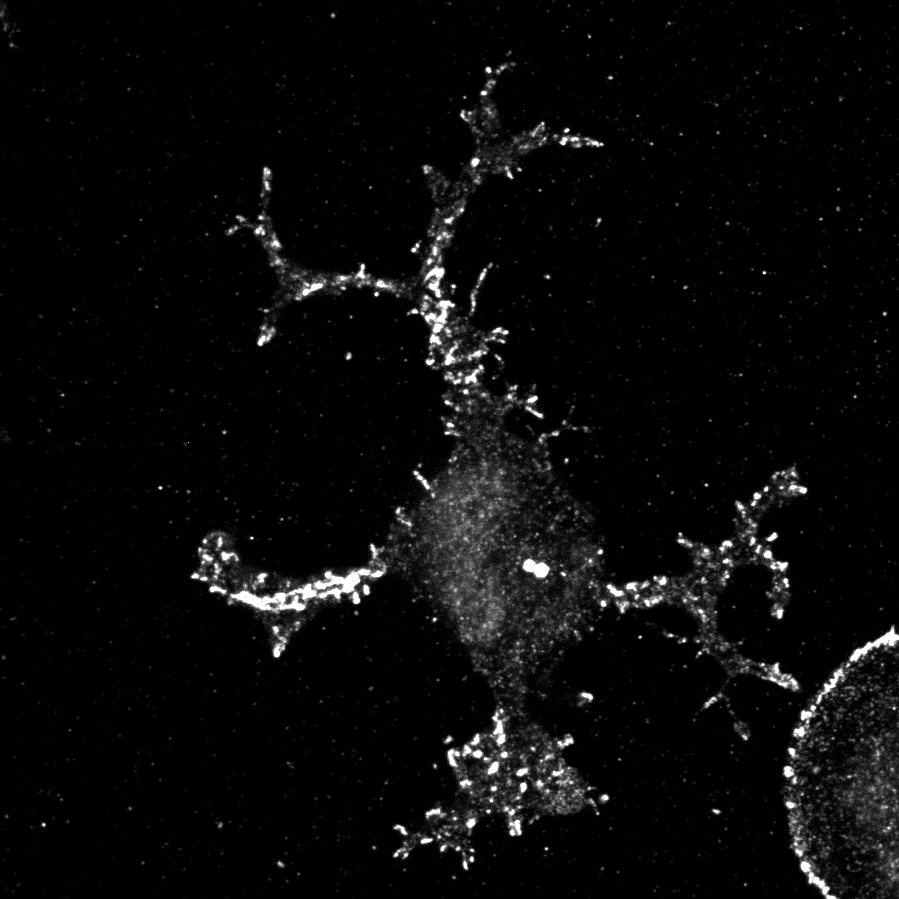

Supplement: Supplementary file 16 — Figure EV1 Source Data [file 44318_2025_515_MOESM16_ESM.zip › FigureEV1/1N/vinculin RAW data.tif]

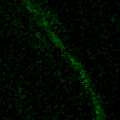

Supplement: Supplementary file 17 — Figure EV2 Source Data [file 44318_2025_515_MOESM17_ESM.zip › FigureEV2/2D/Bleb-1.tif]

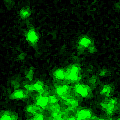

Supplement: Supplementary file 17 — Figure EV2 Source Data [file 44318_2025_515_MOESM17_ESM.zip › FigureEV2/2D/Bleb-2.tif]

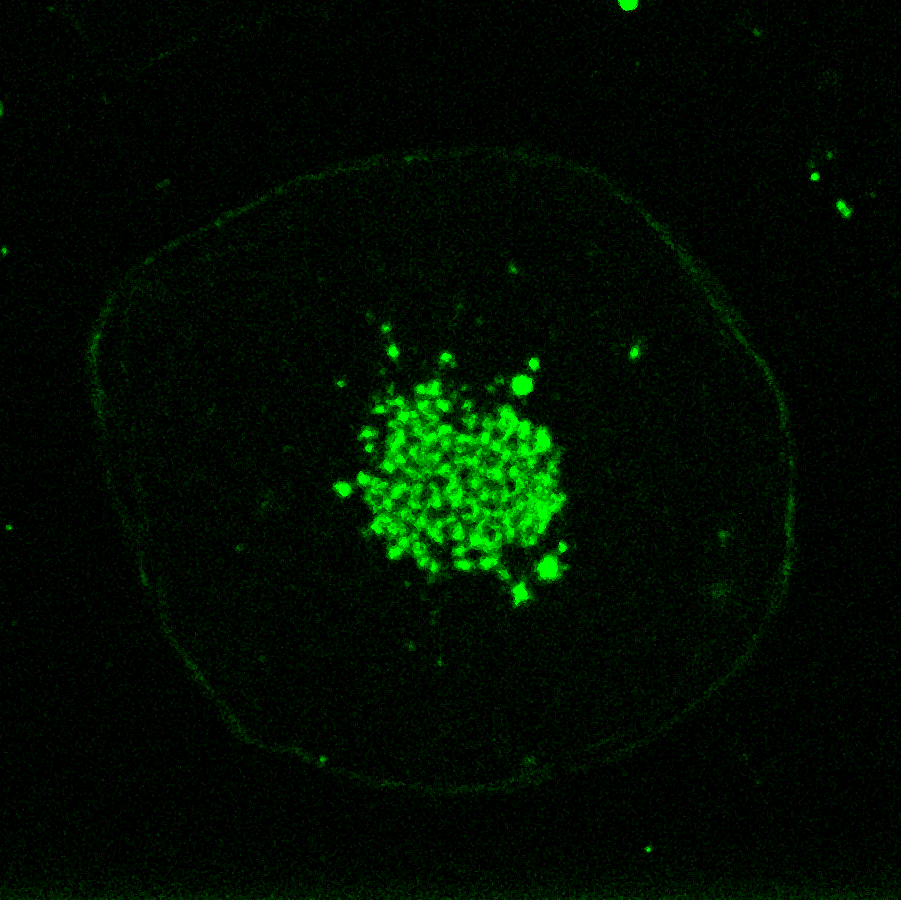

Supplement: Supplementary file 17 — Figure EV2 Source Data [file 44318_2025_515_MOESM17_ESM.zip › FigureEV2/2D/Bleb.tif]

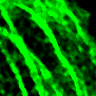

Supplement: Supplementary file 17 — Figure EV2 Source Data [file 44318_2025_515_MOESM17_ESM.zip › FigureEV2/2D/CK666-1.tif]

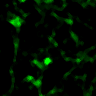

Supplement: Supplementary file 17 — Figure EV2 Source Data [file 44318_2025_515_MOESM17_ESM.zip › FigureEV2/2D/CK666-2.tif]

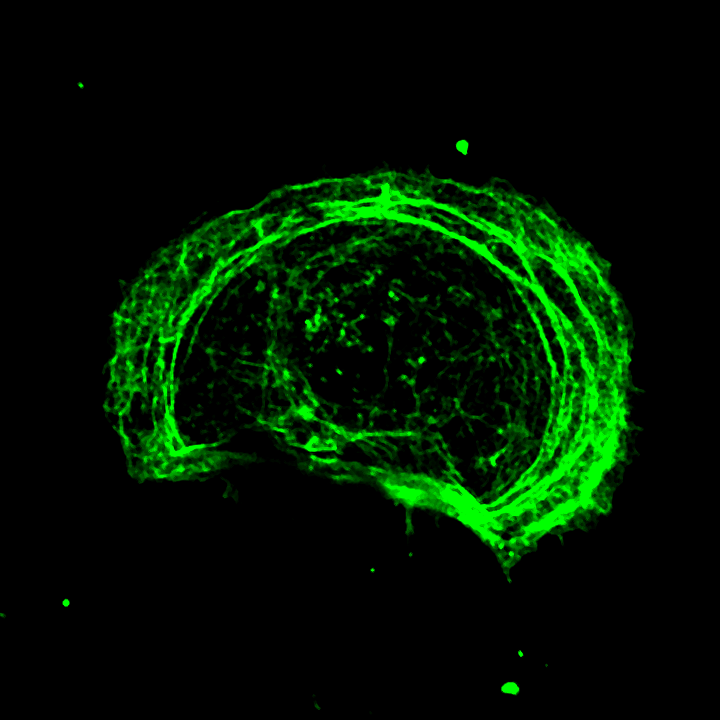

Supplement: Supplementary file 17 — Figure EV2 Source Data [file 44318_2025_515_MOESM17_ESM.zip › FigureEV2/2D/CK666.tif]

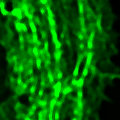

Supplement: Supplementary file 17 — Figure EV2 Source Data [file 44318_2025_515_MOESM17_ESM.zip › FigureEV2/2D/Ctrl-1.tif]

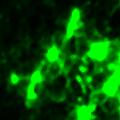

Supplement: Supplementary file 17 — Figure EV2 Source Data [file 44318_2025_515_MOESM17_ESM.zip › FigureEV2/2D/Ctrl-2.tif]

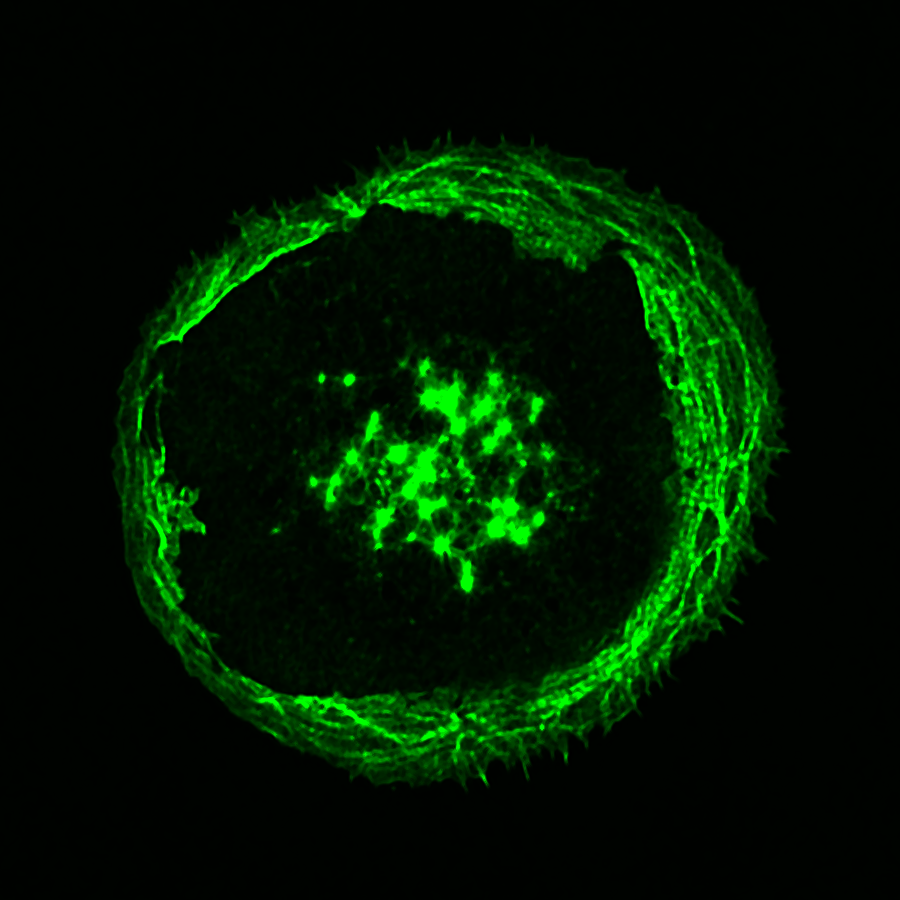

Supplement: Supplementary file 17 — Figure EV2 Source Data [file 44318_2025_515_MOESM17_ESM.zip › FigureEV2/2D/Ctrl.tif]

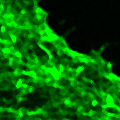

Supplement: Supplementary file 17 — Figure EV2 Source Data [file 44318_2025_515_MOESM17_ESM.zip › FigureEV2/2D/LatB-1.tif]

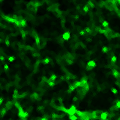

Supplement: Supplementary file 17 — Figure EV2 Source Data [file 44318_2025_515_MOESM17_ESM.zip › FigureEV2/2D/LatB-2.tif]

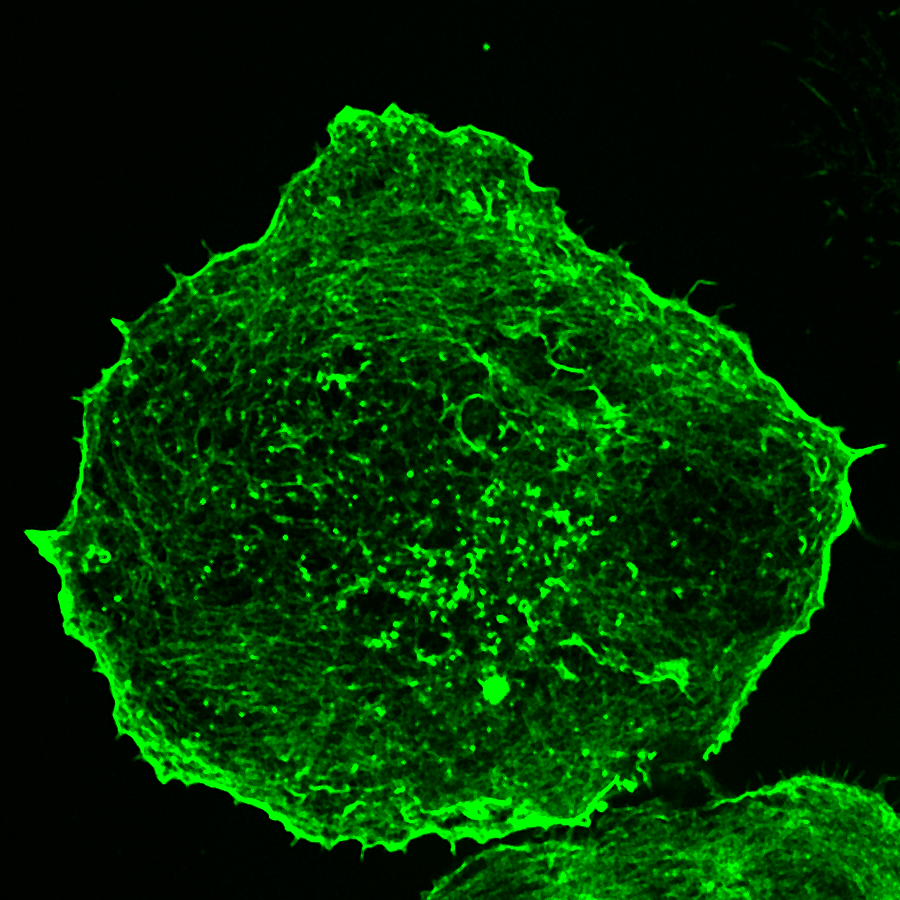

Supplement: Supplementary file 17 — Figure EV2 Source Data [file 44318_2025_515_MOESM17_ESM.zip › FigureEV2/2D/LatB.tif]

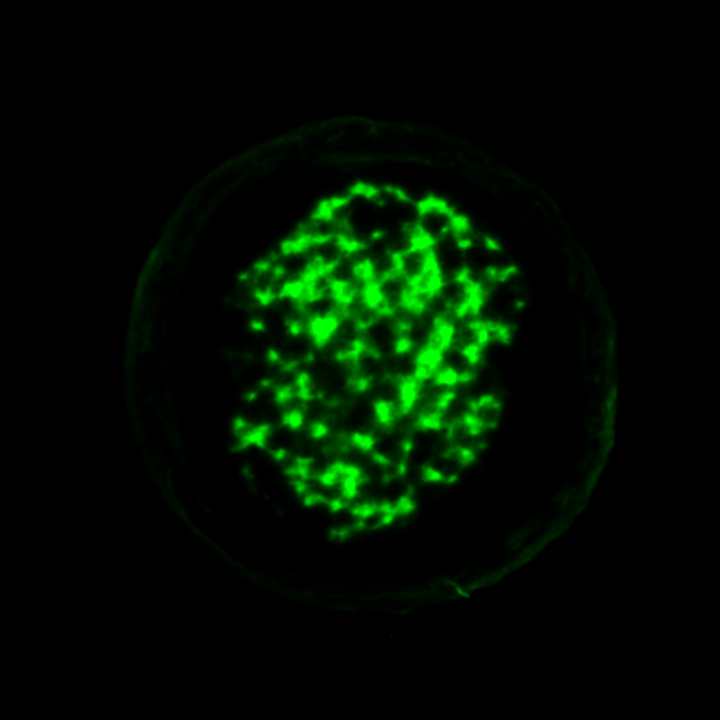

Supplement: Supplementary file 17 — Figure EV2 Source Data [file 44318_2025_515_MOESM17_ESM.zip › FigureEV2/2D/NP-G2-044.tif]

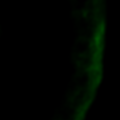

Supplement: Supplementary file 17 — Figure EV2 Source Data [file 44318_2025_515_MOESM17_ESM.zip › FigureEV2/2D/NP-G3-044-1.tif]

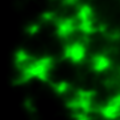

Supplement: Supplementary file 17 — Figure EV2 Source Data [file 44318_2025_515_MOESM17_ESM.zip › FigureEV2/2D/NP-G3-044.tif]

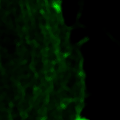

Supplement: Supplementary file 17 — Figure EV2 Source Data [file 44318_2025_515_MOESM17_ESM.zip › FigureEV2/2D/SMITFH-2.tif]

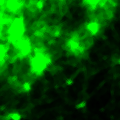

Supplement: Supplementary file 17 — Figure EV2 Source Data [file 44318_2025_515_MOESM17_ESM.zip › FigureEV2/2D/SMITFH-3.tif]

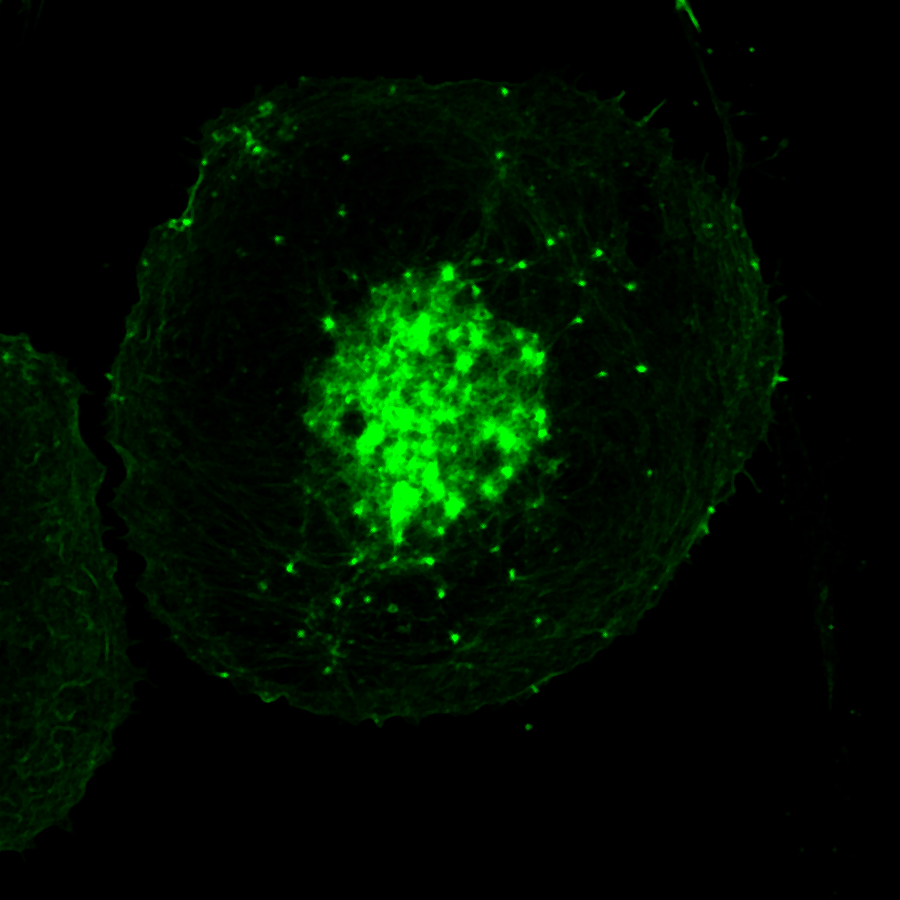

Supplement: Supplementary file 17 — Figure EV2 Source Data [file 44318_2025_515_MOESM17_ESM.zip › FigureEV2/2D/SMITFH.tif]

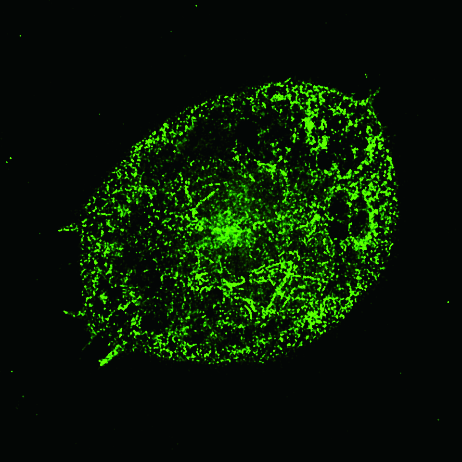

Supplement: Supplementary file 17 — Figure EV2 Source Data [file 44318_2025_515_MOESM17_ESM.zip › FigureEV2/2E/Nocdazole tubulin.tif]

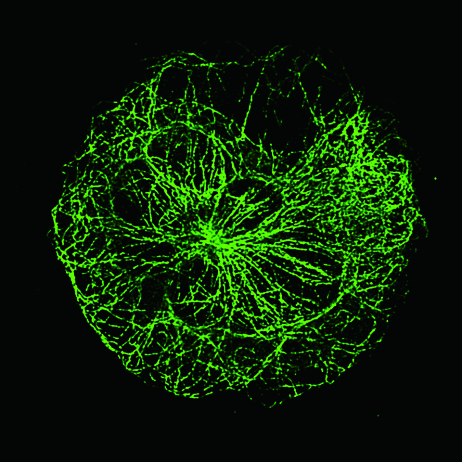

Supplement: Supplementary file 17 — Figure EV2 Source Data [file 44318_2025_515_MOESM17_ESM.zip › FigureEV2/2E/Veh tubulin.tif]

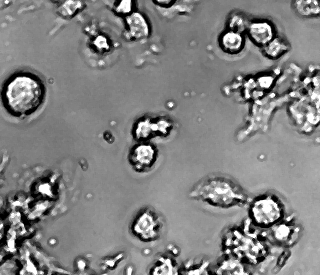

Supplement: Supplementary file 17 — Figure EV2 Source Data [file 44318_2025_515_MOESM17_ESM.zip › FigureEV2/2G/Noc-0min.tif]

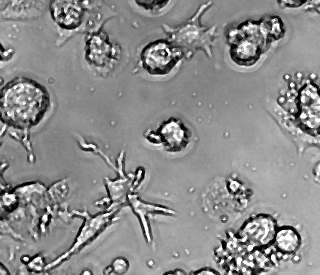

Supplement: Supplementary file 17 — Figure EV2 Source Data [file 44318_2025_515_MOESM17_ESM.zip › FigureEV2/2G/Noc-180min.tif]

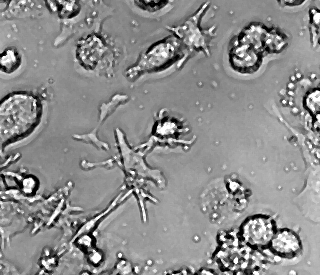

Supplement: Supplementary file 17 — Figure EV2 Source Data [file 44318_2025_515_MOESM17_ESM.zip › FigureEV2/2G/Noc-270min.tif]

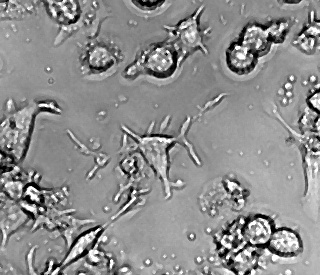

Supplement: Supplementary file 17 — Figure EV2 Source Data [file 44318_2025_515_MOESM17_ESM.zip › FigureEV2/2G/Noc-360min.tif]

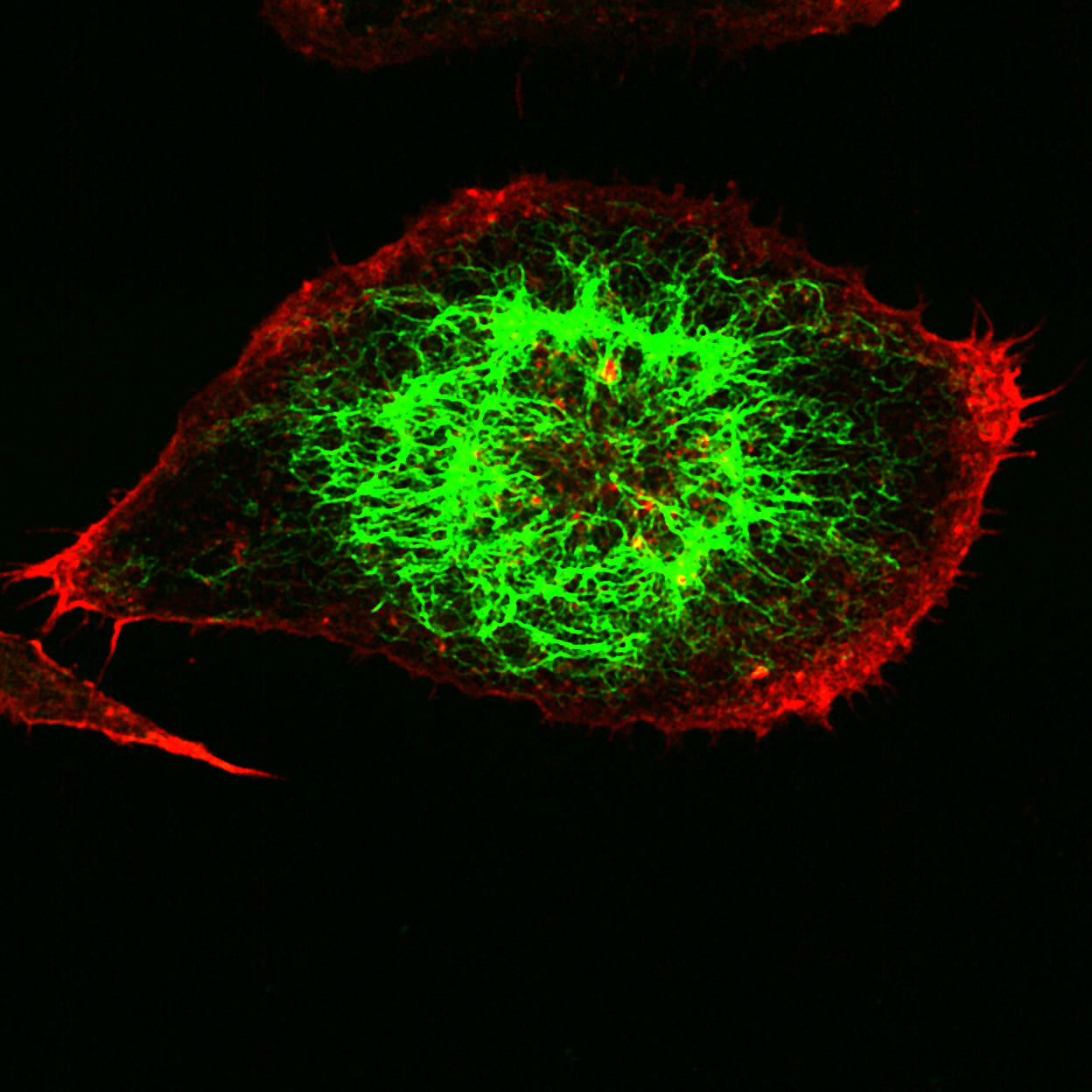

Supplement: Supplementary file 17 — Figure EV2 Source Data [file 44318_2025_515_MOESM17_ESM.zip › FigureEV2/2I/Ctrl.tif]

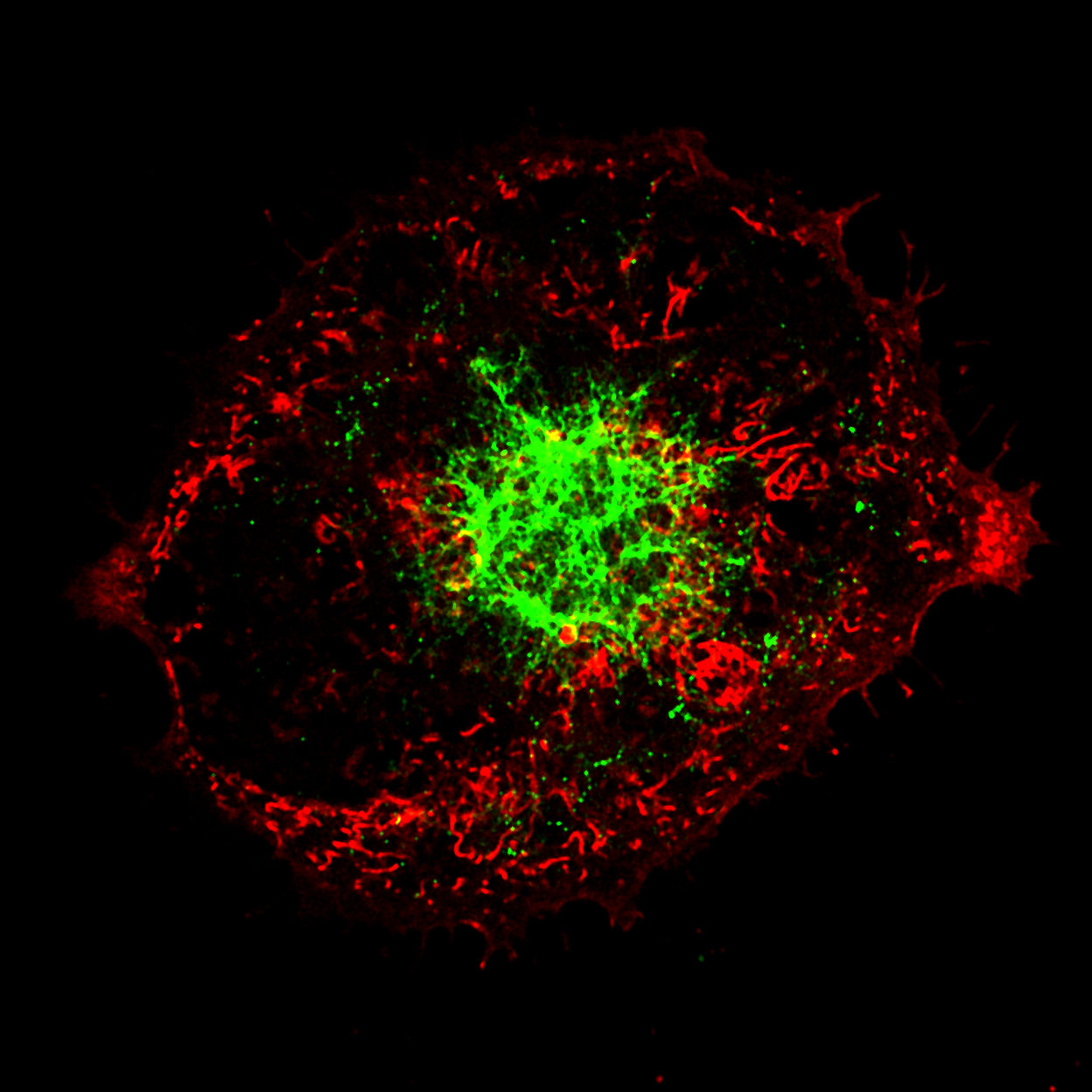

Supplement: Supplementary file 17 — Figure EV2 Source Data [file 44318_2025_515_MOESM17_ESM.zip › FigureEV2/2I/Sim.tif]

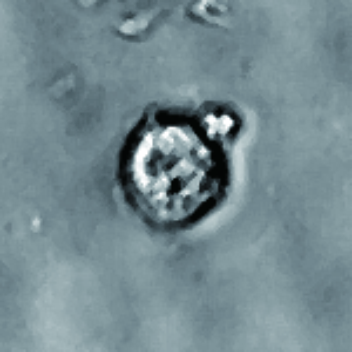

Supplement: Supplementary file 17 — Figure EV2 Source Data [file 44318_2025_515_MOESM17_ESM.zip › FigureEV2/2K/Sim 0min.tif]

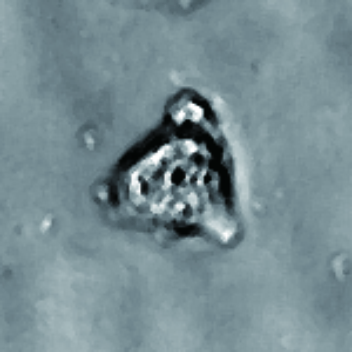

Supplement: Supplementary file 17 — Figure EV2 Source Data [file 44318_2025_515_MOESM17_ESM.zip › FigureEV2/2K/Sim 107min.tif]

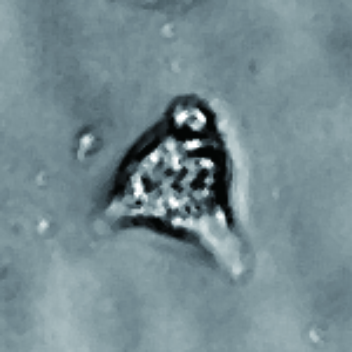

Supplement: Supplementary file 17 — Figure EV2 Source Data [file 44318_2025_515_MOESM17_ESM.zip › FigureEV2/2K/Sim 135min.tif]

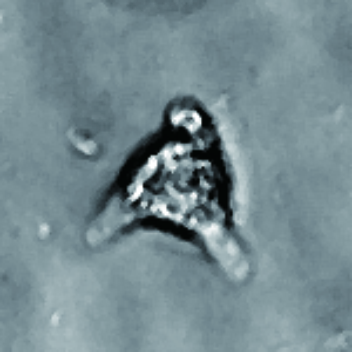

Supplement: Supplementary file 17 — Figure EV2 Source Data [file 44318_2025_515_MOESM17_ESM.zip › FigureEV2/2K/Sim 155min.tif]

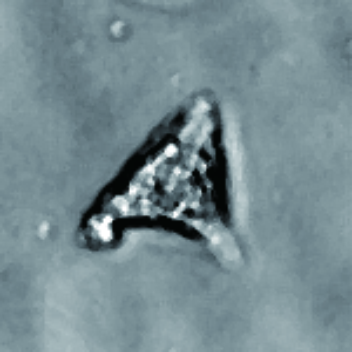

Supplement: Supplementary file 17 — Figure EV2 Source Data [file 44318_2025_515_MOESM17_ESM.zip › FigureEV2/2K/Sim 220min.tif]

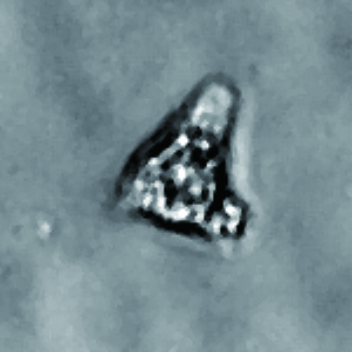

Supplement: Supplementary file 17 — Figure EV2 Source Data [file 44318_2025_515_MOESM17_ESM.zip › FigureEV2/2K/Sim 300min.tif]

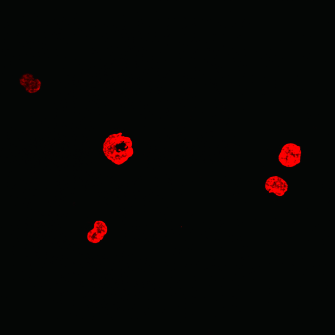

Supplement: Supplementary file 18 — Figure EV3 Source Data [file 44318_2025_515_MOESM18_ESM.zip › FigureEV3/3A/LPS DAPI.tif]

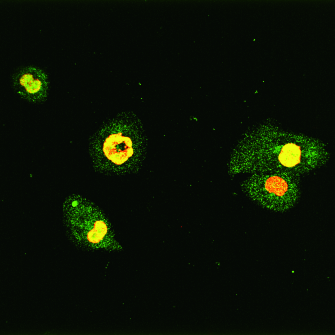

Supplement: Supplementary file 18 — Figure EV3 Source Data [file 44318_2025_515_MOESM18_ESM.zip › FigureEV3/3A/LPS Merge.tif]

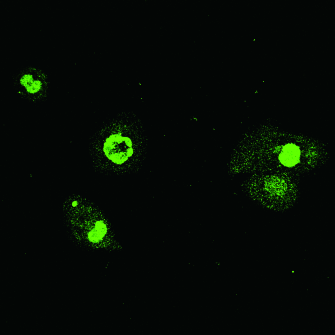

Supplement: Supplementary file 18 — Figure EV3 Source Data [file 44318_2025_515_MOESM18_ESM.zip › FigureEV3/3A/LPS P65.tif]

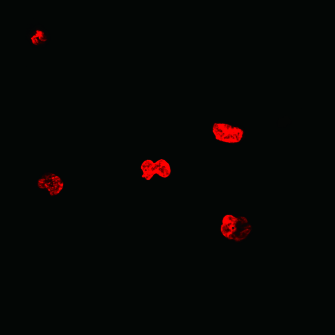

Supplement: Supplementary file 18 — Figure EV3 Source Data [file 44318_2025_515_MOESM18_ESM.zip › FigureEV3/3A/MOCK DAPI.tif]

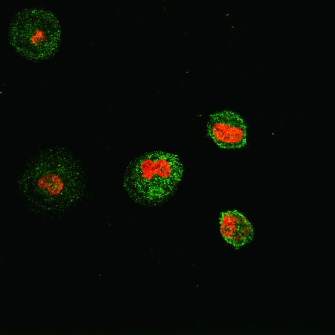

Supplement: Supplementary file 18 — Figure EV3 Source Data [file 44318_2025_515_MOESM18_ESM.zip › FigureEV3/3A/MOCK Merge.tif]

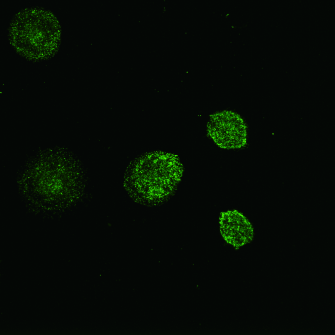

Supplement: Supplementary file 18 — Figure EV3 Source Data [file 44318_2025_515_MOESM18_ESM.zip › FigureEV3/3A/MOCK P65.tif]

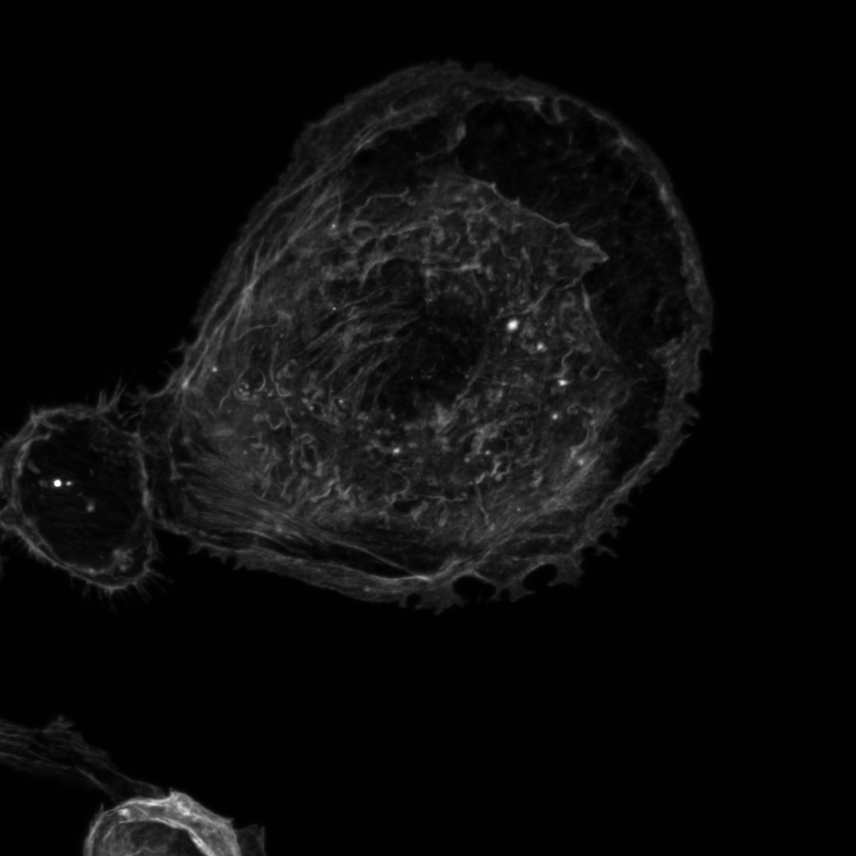

Supplement: Supplementary file 19 — Figure EV4 Source Data [file 44318_2025_515_MOESM19_ESM.zip › FigureEV4/4C-D/THP-1 shapeI Actin CD14.tif]

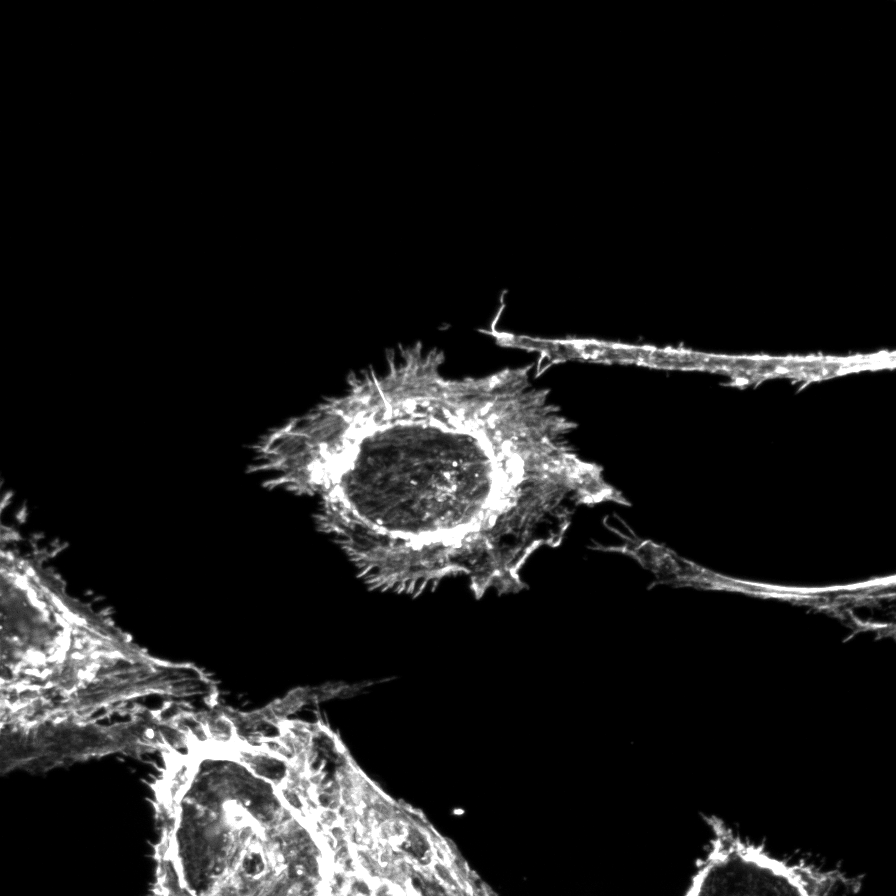

Supplement: Supplementary file 19 — Figure EV4 Source Data [file 44318_2025_515_MOESM19_ESM.zip › FigureEV4/4C-D/THP-1 shapeI Actin CD32.tif]

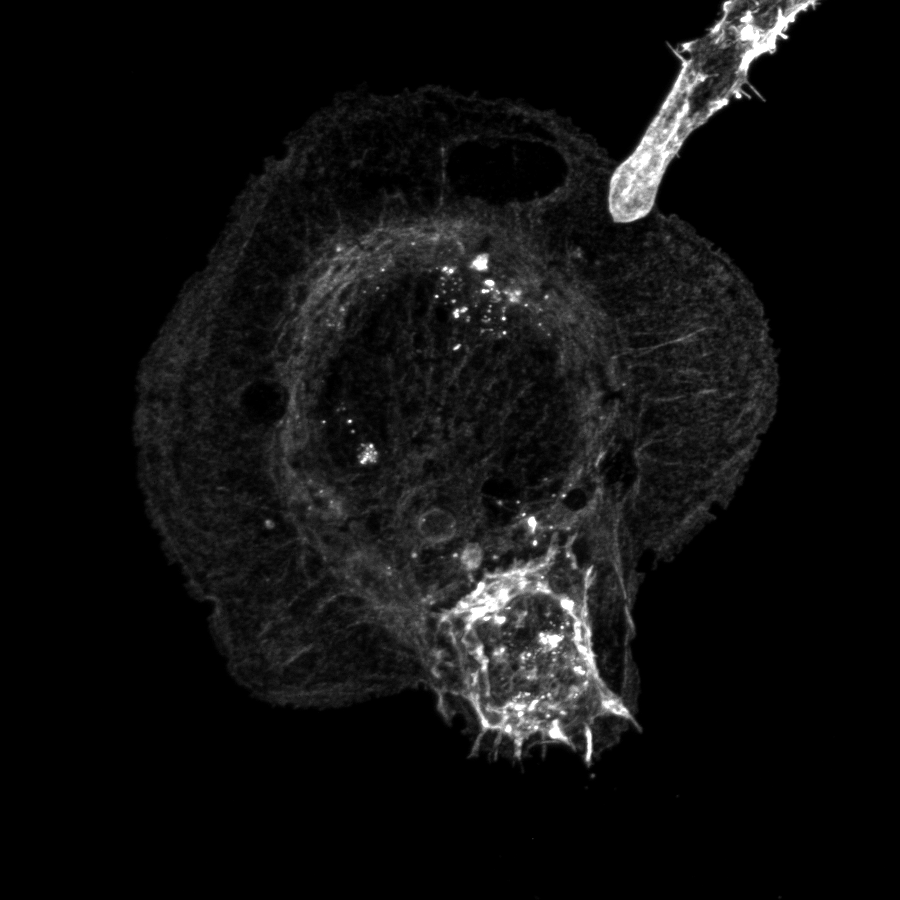

Supplement: Supplementary file 19 — Figure EV4 Source Data [file 44318_2025_515_MOESM19_ESM.zip › FigureEV4/4C-D/THP-1 shapeI Actin CD64.tif]

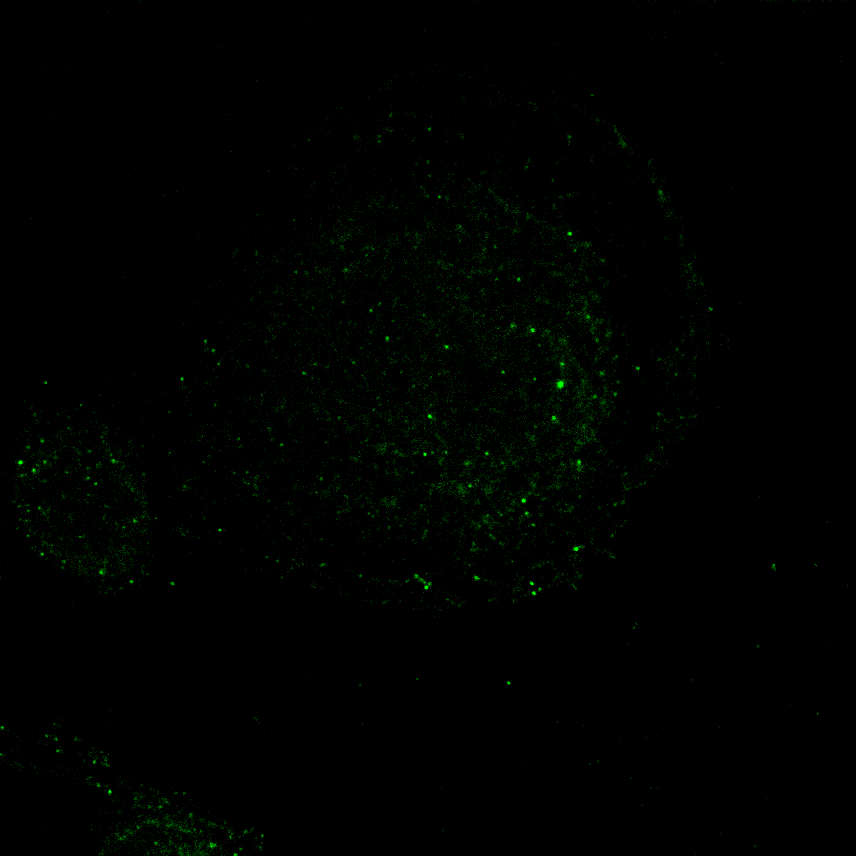

Supplement: Supplementary file 19 — Figure EV4 Source Data [file 44318_2025_515_MOESM19_ESM.zip › FigureEV4/4C-D/THP-1 shapeI CD14.tif]

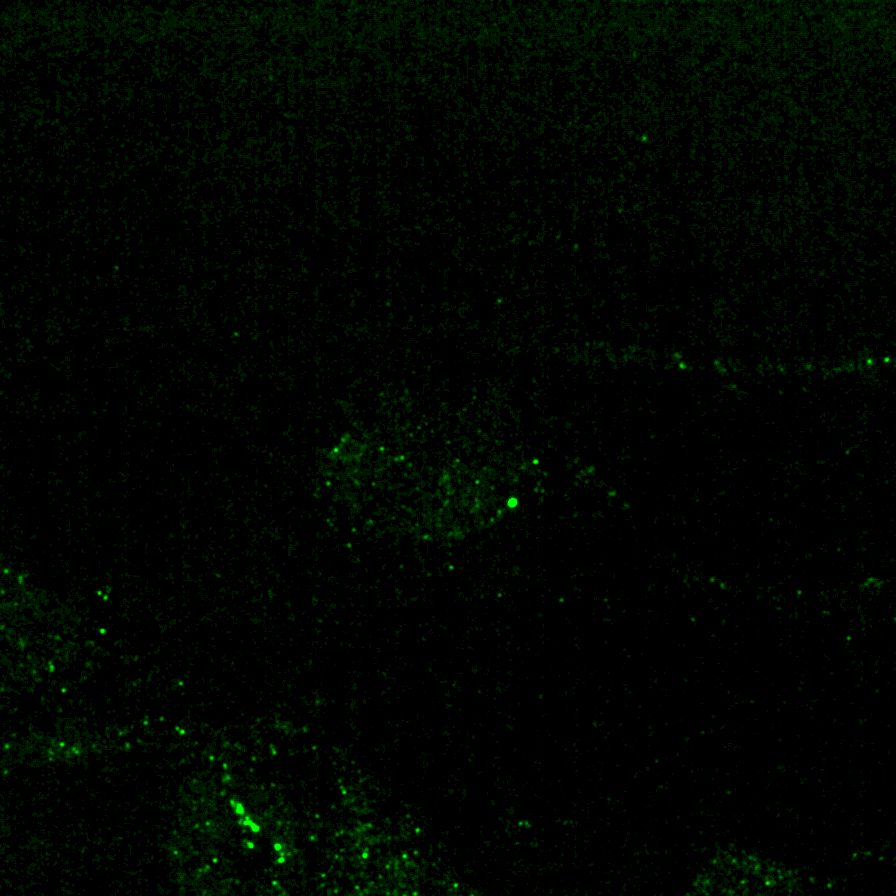

Supplement: Supplementary file 19 — Figure EV4 Source Data [file 44318_2025_515_MOESM19_ESM.zip › FigureEV4/4C-D/THP-1 shapeI CD32.tif]

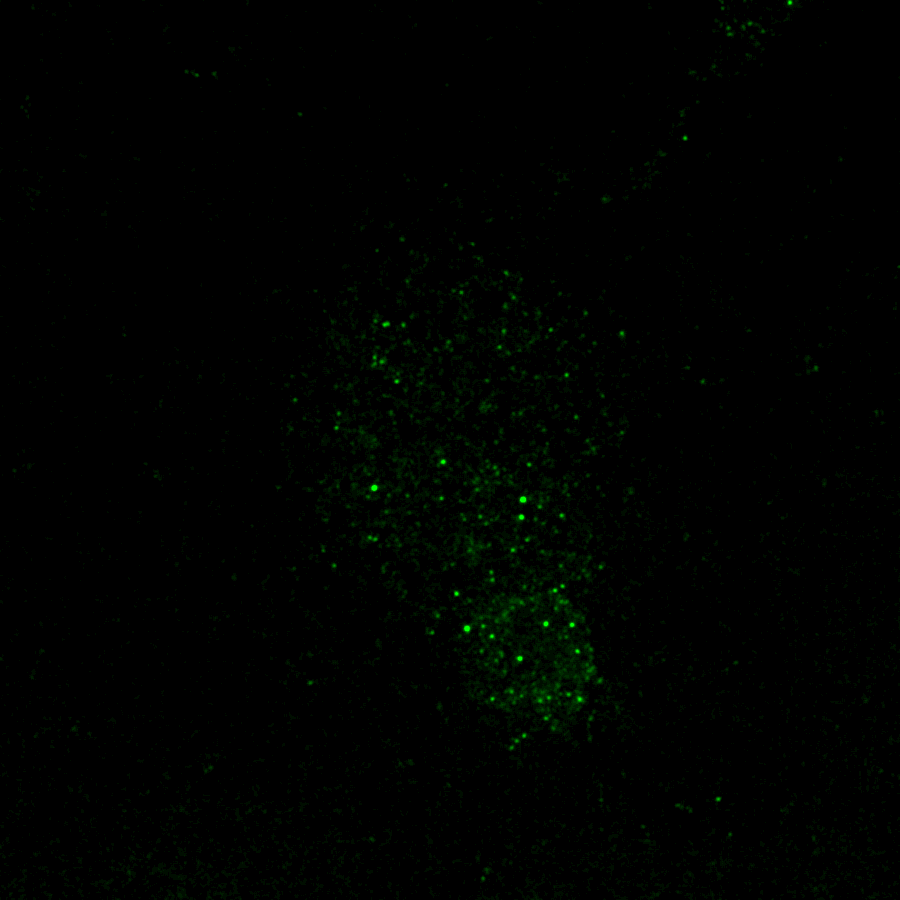

Supplement: Supplementary file 19 — Figure EV4 Source Data [file 44318_2025_515_MOESM19_ESM.zip › FigureEV4/4C-D/THP-1 shapeI CD64.tif]

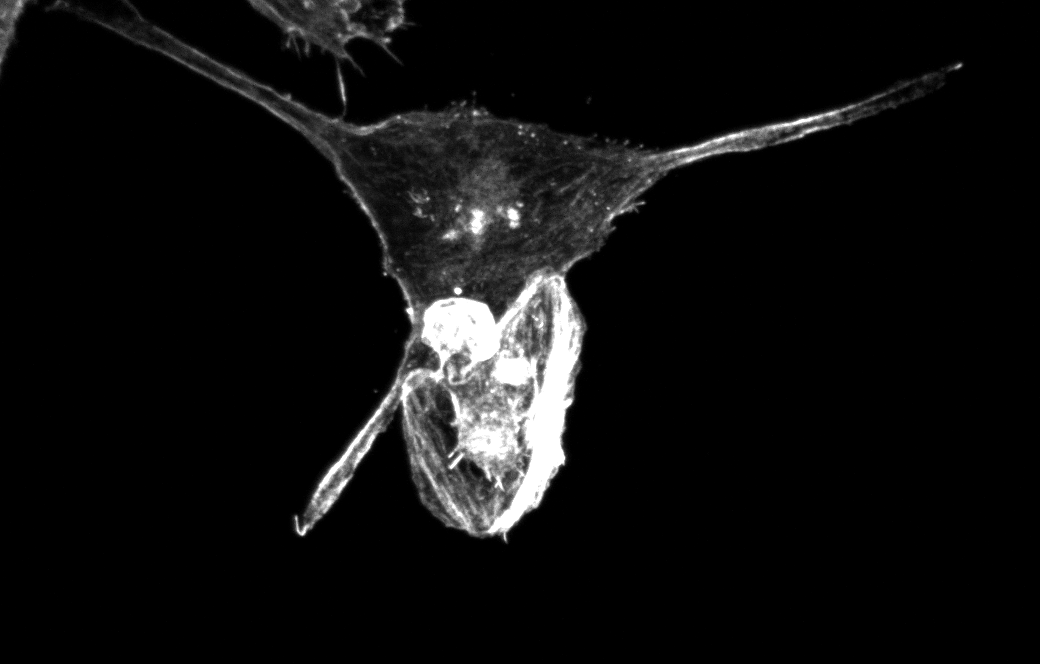

Supplement: Supplementary file 19 — Figure EV4 Source Data [file 44318_2025_515_MOESM19_ESM.zip › FigureEV4/4C-D/THP-1 shapeIII Actin CD14.tif]

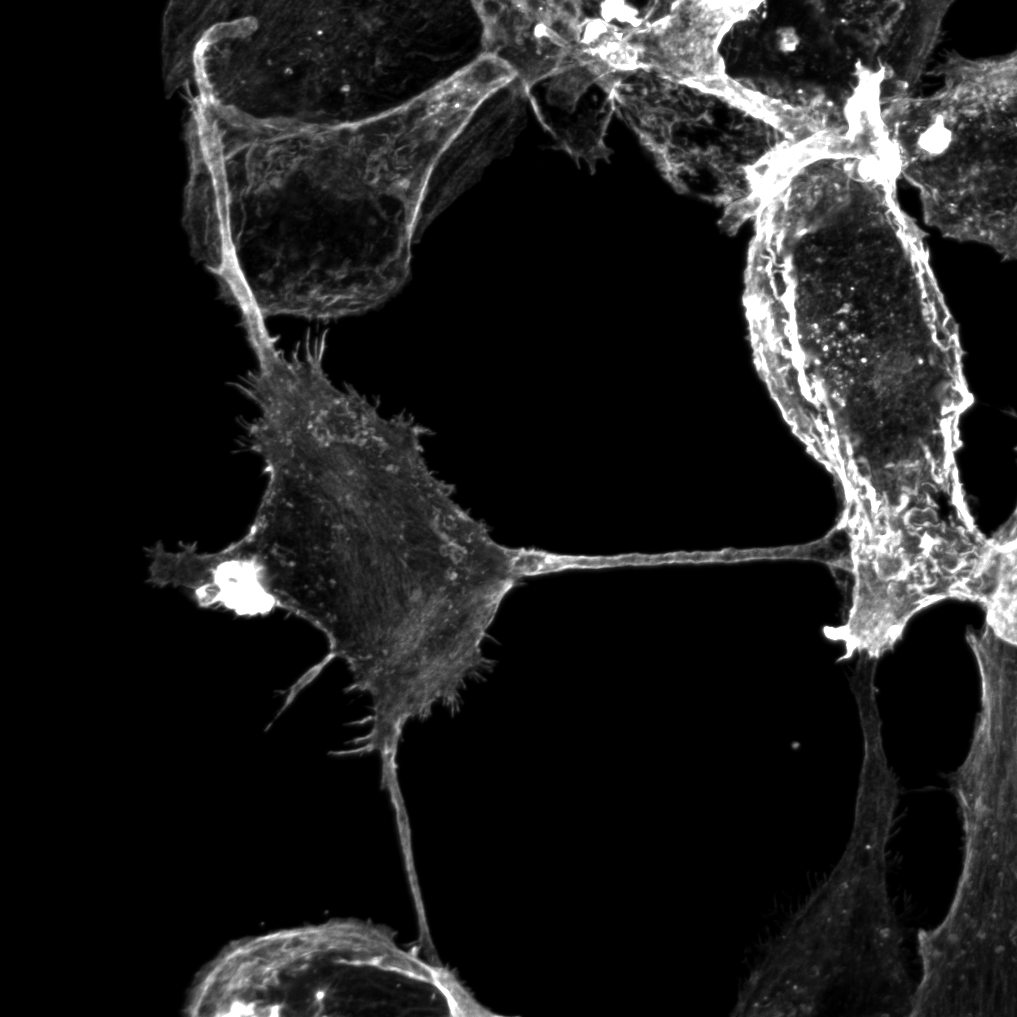

Supplement: Supplementary file 19 — Figure EV4 Source Data [file 44318_2025_515_MOESM19_ESM.zip › FigureEV4/4C-D/THP-1 shapeIII Actin CD32.tif]

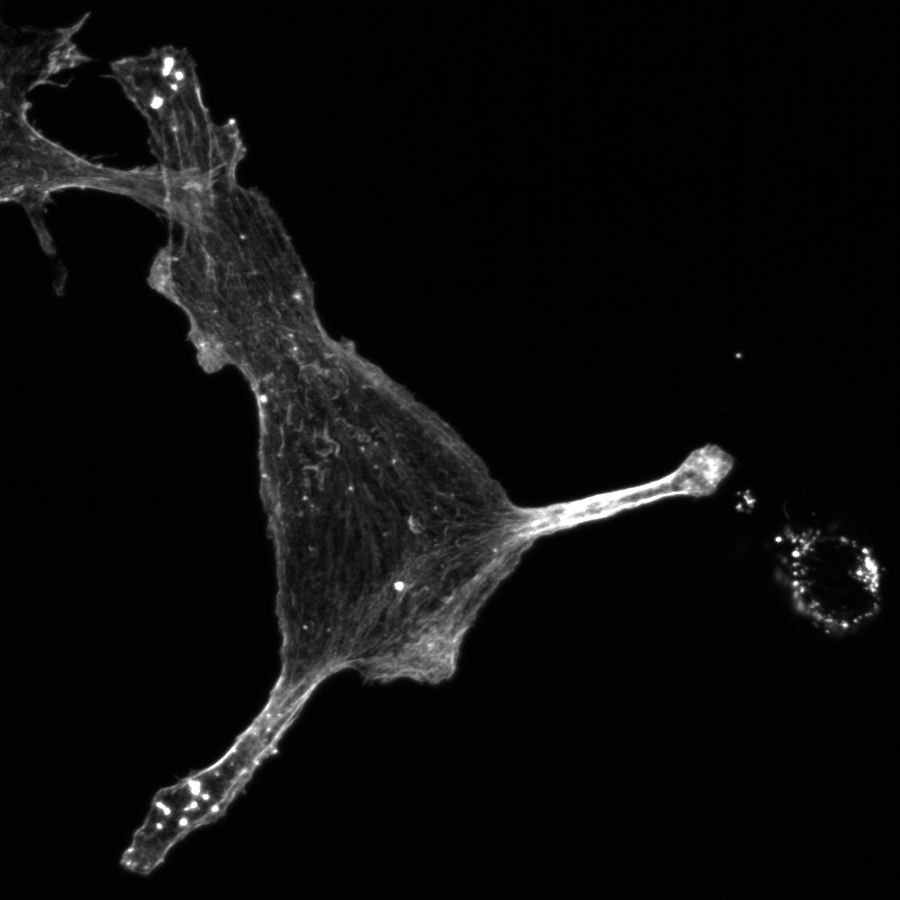

Supplement: Supplementary file 19 — Figure EV4 Source Data [file 44318_2025_515_MOESM19_ESM.zip › FigureEV4/4C-D/THP-1 shapeIII Actin CD64.tif]

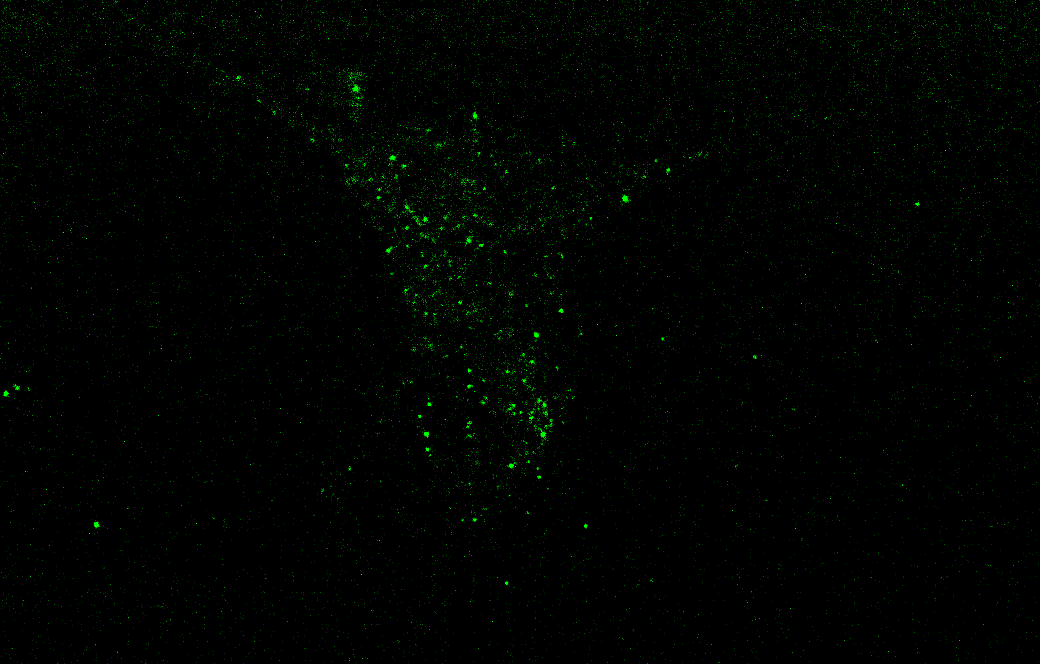

Supplement: Supplementary file 19 — Figure EV4 Source Data [file 44318_2025_515_MOESM19_ESM.zip › FigureEV4/4C-D/THP-1 shapeIII CD14.tif]

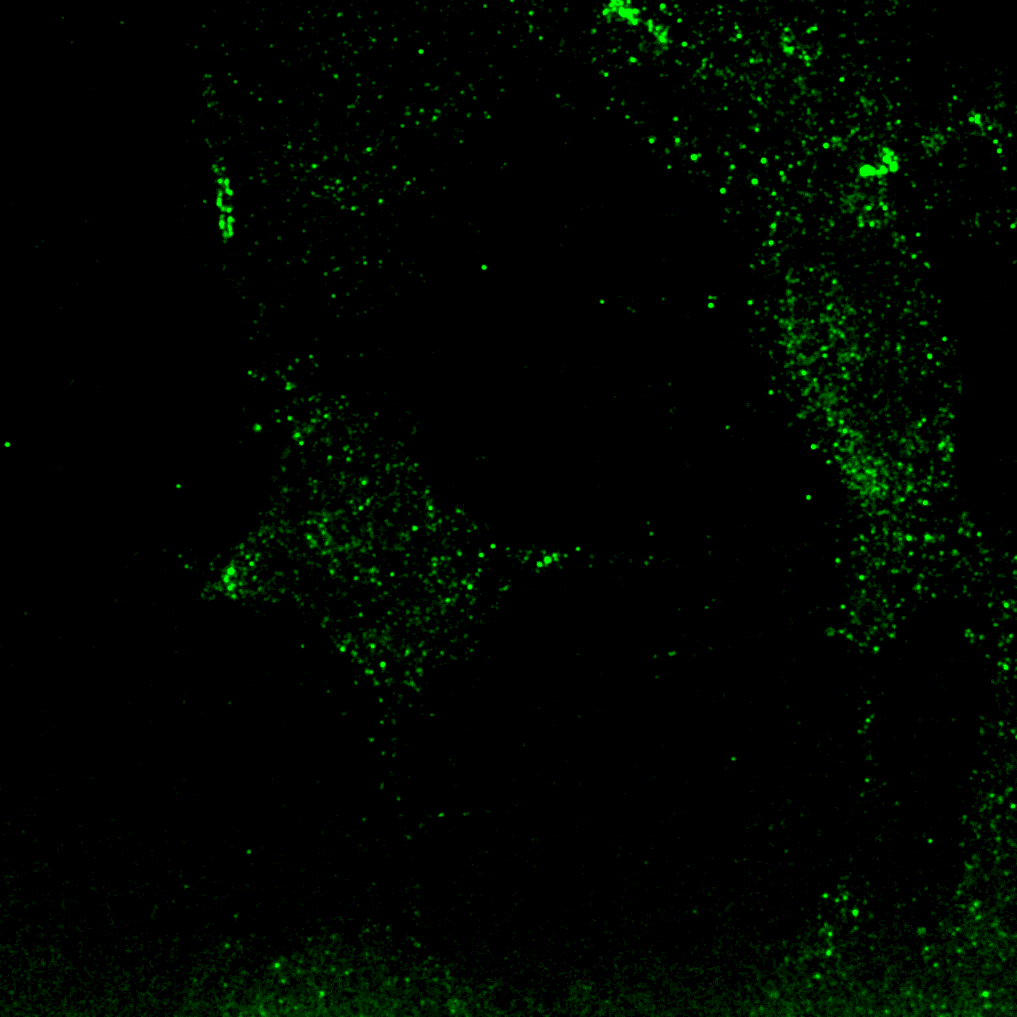

Supplement: Supplementary file 19 — Figure EV4 Source Data [file 44318_2025_515_MOESM19_ESM.zip › FigureEV4/4C-D/THP-1 shapeIII CD32.tif]

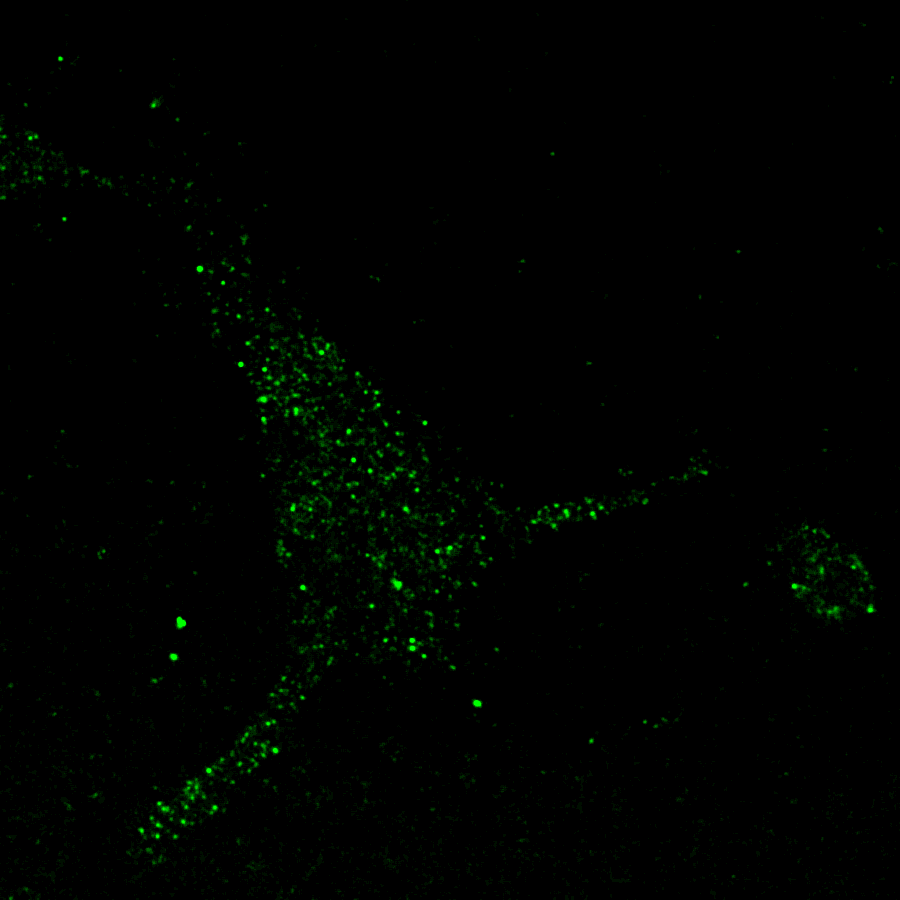

Supplement: Supplementary file 19 — Figure EV4 Source Data [file 44318_2025_515_MOESM19_ESM.zip › FigureEV4/4C-D/THP-1 shapeIII CD64.tif]

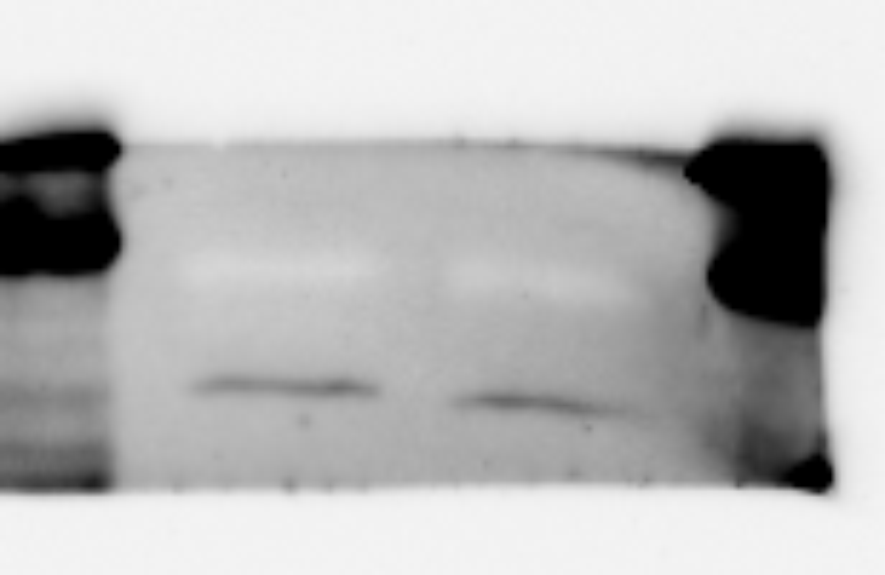

Supplement: Supplementary file 19 — Figure EV4 Source Data [file 44318_2025_515_MOESM19_ESM.zip › FigureEV4/4I/WB ARHGEF3.tif]

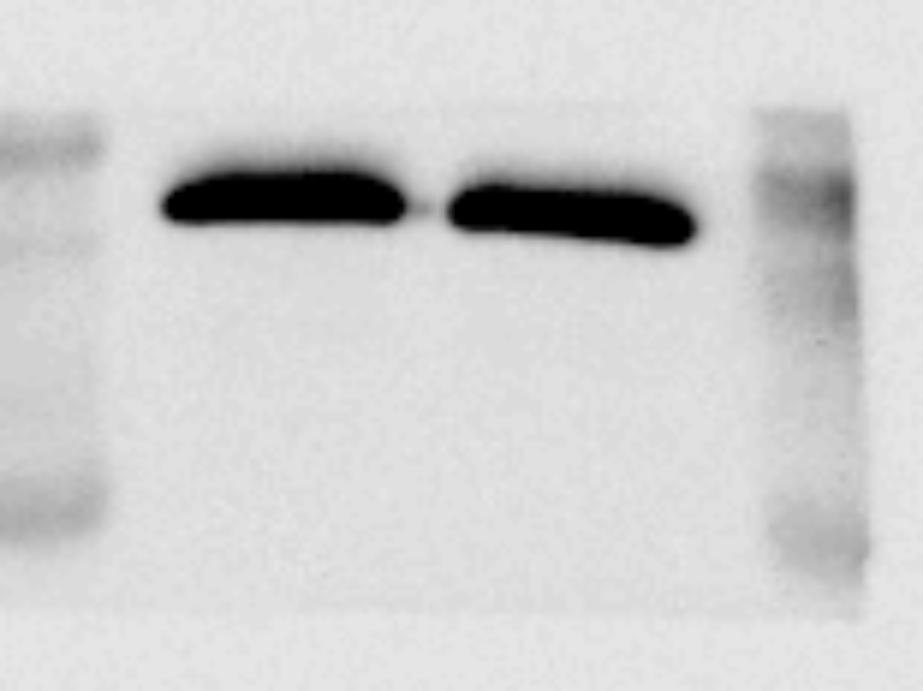

Supplement: Supplementary file 19 — Figure EV4 Source Data [file 44318_2025_515_MOESM19_ESM.zip › FigureEV4/4I/WB GAPDH.tif]

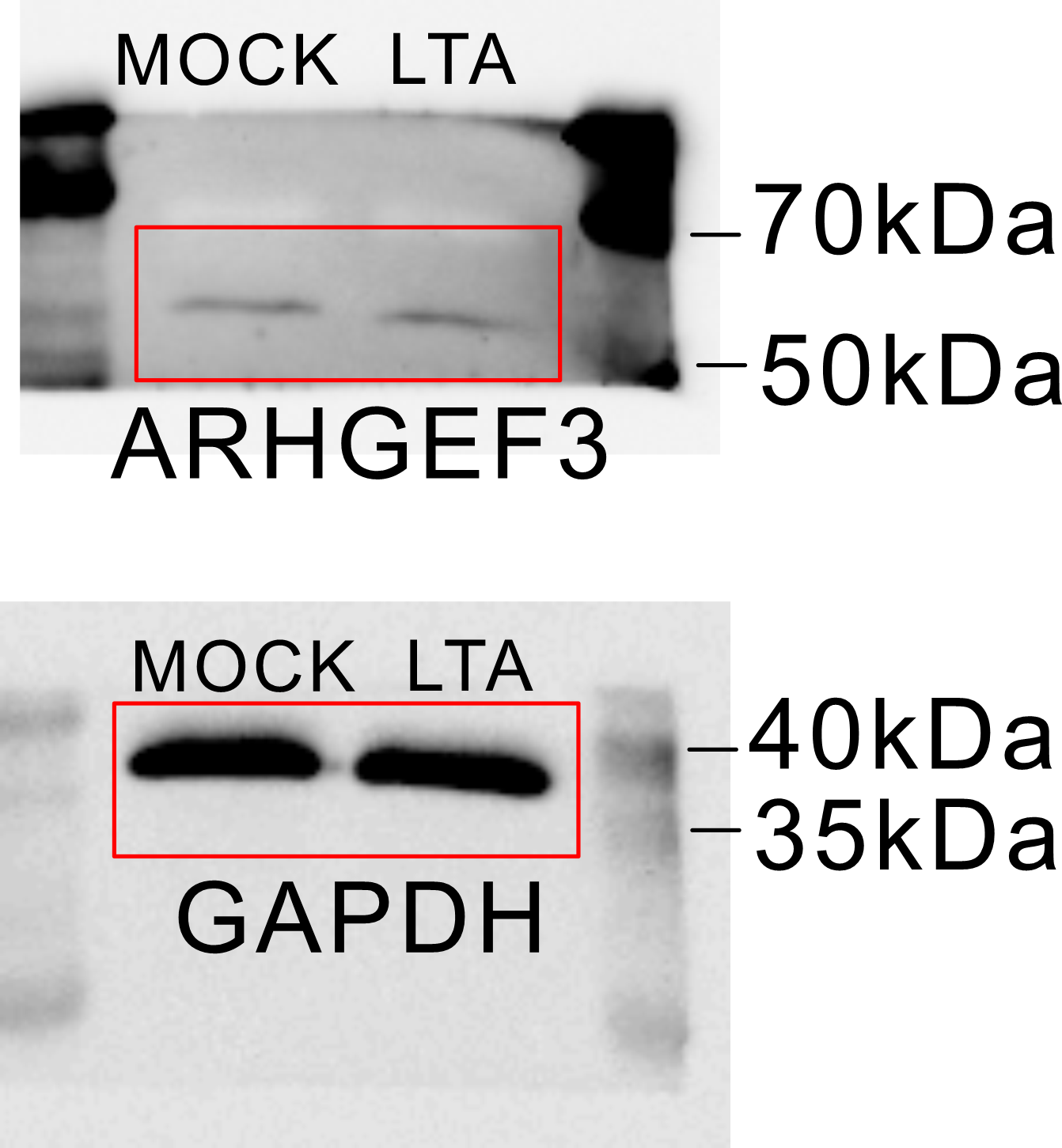

Supplement: Supplementary file 19 — Figure EV4 Source Data [file 44318_2025_515_MOESM19_ESM.zip › FigureEV4/4I/WB source.tif]

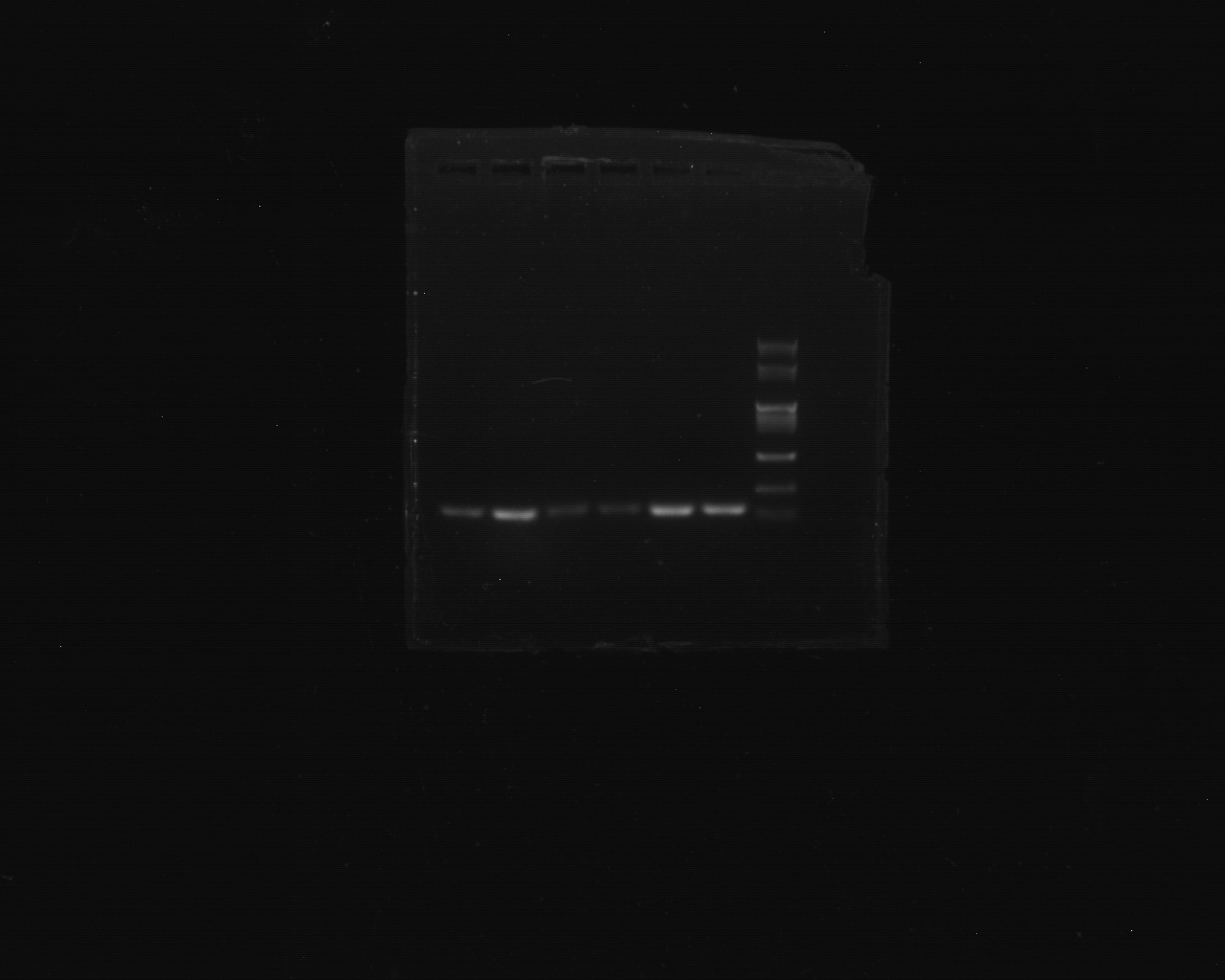

Supplement: Supplementary file 19 — Figure EV4 Source Data [file 44318_2025_515_MOESM19_ESM.zip › FigureEV4/4K/CHIP gel RAW data.bmp]

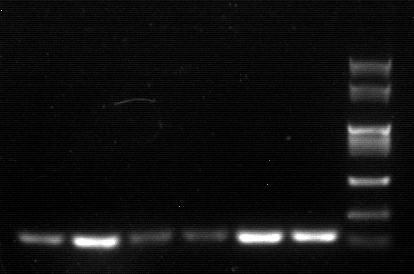

Supplement: Supplementary file 19 — Figure EV4 Source Data [file 44318_2025_515_MOESM19_ESM.zip › FigureEV4/4K/CHIP gel.tif]

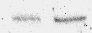

Supplement: Supplementary file 19 — Figure EV4 Source Data [file 44318_2025_515_MOESM19_ESM.zip › FigureEV4/4N-O/PDTC WB ARHGEF3.tif]

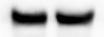

Supplement: Supplementary file 19 — Figure EV4 Source Data [file 44318_2025_515_MOESM19_ESM.zip › FigureEV4/4N-O/PDTC WB GAPDH.tif]

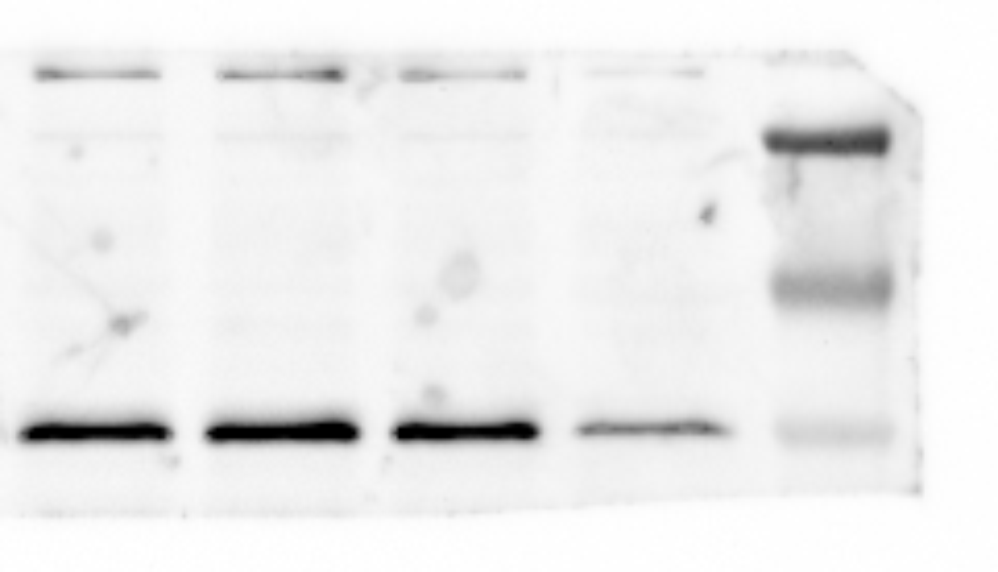

Supplement: Supplementary file 19 — Figure EV4 Source Data [file 44318_2025_515_MOESM19_ESM.zip › FigureEV4/4N-O/WB ARHGEF3.tif]

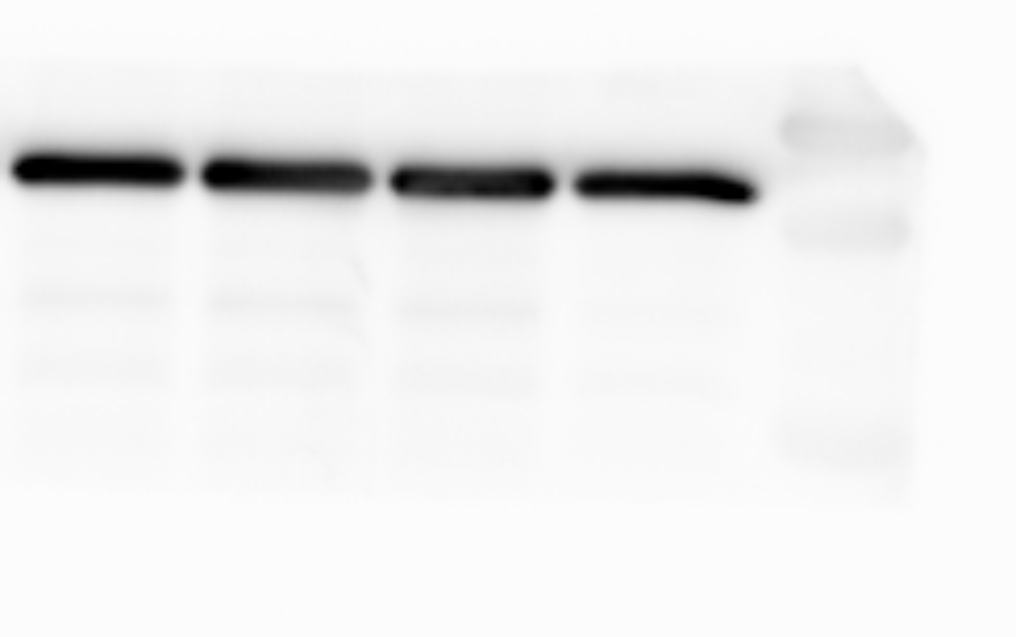

Supplement: Supplementary file 19 — Figure EV4 Source Data [file 44318_2025_515_MOESM19_ESM.zip › FigureEV4/4N-O/WB GAPDH.tif]

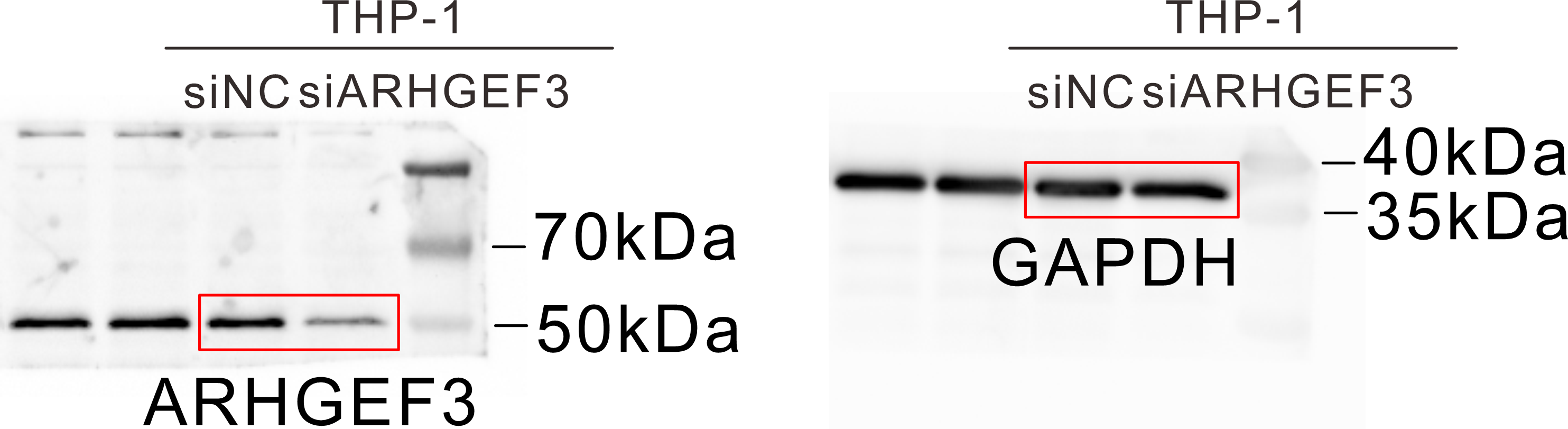

Supplement: Supplementary file 19 — Figure EV4 Source Data [file 44318_2025_515_MOESM19_ESM.zip › FigureEV4/4N-O/WB source.tif]

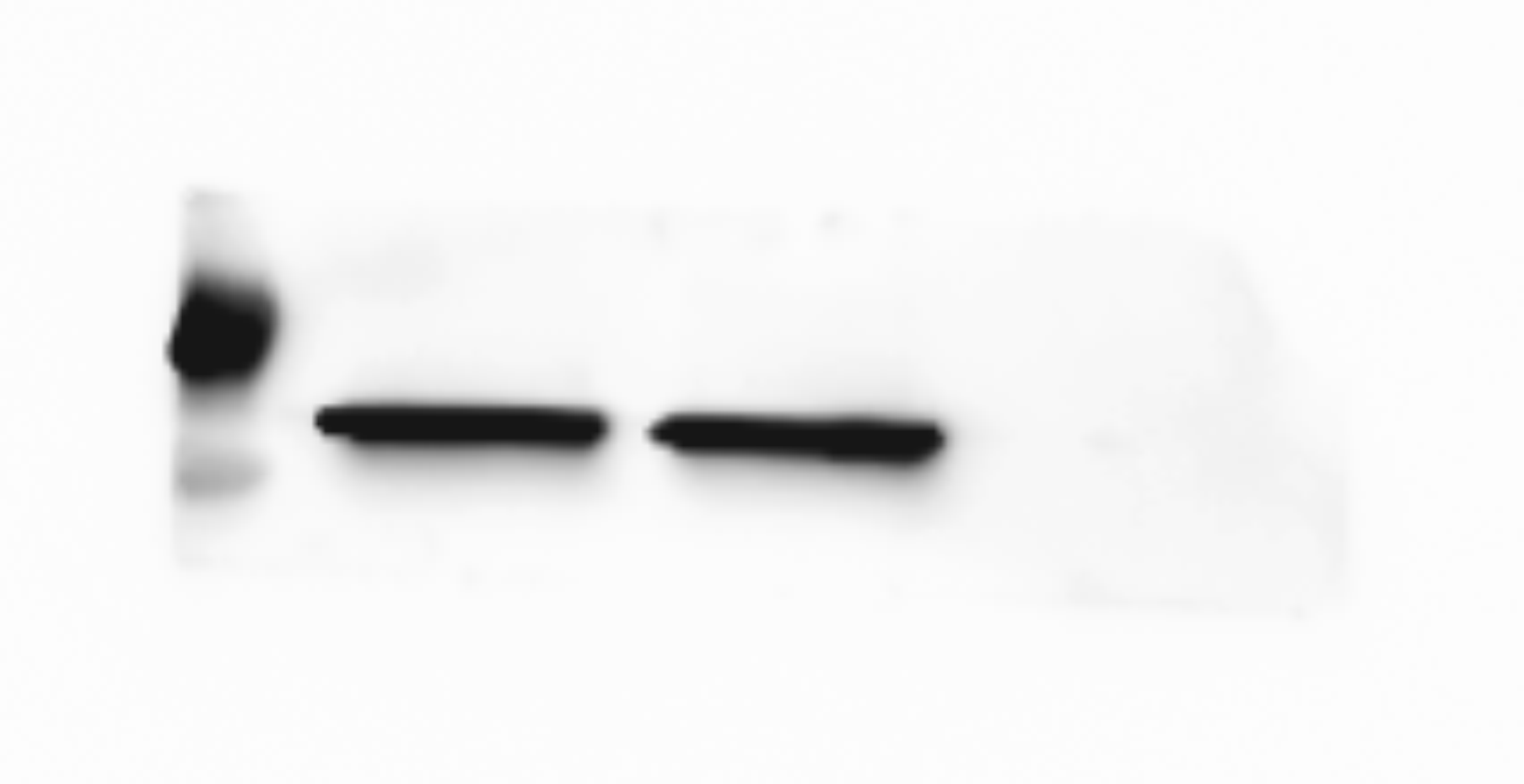

Supplement: Supplementary file 19 — Figure EV4 Source Data [file 44318_2025_515_MOESM19_ESM.zip › FigureEV4/4Q/GAPDH.tif]

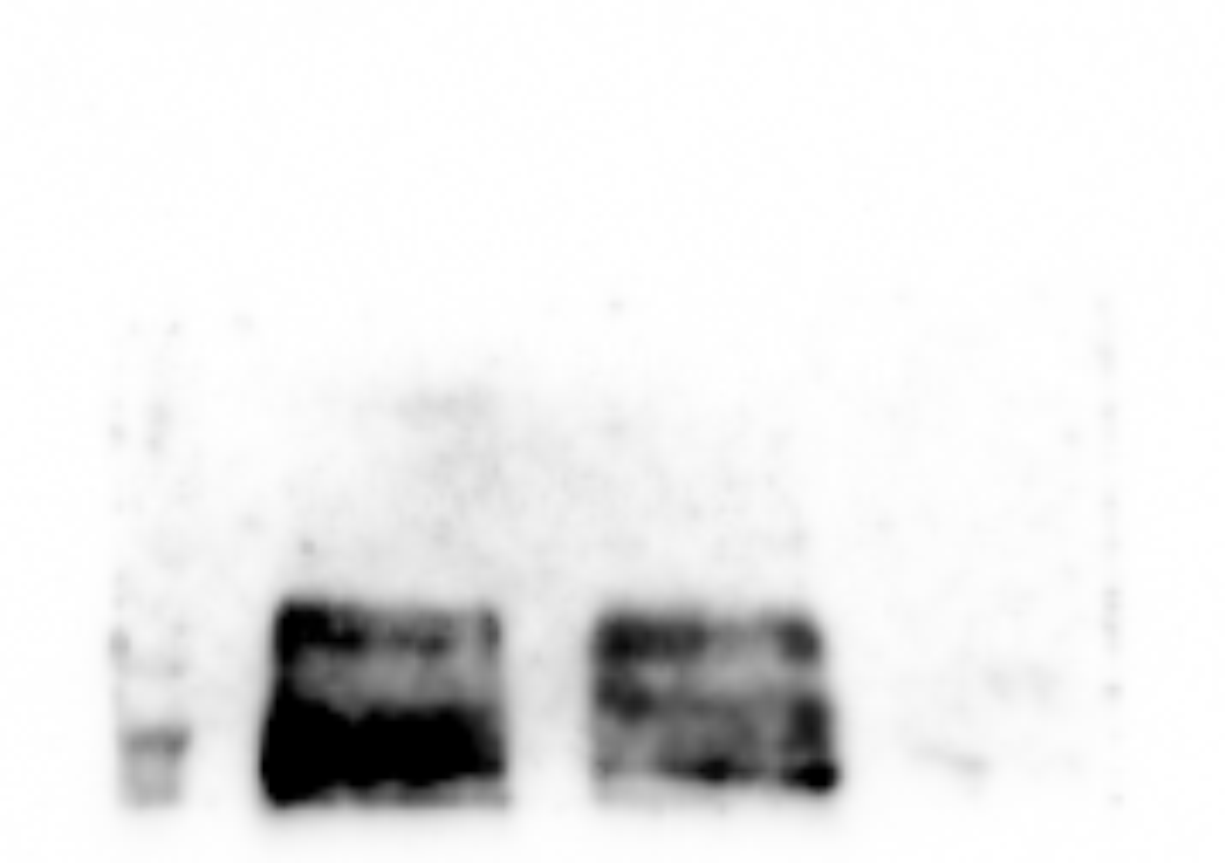

Supplement: Supplementary file 19 — Figure EV4 Source Data [file 44318_2025_515_MOESM19_ESM.zip › FigureEV4/4Q/pMLC.tif]

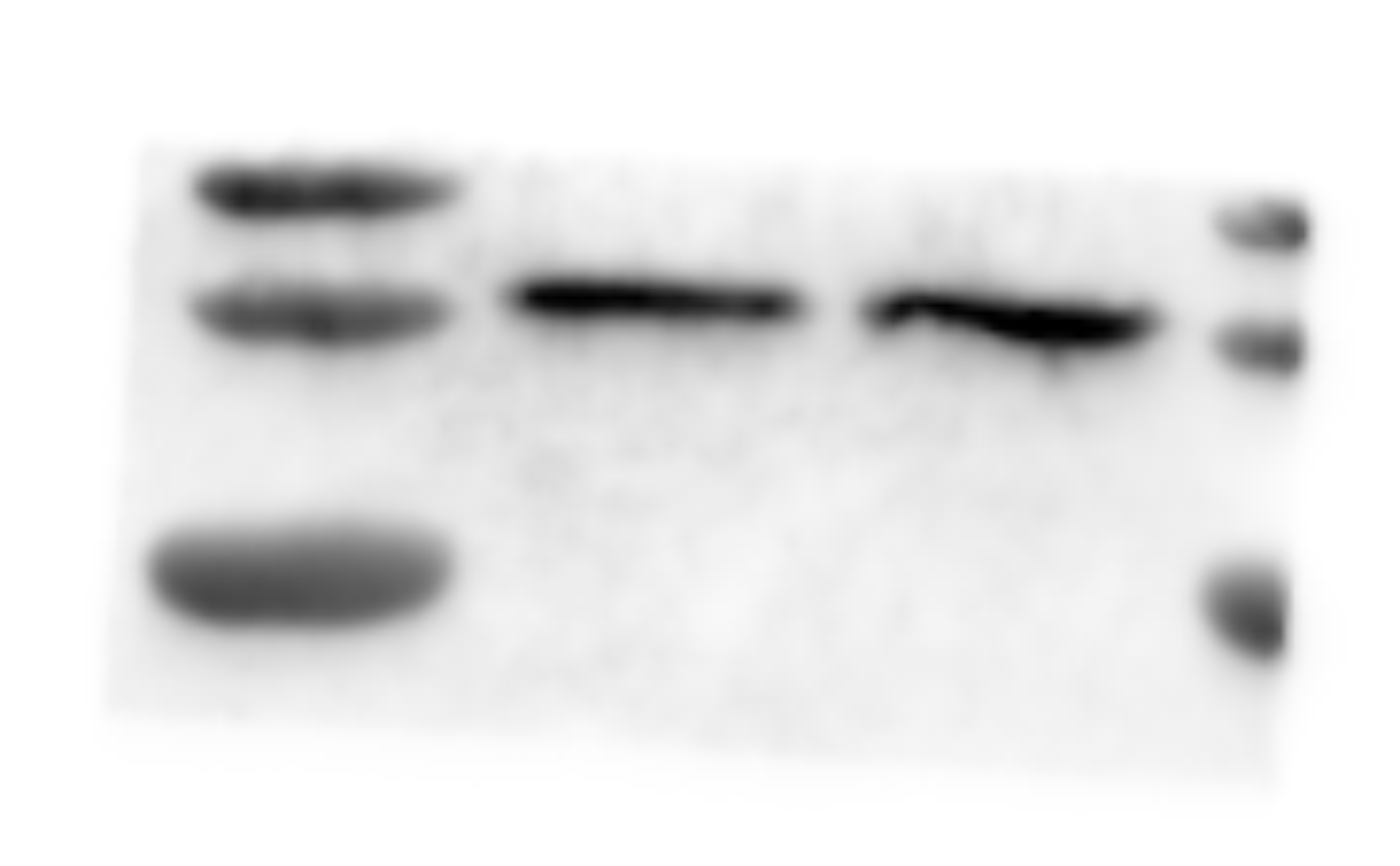

Supplement: Supplementary file 19 — Figure EV4 Source Data [file 44318_2025_515_MOESM19_ESM.zip › FigureEV4/4Q/WB MLC.tif]

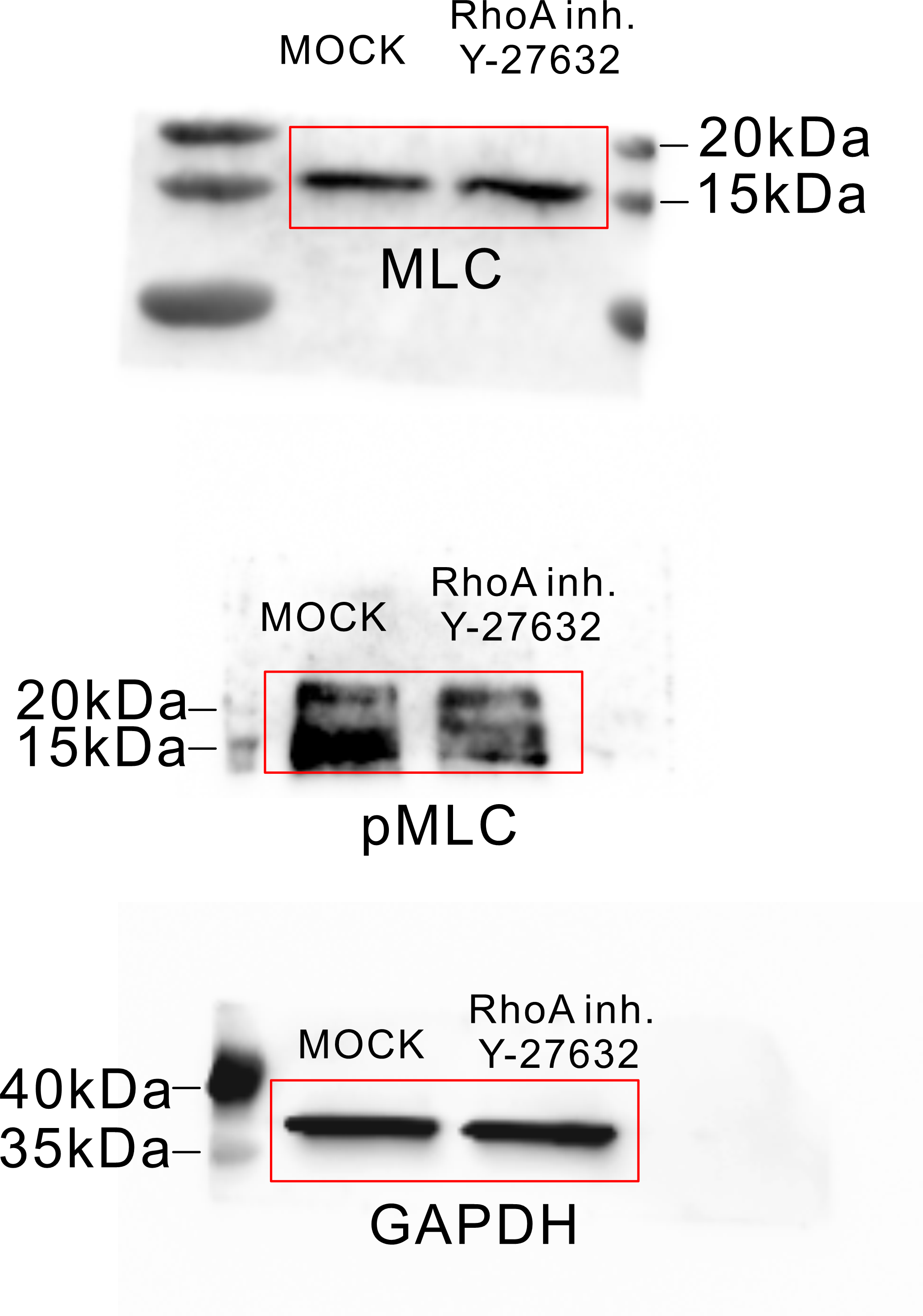

Supplement: Supplementary file 19 — Figure EV4 Source Data [file 44318_2025_515_MOESM19_ESM.zip › FigureEV4/4Q/WB source.tif]

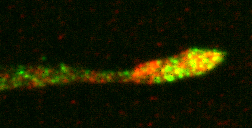

Supplement: Supplementary file 19 — Figure EV4 Source Data [file 44318_2025_515_MOESM19_ESM.zip › FigureEV4/4T-V/iBMDM Macropodia 1 merge.tif]

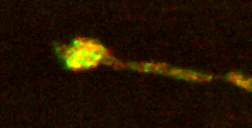

Supplement: Supplementary file 19 — Figure EV4 Source Data [file 44318_2025_515_MOESM19_ESM.zip › FigureEV4/4T-V/iBMDM Macropodia 2 merge.tif]

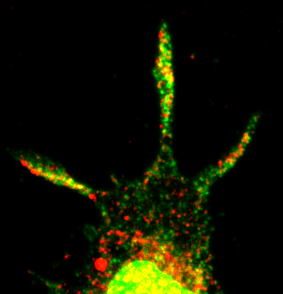

Supplement: Supplementary file 19 — Figure EV4 Source Data [file 44318_2025_515_MOESM19_ESM.zip › FigureEV4/4T-V/Profile THP-1 macropodia.tif]

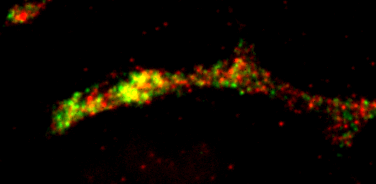

Supplement: Supplementary file 19 — Figure EV4 Source Data [file 44318_2025_515_MOESM19_ESM.zip › FigureEV4/4T-V/RAW264.7 Macropodia 1 merge.tif]

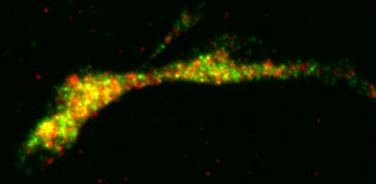

Supplement: Supplementary file 19 — Figure EV4 Source Data [file 44318_2025_515_MOESM19_ESM.zip › FigureEV4/4T-V/RAW264.7 Macropodia 2 merge.tif]

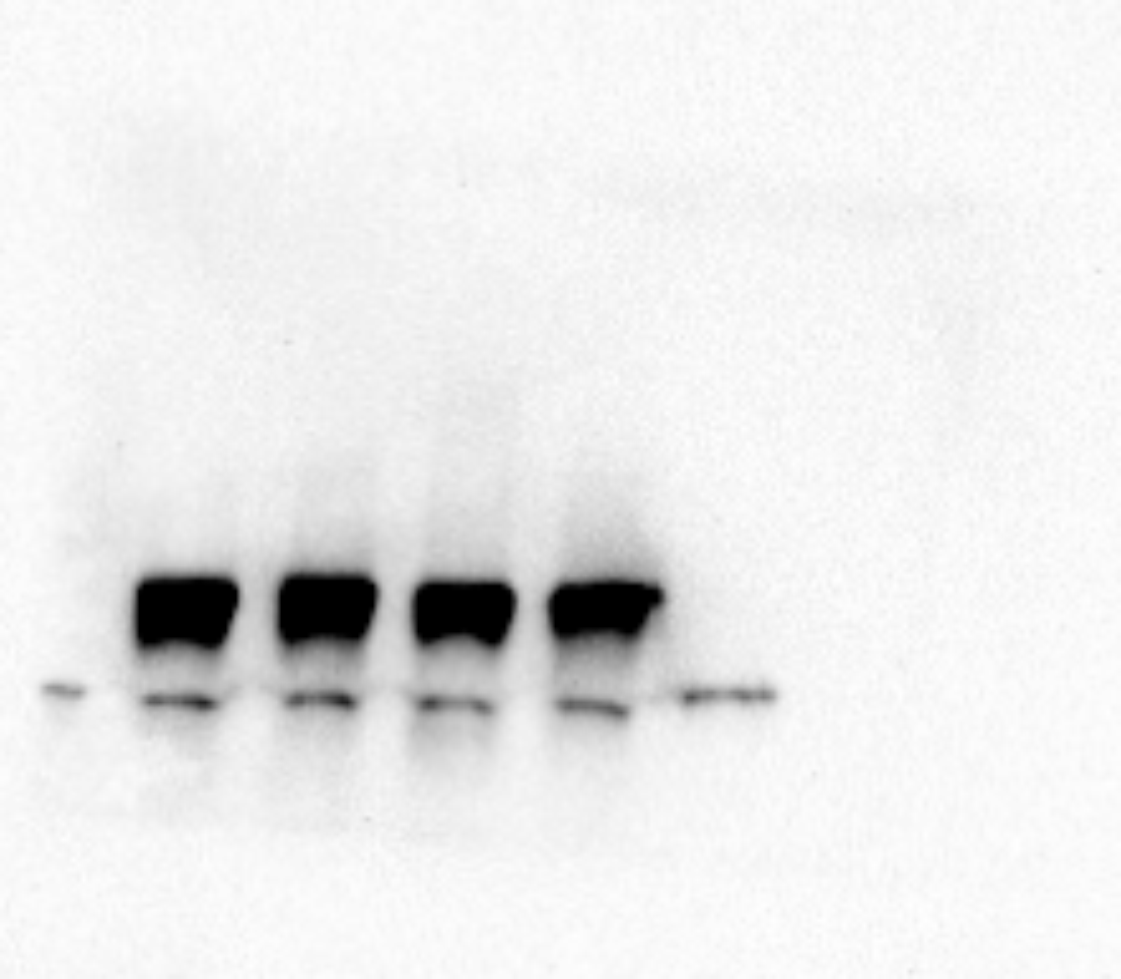

Supplement: Supplementary file 20 — Figure EV5 Source Data [file 44318_2025_515_MOESM20_ESM.zip › FigureEV5/5G/WB GAPDH.tif]

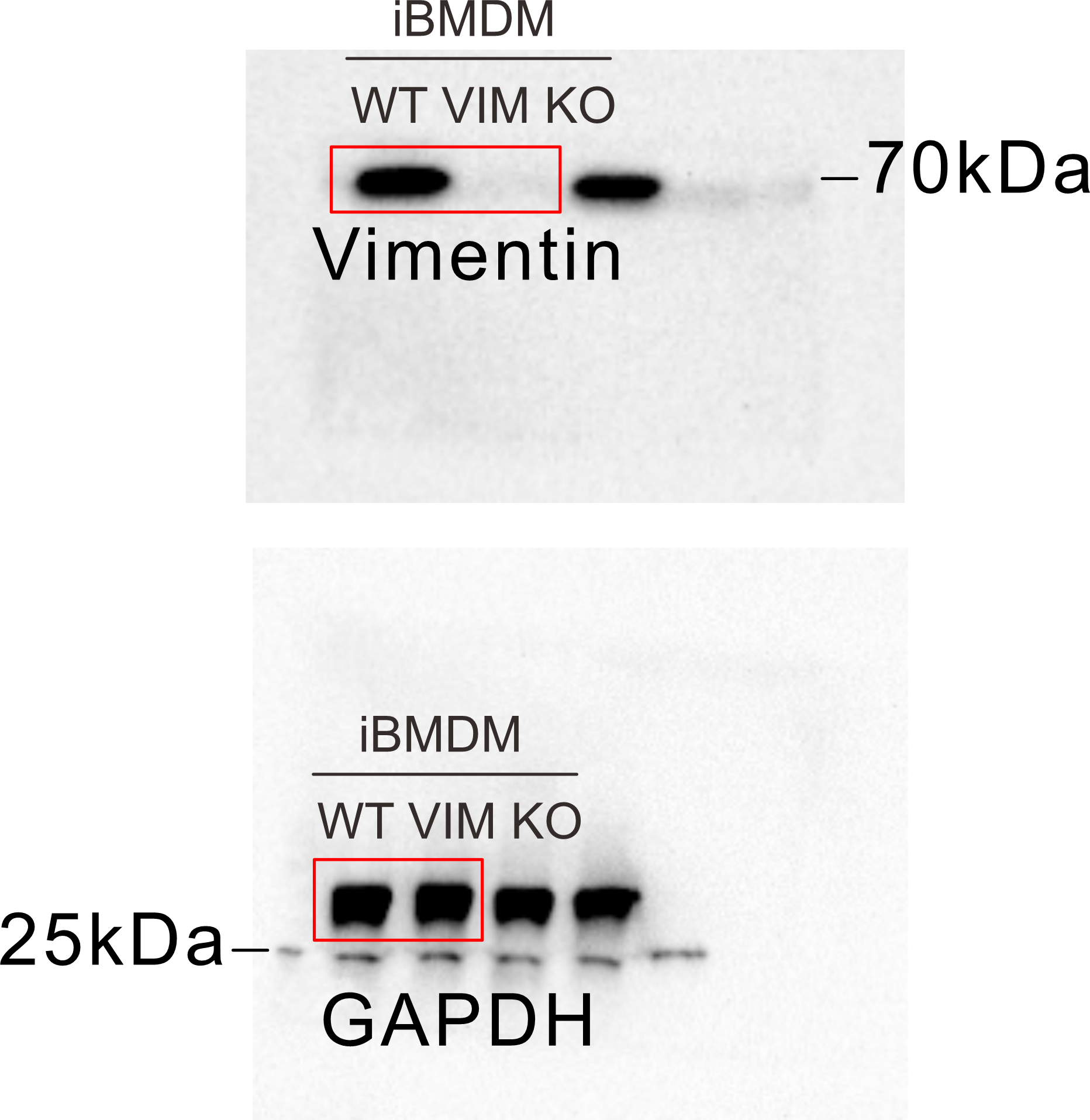

Supplement: Supplementary file 20 — Figure EV5 Source Data [file 44318_2025_515_MOESM20_ESM.zip › FigureEV5/5G/WB source.tif]

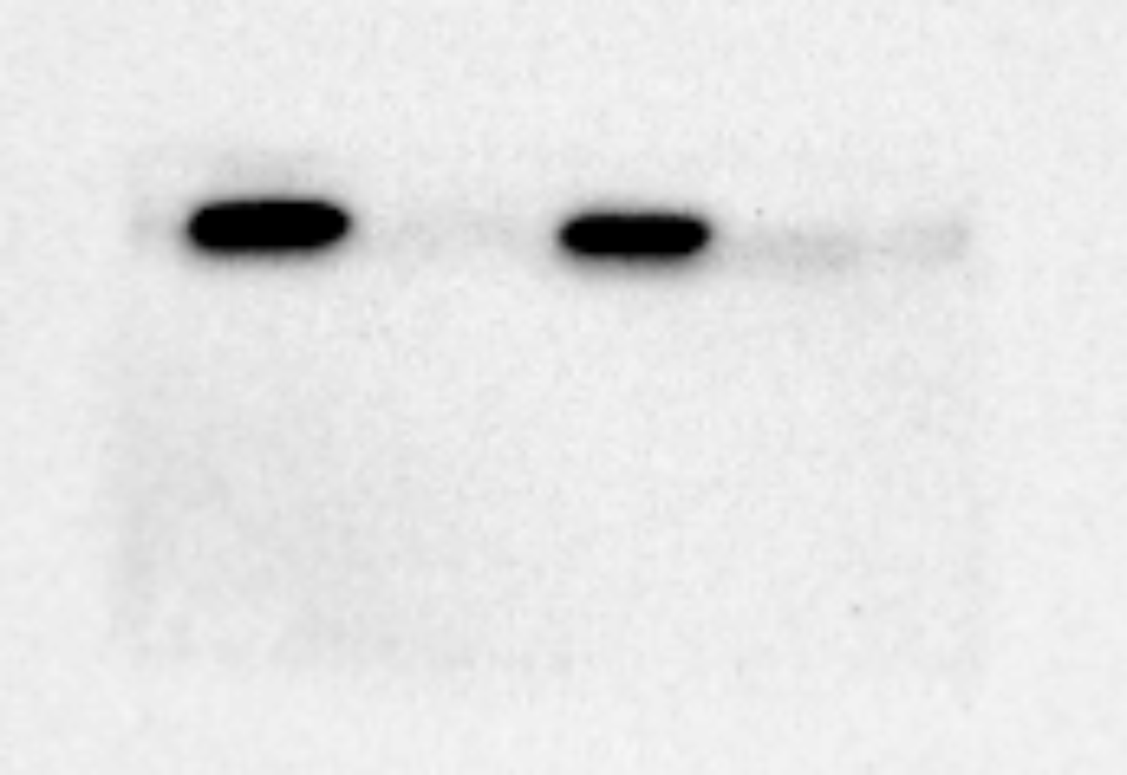

Supplement: Supplementary file 20 — Figure EV5 Source Data [file 44318_2025_515_MOESM20_ESM.zip › FigureEV5/5G/WB vimentin.tif]
