# Supplementary material for: Macrophages form dendrite-like pseudopods to enhance bacterial ingestion
Source: EMBO J. 2025 Jul 28;44(17):4772–802. doi: 10.1038/s44318-025-00515-z (PMC12402336; doi:10.1038/s44318-025-00515-z)
Supplement: Supplementary file 21 — Expanded View Figures [file 44318_2025_515_MOESM21_ESM.pdf]

Expanded View Figures

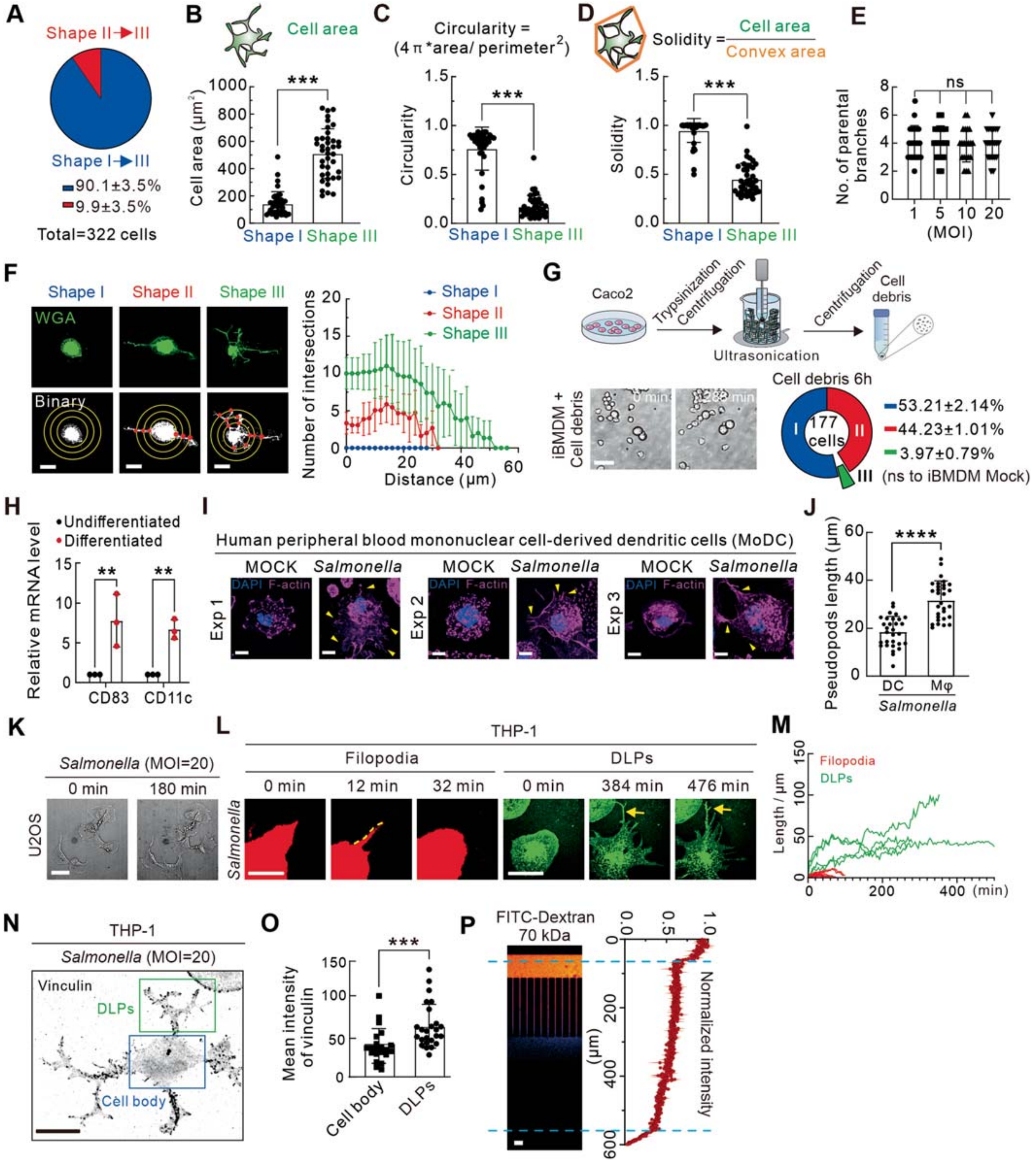

Figure EV1. Macrophages construct dendrite-like pseudopods (DLPs) in response to bacterial infection.

(**A**) Pie chart quantification of the percentage of shape III macrophages derived from shape I/II macrophages with Salmonella infection (MOI = 20). Blue, shape I to shape III; Red, shape II to shape III. The percentages presented in the form of mean ± s.d. (**B-D**) Quantification of cell area (**B**), circularity (**C**) and solidity (**D**) of shape I and shape III macrophages. $n = 50$ cells. (**E**) Quantification of the number of primary pseudopods derived from THP-1 cell body upon *Salmonella* infection with distinct MOIs. (**F**) Representative images visualized by WGA in shape I, shape II and shape III THP-1 macrophages and processed by Binary and Sholl analysis. Scale bar, 20 µm. Right panel shows the quantification of the number of intersections along DLPs based on Sholl analysis. $n = 20$ cells. (**G**) Schematic diagram of cell debris extraction (upper panel) and the time-lapse images of iBMDMs stimulated with cell debris (lower left panel). Scale bar, 50 µm. Pie chart quantification of the percentage of three shapes of macrophages with cell debris stimulation for 6 h (lower right panel). The percentages presented in the form of mean ± s.d. (**H**) Quantifications of the transcriptional levels of dendritic cell markers (CD83 and CD11c) by RT-qPCR. (**I**) Representative images of MoDCs derived from Human peripheral blood mononuclear cell-derived dendritic cells with and without *Salmonella* infection (MOI = 20) for 6 h. Scale bar, 10 µm. (**J**) Quantification of DLPs length in *Salmonella* infected MoDCs and macrophages. $n = 30$ cells. (**K**) Representative time-lapse images of U2OS cells upon *Salmonella* infection (MOI = 20). Scale bar, 10 µm. (**L**) Immunofluorescence of THP-1 macrophages infected with *Salmonella* and stained with WGA. Scale bar, 5 µm (filopodia panel) and 20 µm (DLPs panel). (**M**) Quantification of filopodia and DLPs length upon *Salmonella* infection. (**N**) Invert confocal images visualized by vinculin staining in *Salmonella* infected THP-1 macrophages. Blue and green boxes depicting the region of cell body and DLPs, respectively. Bars, 20 µm. (**O**) Quantification of the mean intensity of vinculin in ROIs of cell body and DLPs in (**N**). $n = 28$ cells. (**P**) Representative fluorescent imaging revealing the diffusion of 0.5 mg/mL 70 kDa FITC-Dextran in the microchannel 6 h after loading. Line profile on the right panel illustrates the fluorescence intensity of FITC-Dextran along the microchannels. Scale bars, 60 µm. Data are presented as mean ± s.d. from three independent experiments. Dots in quantifications presented individual cells. $P = 2.38284E-18$ (**B**). $P = 2.16145E-24$ (**C**). $P = 2.74828E-25$ (**D**). *P* values from left to right (**E**): $P = 0.89574$, $P = 0.60961$, $P = 0.787850183$, $P = 0.51197$, $P = 0.67727$, $P = 0.78709$. *P* values from left to right (**H**): $P = 0.00233$, $P = 0.00128$. $P = 2.07762E-09$ (**J**). $P = 0.00093$ (O). ns $P > 0.05$; $^{**}P < 0.001$; $^{***}P < 0.001$: unpaired two-tailed Student's *t* test (**B–D**, **G**, **J**). one-way/two-way ANOVA with Sidak's analysis (**E**, **H**). Source data are available online for this figure.

 © The Author(s)

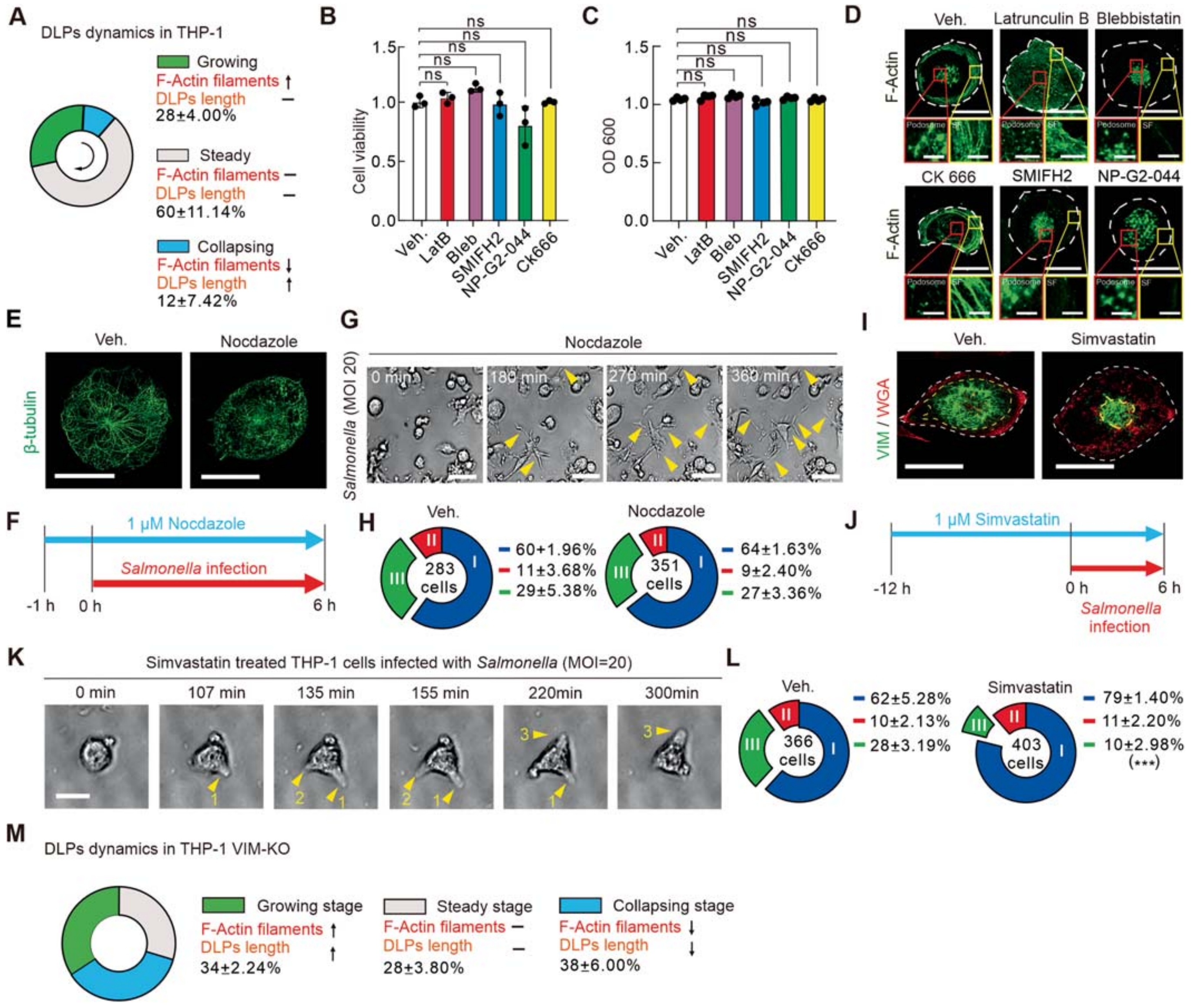

Figure EV2. DLPs formation is dependent on cytoskeleton.

(**A**) Pie chart shows the percentage of time spent in each stage of DLPs in wild-type THP-1 macrophages. (**B**) Quantification of the cell toxicity of the actin inhibitors. (**C**) Quantification of bacterial growth rate following the treatment of the actin inhibitors. (**D**) Representative images of actin filaments visualized by phalloidin in Veh., 0.5 μM Latrunculin B (LatB), 10 μM Blebbistatin, 40 μM CK666, 15 μM SMIFH2 and 1 μM NP-G2-044 treated THP-1 macrophages, respectively. Red and yellow magnified regions represent the podosome and contractile stress fibers (SF), respectively. Scale bars, 10 μm (in cell images) and 5 μm (in the magnified images). White dash lines outline cell contours. (**E**) Effects of 1 μM Nocodazole treatment on THP-1 macrophages for 1 h. The endogenous microtubule network was visualized by β-tubulin antibody staining. Scale bars, 20 μm. (**F**) Diagram of the Nocodazole treatment procedure. (**G**) Time-lapse images of THP-1 macrophages during *Salmonella* infection for 6 h with Nocodazole treatment (MOI = 20). Scale bars, 20 μm. (**H**) Pie charts show the percentage of three shapes in macrophages infected with *salmonella* for 6 h. Veh. $n = 283$ cells; Nocodazole treatment, $n = 351$ cells. (**I**) Representative images of endogenous vimentin in vehicle and 1 μM simvastatin treated cells. White and yellow dashed lines depict the regions of cell outline and vimentin network, respectively. Scale bars, 10 μm. (**J**) Schematic diagram of simvastatin treatment procedure. (**K**) Time-lapse images of THP-1 macrophages during *Salmonella* infection for 6 h with simvastatin treatment (MOI = 20). Scale bars, 20 μm. Yellow arrowheads depict DLPs, while the hollow arrowheads depict the diminishing DLPs. (**L**) Pie charts show the distribution of macrophage shapes following vehicle and simvastatin treatment with *salmonella* infection for 6 h. Veh., $n = 366$ cells; simvastatin treatment, $n = 403$ cells. (**M**) Pie chart shows the percentage of time spent in each stage of DLPs in VIM KO THP-1. Data are presented as mean ± s.d. from three independent experiments. $P = 0.00040$ (**L**). ns $P > 0.05$. ***$P < 0.001$: unpaired two-tailed Student's *t* test (**B**, **C**, **H**, **L**). Source data are available online for this figure.

© The Author(s)

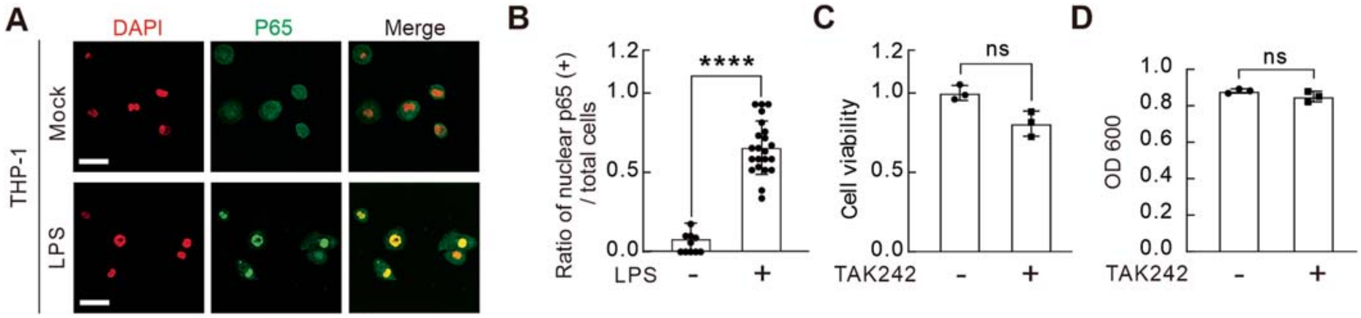

Figure EV3. TLR4 is a key factor for DLPs formation.

(**A**) Representative images show the localization of NF-κB/P65 in THP-1 macrophages under mock or LPS-treated conditions. Scale bar, 20 μm. (**B**) Quantification of the nuclear P65 ratio relative to total cell count in (**A**). (**C**) Quantification of cell toxicity followingTAK-242 treatment. (**D**) Quantification of bacterial growth with TAK-242 treatment. Data are presented as mean ± s.d. from three independent experiments. Dots in quantifications presented individual cells. $P = 2.27158E\text{-}07$ (**B**). ns $P > 0.05$; ****$P < 0.0001$: unpaired two-tailed Student's *t* test (**B**–**D**). Source data are available online for this figure.

 © The Author(s)

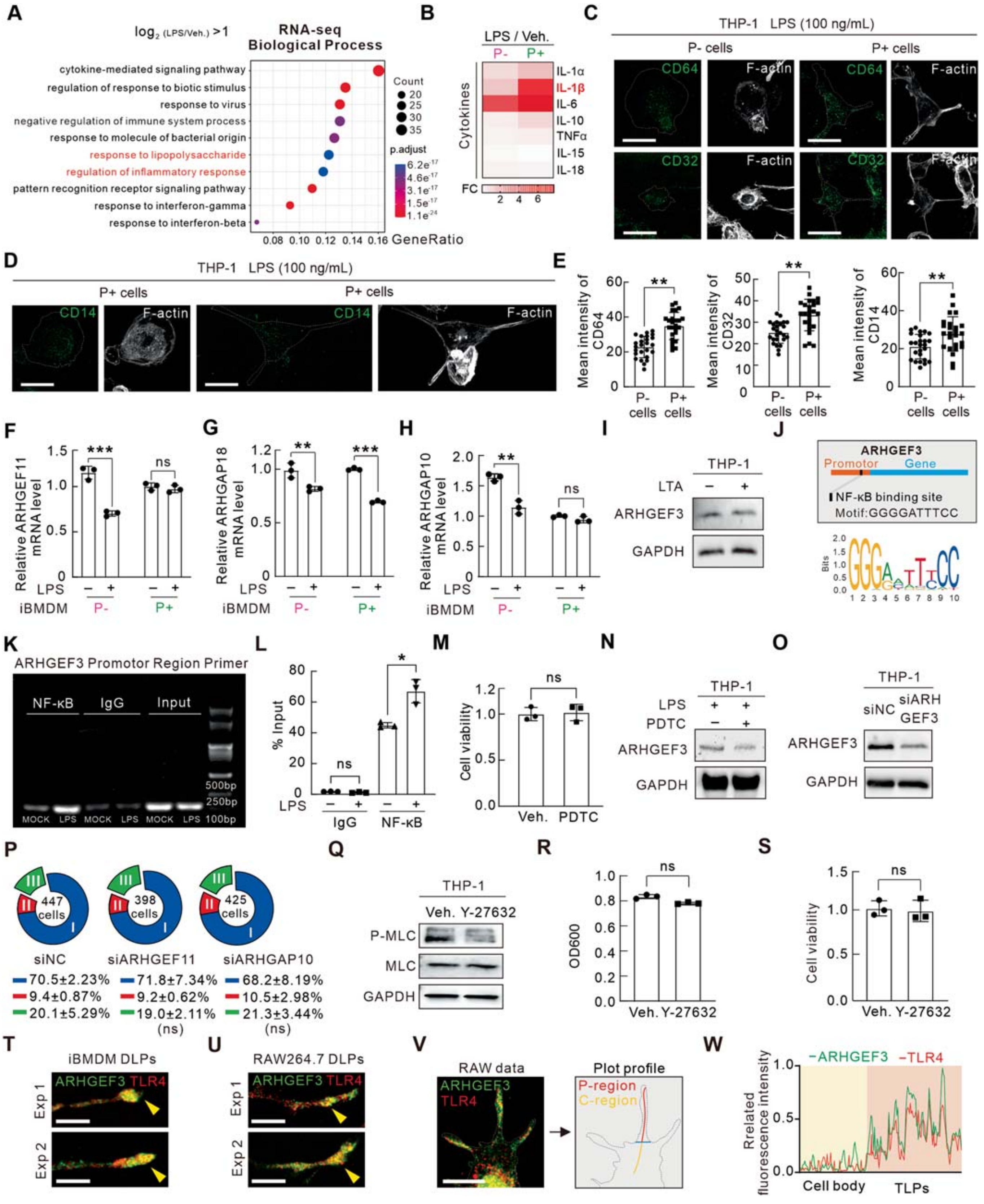

A
log2 (LPS/Veh.) >1
RNA-seq Biological Process
cytokine-mediated signaling pathway
regulation of response to biotic stimulus
response to virus
negative regulation of immune system process
response to molecule of bacterial origin
response to lipopolysaccharide
regulation of inflammatory response
pattern recognition receptor signaling pathway
response to interferon-gamma
response to interferon-beta
GeneRatio
Count
p.adjust
B
LPS / Veh.
P- P+
Cytokines
IL-1α
IL-1β
IL-6
IL-10
TNFα
IL-15
IL-18
FC
C
THP-1 LPS (100 ng/mL)
P- cells
P+ cells
CD64
F-actin
CD32
D
THP-1 LPS (100 ng/mL)
P+ cells
CD14
F-actin
E
Mean intensity of CD64
Mean intensity of CD32
Mean intensity of CD14
P- cells
P+ cells
F
Relative ARHGEF11 mRNA level
G
Relative ARHGAP18 mRNA level
H
Relative ARHGAP10 mRNA level
LPS
iBMDM
ns
I
THP-1
LTA
ARHGEF3
GAPDH
J
ARHGEF3
Promotor
Gene
NF-κB binding site
Motif:GGGGATTTCC
Bits
K
ARHGEF3 Promotor Region Primer
NF-κB
IgG
Input
500bp
250bp
100bp
MOCK LPS MOCK LPS MOCK LPS
L
% Input
IgG
NF-κB
M
Cell viability
Veh.
PDTC
N
THP-1
LPS
PDTC
ARHGEF3
GAPDH
O
THP-1
siNC
siARHGEF3
P
447 cells
398 cells
425 cells
siNC
siARHGEF11
siARHGAP10
70.5±2.23%
9.4±0.87%
20.1±5.29%
71.8±7.34%
9.2±0.62%
19.0±2.11% (ns)
68.2±8.19%
10.5±2.98%
21.3±3.44% (ns)
Q
THP-1
Veh. Y-27632
P-MLC
MLC
GAPDH
R
OD600
Veh. Y-27632
S
Cell viability
Veh. Y-27632
T
iBMDM DLPs
ARHGEF3 TLR4
Exp 1
Exp 2
U
RAW264.7 DLPs
ARHGEF3 TLR4
Exp 1
Exp 2
V
RAW data
ARHGEF3
TLR4
Plot profile
P-region
C-region
W
-ARHGEF3 -TLR4
Rrelated fluorescence intensity
Cell body
TLPs

Figure EV4. ARHGEF3 and RhoA is essential in DLPs formation.

(**A**) Biological process enrichment analysis of significantly upregulated genes in P + /P− iBMDMs challenged with LPS (count with average TPM). The top 10 enriched items are shown in the bubble chart. The colored bubbles from red to blue indicates the descending order of *P*. adjust value. The sizes of the bubbles are displayed from small to large in ascending order of gene counts. The *x* and *y* axis represent the gene ratio and the GO terms, respectively. (**B**) Heatmap shows the transcriptional levels of inflammatory cytokines by RNA sequencing. (**C**) Representative images showing the F-actin visualized by phalloidin and macrophage surface markers CD64 and CD32 in P − and P+ iBMDMs with LPS treatment for 6 h. Bars, 10 μm. (**D**) Representative images showing the F-actin visualized by phalloidin and macrophage surface markers CD14 in P− and P+ iBMDMs with LPS treatment for 6 h. Bars, 10 μm. (**E**) quantification of the mean intensity of macrophage surface markers CD64, CD32, and CD14 in P− and P+ iBMDMs in (**C**) and (**D**). (**F–H**) Quantifications of ARHGEF11, ARHGAP18, ARHGAP10 transcriptional levels by qRT-PCR in P- and P+ iBMDMs upon LPS stimulation. (**I**) Western blot analysis of ARHGEF3 upon LTA treatment. (**J**) Schematic diagram showing predicted NF-κB binding site in the ARHGEF3 promoter. (**K**, **L**) RT-qPCR analysis of DNA pulled down with IgG or NF-κB antibody upon LPS stimulus. (**M**) Quantification of the cell toxicity upon PDTC treatment. (**N**) Western blot analysis of ARHGEF3 upon LPS, or combined LPS and PDTC treatment. (**O**) Western blot analysis demonstrating ARHGEF3 knockdown efficiency in THP-1 macrophages. (**P**) Pie charts show the percentage of three shapes in macrophages with RNAi treatment of negative control, ARHGEF11 and ARHGAP10 and with *Salmonella* infection for 6 h. (**Q**) Western blot analysis of pMLC and MLC upon Y-27632 treatment in THP-1 macrophages. (**R**) Quantification of bacterial growth rate upon Y-27632 treatment. (**S**) Quantification of cell toxicity upon Y-27632 treatment with *Salmonella* infection for 6 h. (**T**, **U**) Representative images of DLPs in iBMDMs (**T**) and RAW264.7 (**U**) stimulated by LPS and stained with ARHGEF3 and TLR4 antibodies. Yellow arrows depict the DLPs. (**V**) The distribution of ARHGEF3 and TLR4 from DLPs to cell body. P-region, the specific pseudopods region; C-region, Cell body region. (**W**) Line profiles of related fluorescence intensity of ARHGEF3 and TLR4 in (**V**). Data are presented as mean ± s.d. from three independent experiments. Dots in quantifications presented individual cells. *P* values from left to right (**E**): $P = 0.00288$, $P = 0.00189$, $P = 0.00425$. *P* values from left to right (**F**): $P = 0.00054$, $P = 0.45742$. *P* values from left to right (**G**): $P = 0.00150$, $P = 0.00067$. *P* values from left to right (**H**): $P = 0.00243$, $P = 0.14849$. *P* values from left to right (**L**): $P = 0.39404$, $P = 0.03801$. ns $P > 0.05$; *$P < 0.05$; **$P < 0.01$; ***$P < 0.001$: one-way ANOVA with Sidak's analysis (**P**), two-way ANOVA with Sidak's analysis (**F–H**, **L**), unpaired two-tailed Student's *t* test (**E**, **M**, **R**, **S**), Benjamini–Hochberg (BH) analysis (**A**). Source data are available online for this figure.

 © The Author(s)

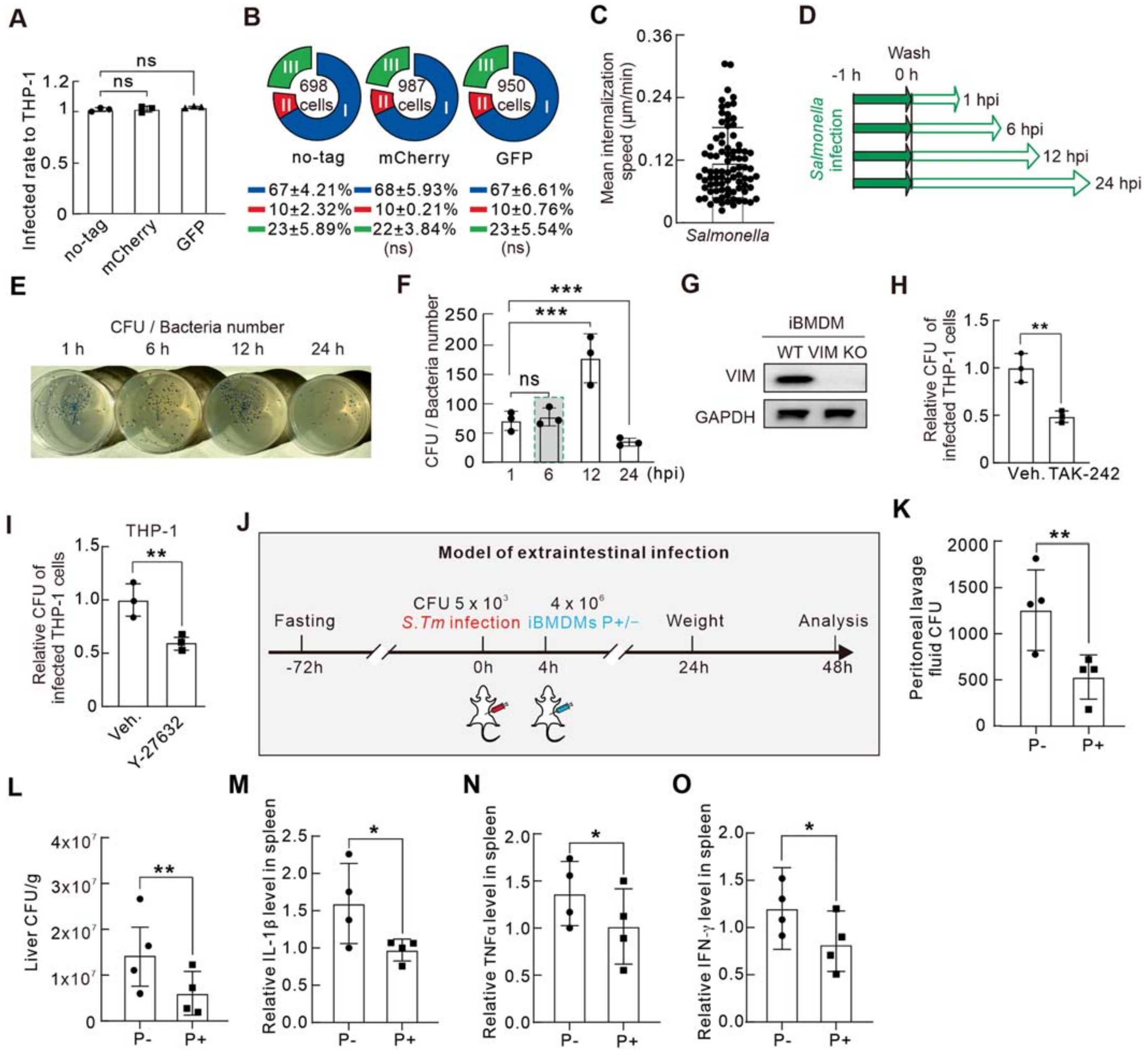

Figure EV5. Cytoskeleton and related signaling are essential in DLPs formation.

(**A**) Quantification of infection rates in THP-1 macrophages with fluorescence-tagged or non-tagged *Salmonella*. (**B**) Pie charts show the percentage of three shapes of macrophages with *Salmonella* infection for 6 h in (**A**). (**C**) Quantification of the average speed of inward moving *Salmonella*. $n = 78$ cells. (**D**) Schematic diagram illustrating the CFU assay performed at distinct infection durations. (**E**) Representative images of the CFU assay at different time point post *Salmonella* infection of THP-1 macrophages. (**F**) Quantification of intracellular *Salmonella* over time in THP-1 macrophages. (**G**) Western blot analysis of vimentin in WT and VIM KO iBMDM. (**H**, **I**) Quantification of the bacterial load upon *Salmonella* infection in THP-1 macrophages treatment with TAK242 or Y-27632. (**J**) Schematic diagram of extraintestinal infection mouse model incorporating macrophage re-infusion. (**K**, **L**) Quantification of bacterial load indicated by colony forming unit (CFU) in peritoneal lavage fluid (**K**) and liver (**L**) infected with *Salmonella*. (**M–O**) Quantitative RT-qPCR analysis of the expression levels of inflammatory markers in the spleen. Data are presented as mean ± s.d. from three independent experiments. Dots in quantifications presented individual cells. *P* values from left to right (**F**): $P = 0.65388$, $P = 0.00094$, $P = 0.00094$. $P = 0.00558515$ (**H**). $P = 0.00124$ (**I**). $P = 0.00805$ (**K**). $P = 0.00243$ (**L**). $P = 0.03700$ (**M**). $P = 0.04913$ (**N**). $P = 0.04496$ (**O**). ns $P > 0.05$; $^{*}P < 0.05$; $^{**}P < 0.01$; $^{***}P < 0.001$: unpaired two-tailed Student's *t* test (**H**, **I**, **K–O**), one-way ANOVA followed by a Tukey's post hoc test (**A**, **F**). Source data are available online for this figure.

© The Author(s)
